# A vector-based strategy for olfactory navigation in *Drosophila*

**DOI:** 10.1038/s41586-026-10827-7

**Published:** 2026-07-22

**Authors:** Andrew F. Siliciano, Sun Minni, Chad Morton, Charles K. Dowell, Noelle B. Eghbali, Silas E. Busch, Juliana Y. Rhee, L. F. Abbott, Vanessa Ruta

**Affiliations:** 1 Laboratory of Neurophysiology and Behavior and Howard Hughes Medical Institute, The Rockefeller University, New York, NY USA; 2 Mortimer B. Zuckerman Mind Brain Behavior Institute, Kavli Institute for Brain Science, Department of Neuroscience, Columbia University, New York, NY USA

**Keywords:** Learning and memory, Neural circuits, Olfactory system

## Abstract

For many species, odours serve as key navigational cues^1,2^. Although tracking an odour plume has been modelled as a reflexive process^3–5^, it remains unclear whether animals can use memories of their past odour encounters to infer the spatial structure of their chemical environment or their location within it. Here we developed a virtual-reality olfactory paradigm that allows head-fixed *Drosophila* to explore structured chemical landscapes, offering insight into how memory mechanisms shape their navigational strategies. We found that flies track an appetitive odour corridor by following its boundary, alternating between rapid counter-turns to exit the plume and directed returns to its edge. Using a combination of behavioural modelling, functional calcium imaging and neural perturbations, we show that this ‘edge tracking’ strategy relies on vector-based computations within the *Drosophila* central complex, in which flies store and dynamically update memories of the direction to return to the plume’s boundary. Consistent with this, we find that FC2 neurons within the fan-shaped body, which encode a fly’s navigational goal^6^, signal the direction back to the odour boundary when flies are outside the plume. Plume tracking thus engages components of a conserved navigational toolkit, in which flies can use directional memories to navigate through complex and shifting chemical landscapes.

## Abstract (web summary)

Using a virtual reality system, experiments in *Drosophila* show that flies use memory mechanisms to navigate along the boundary of an odour plume and track the source of a smell.

## Main

Navigating within a complex environment is a ubiquitous requirement for all animals, driving the evolution of diverse behavioural strategies tailored to the structure and stability of sensory landscapes^1^. In many instances, a simple chain of sensorimotor reflexes can be used to guide animals towards or away from a salient object using only immediate sensory cues^1,7^. However, when sensory signals are intermittent or unreliable, animals might instead need to navigate towards goals that cannot be continuously sensed, requiring memory of their position or direction. Insects, for example, are thought to use a vector-based navigational strategy in which they integrate both distance and directional information to guide them towards a stored location^8,9^. This approach is exemplified by the navigational feats of central place foragers, such as ants and bees, which can wander far from their nests and return efficiently even in a mostly featureless environment^9,10^. However, whether similar vector-based strategies can be used to steer through more dynamic sensory environments, such as an odour plume wafting from its source, has remained unclear^7,11^.

Odours are among the most salient signals that insects use to guide navigation^2^. Plume tracking in insects has been proposed to rely on a mainly reflexive strategy, in which animals surge upwind after encountering an odour and cast crosswind after losing contact with the plume to re-encounter it^3–5,12^. However, the dynamic nature of olfactory plumes often requires animals to track using only intermittent odour encounters^11,13^ separated by vast stretches of clean air that offer no immediate sensory information to point them back to the plume. Memory mechanisms could allow animals to bridge across these fragmented odour experiences. Here we investigate this possibility by developing a virtual-reality paradigm for plume navigation and show that *Drosophila* store the direction required to return to the plume as an angular goal that is continuously updated as they track along the plume’s border. Consistent with the notion that a plume is not a fixed spatial landmark, flies appear to store only the direction towards its boundary, not their distance from it. Our results suggest that elements of an evolutionarily conserved navigational toolkit^14^ can be flexibly deployed in different sensory contexts, allowing flies to use odours as chemical signposts to track long distances towards a remote source.

## Flies track a plume along its boundary

**Fig. 1:**
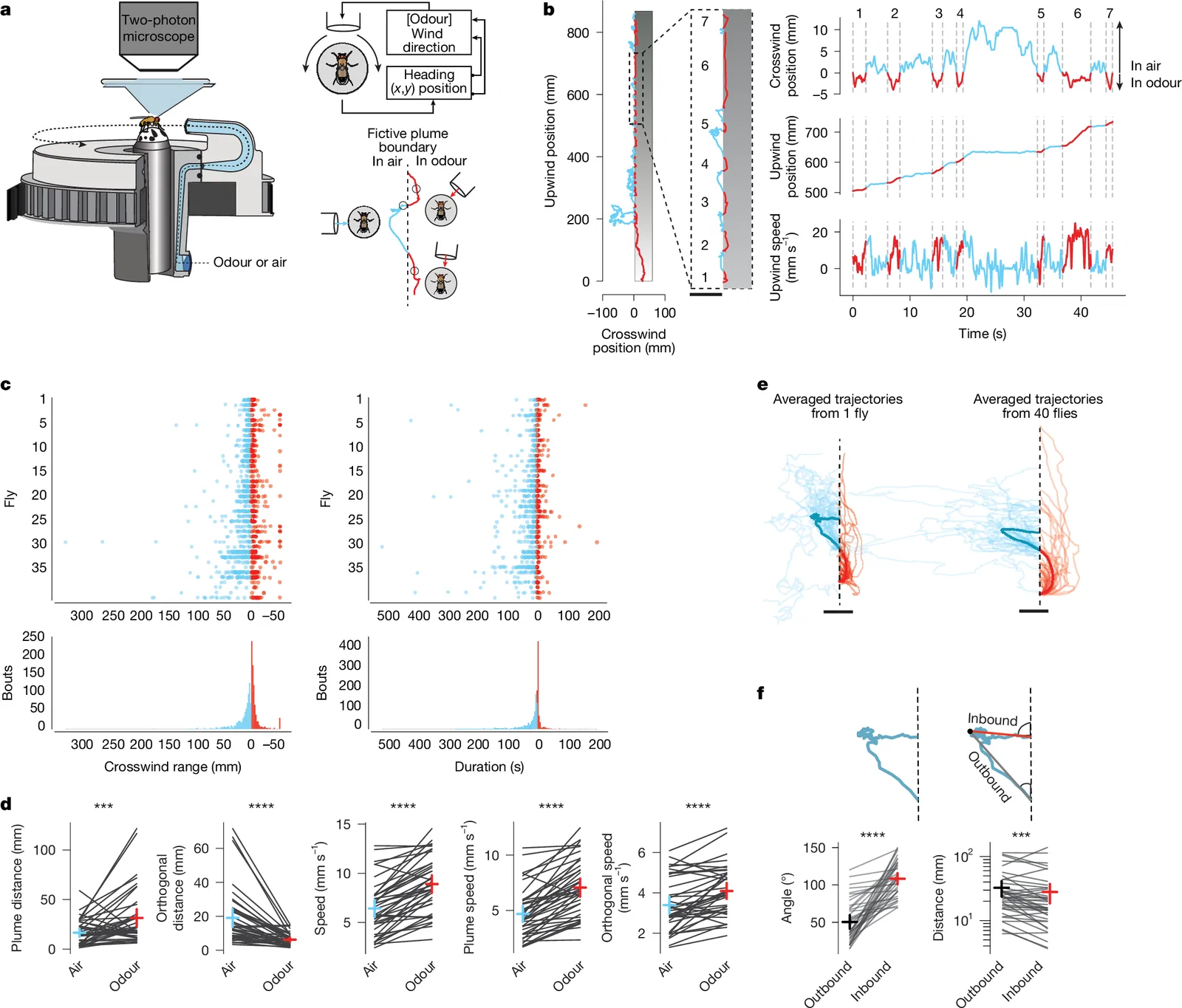
Flies track a plume along its boundary. **a**, Left, schematic of the closed-loop virtual-reality system. A tethered fly walks on an air-supported ball while its heading controls the angular position of a nozzle delivering a constant airstream, with or without odour. Top right, the fly’s fictive position determines the odour concentration. Bottom right, nozzle position as the fly crosses the fictive plume boundary (dashed line). The trajectory is red inside the plume and blue outside it. **b**, Left, example trajectory of a fly tracking an odour corridor with an ascending gradient (10–100% ACV over 1 m); red inside the plume, blue outside. The boxed segment is expanded on the right. Scale bar, 25 mm. Right, crosswind position, upwind position and upwind speed for the inside and outside bouts in the expanded segment. **c**, Distributions of crosswind distance (left) and duration (right) for inside (red) and outside (blue) bouts. Each dot is one bout; *n* = 40 flies. **d**, Average behavioural metrics for bouts inside (red) or outside (blue) the plume. Plume distance and orthogonal distance refer to distance travelled along, and orthogonal to, the longitudinal plume axis. Thin lines, per-fly averages; thick lines with error bars, mean ± s.e.m. across flies. *n* = 40 flies. **e**, Aligned inside (red) and outside (blue) trajectories. Left, all trajectories (thin) and scaled average (thick) for the fly in **b**. Right, scaled averages for 40 individual flies (thin lines) and across-fly average (thick). Scale bars, 10 mm. **f**, Top, schematic of outbound (black) and inbound (red) segments of an outside trajectory, defined relative to the farthest-crosswind point (Methods). Bottom, quantification of the angles and path lengths (log scale) between each segment and the plume. Thin lines, per-fly averages; thick with error bars, mean ± s.e.m. *n* = 40 flies. ***P < 0.001; ****P < 0.0001. See Supplementary Table 1 for full statistics and sample sizes. The schematic in **a** is adapted with permission from ref. ^15^ (Cell Press).

One fundamental challenge to elucidating the behavioural algorithms that underlie plume navigation is that odours are invisible and often carried along turbulent airflow^11,13^, limiting experimental access to an animal’s true sensory experience. To overcome this, we developed a closed-loop olfactory paradigm that allows tethered *Drosophila* to navigate virtual chemical landscapes. In this system, the heading of a fly walking on an air-supported ball is yoked to the rotation of a nozzle carrying a constant airstream, enabling the fly to control its orientation relative to a wind source blowing from a constant allocentric direction^15,16^ (Fig. 1a and Supplementary Video 1). High-speed mass flow controllers dynamically control the concentration of odour infused into the airstream at each moment depending on the fly’s position within this virtual world, allowing us to construct plumes with precise spatial structures. Flies walk in complete darkness, such that aside from proprioceptive feedback to stabilize their trajectories, the wind direction provides the only external directional cue available to orient them.

**Extended Data Fig. 1:**
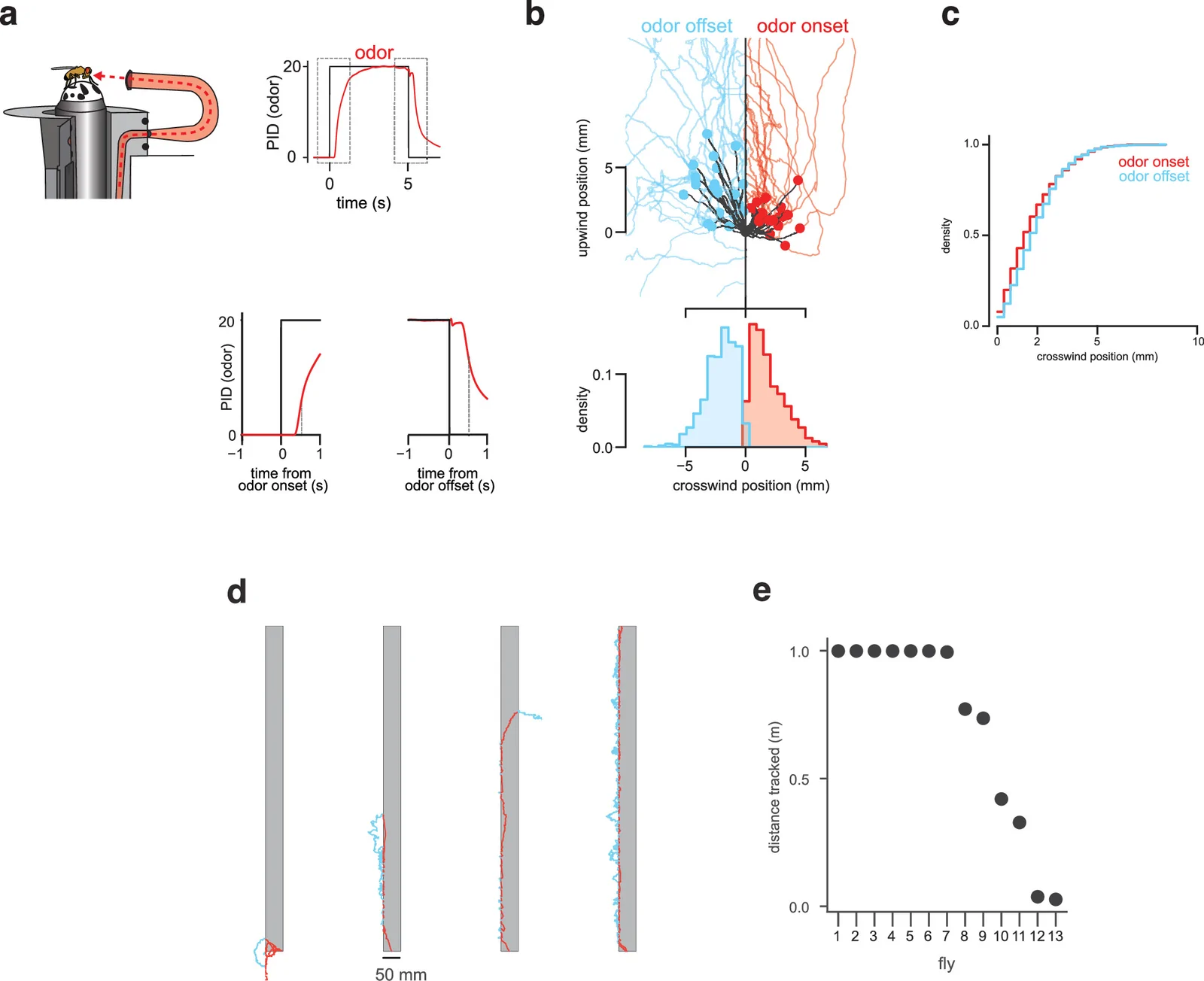
Characteristics of odour stimulation at the plume’s boundary and edge tracking in naive flies. (**a**) Left: Schematic of closed-loop olfactory system highlighting the nozzle yoked to the fly’s heading that provides a continuous stream of clean air that is infused with odour (ACV) when the fly is inside the fictive plume boundary. Right: odour concentration profiles measured by PID (photoionization detector) over time at the position of the fly. Top panel shows the full duration of an odour pulse with an onset and decay of approximately 500 msec. Bottom panels show zoomed-in views of the PID trace over the first second from odour pulse onset (left) and offset (right). (**b**) Positional data of a representative fly upon odour onset and offset. Top: the position of the fly for all entries into the plume (red) and exits from the plume (blue). The solid points represent the fly’s position 500 msec after odour onset and offset. Bottom: histogram shows the distribution of crosswind positions of the fly upon exiting the plume (blue) and upon entering the plume (red). Owing to the finite time required for the odour to reach the fly as it walks in virtual space shown in (a), the plume’s edge is not perfectly sharp, with a spatial gradient that depends on how fast the fly is walking. (**c**) Cumulative density functions (CDFs) of crosswind positions for flies in response to plume entry (red) and exit (blue). n = 40 flies, 731 inside trajectories and 796 outside trajectories. (**d**) Representative trajectories of four naive flies that had never previously encountered an odour corridor tracking a 1-metre vertical plume. Epochs outside the plume are depicted in blue and epochs inside the plume are depicted in red. (**e**) Plume distance tracked by individual naive flies shows that while individual variation exists, most flies readily track the plume. Trials were terminated once a fly tracked more than 1 metre upwind, more than 500 mm in the crosswind direction or more than 100 mm downwind after encountering the plume. Note that for all experiments, flies were starved >24 h. The schematic in **a** is adapted with permission from ref. ^15^ (Cell Press).

We reasoned that memory mechanisms facilitating navigation might be most apparent using a stationary plume, the stability of which allows flies to accumulate predictive information about its structure. We therefore first investigated how flies track a 50-mm-wide fictive ‘corridor’ of the appetitive food odour apple cider vinegar (ACV) aligned to the wind direction (Fig. 1b)—a simplified chemical landscape that nevertheless captures key features of the slowly dispersing surface plumes that walking flies encounter in nature when close to their source^17^. The corridor was bounded on both sides, such that if a fly walked laterally in one direction or the other, it would exit the plume and the ACV concentration would dissipate rapidly (Fig. 1a,b and Extended Data Fig. 1a–c). Owing to the approximately 500-ms delay for odour entering the airstream to reach the fly’s antenna, the lateral boundaries of the plume were not perfectly sharp but decayed within about 5 mm, depending on the fly’s walking speed (Extended Data Fig. 1b). A shallower gradient ran along the corridor’s longitudinal direction, such that a fly experienced a steadily increasing concentration of ACV (10–100% over 1 m) as it walked upwind, mimicking the sensory feedback of approaching the odour source.

Previous descriptions of plume tracking suggest that flies navigate this corridor through odour-gated anemotaxis—surging upwind after contacting the odour, while remaining within the plume’s boundaries^1,3–5,18^. However, we found that flies reliably ascended the length of the plume by tracking along a single edge, through repeating two behavioural motifs: after entering the corridor, they counter-turned rapidly to exit it, and once outside the plume they performed a more circuitous exploration before re-entering (Fig. 1b and Supplementary Video 2). We call this behaviour ‘edge tracking’. Although this strategy is reminiscent of previous descriptions of insects tracking the borders of surface plumes^18^ or odour patches^19^, the precision of our experimental paradigm offers an inroad to examine how flies use this boundary information for navigation.

During edge tracking, flies travelled inside the plume only briefly (mean 4.9 s, *n* = 755 bouts), instead spending significantly more time outside the odour corridor as they ascended upwind along its boundary (Fig. 1c). Nevertheless, most of their progress occurred during these brief inside bouts (Fig. 1b,d). On average, flies also progressed upwind when they walked outside the plume (Fig. 1b,d), but these trajectories were much more variable in both distance and duration, with some excursions extending hundreds of millimetres orthogonal to the plume’s boundary before returning (Fig. 1c,d). Flies typically tracked along a single edge, walking within the plume’s lateral margin (mean 10.4 mm) and rarely crossing the plume’s width to the other edge (<4%, *n* = 793 bouts). Most flies that initially encountered an ACV corridor tracked its edge spontaneously for hundreds of millimetres (Extended Data Fig. 1d,e), underscoring the robustness of this strategy.

**Extended Data Fig. 2:**
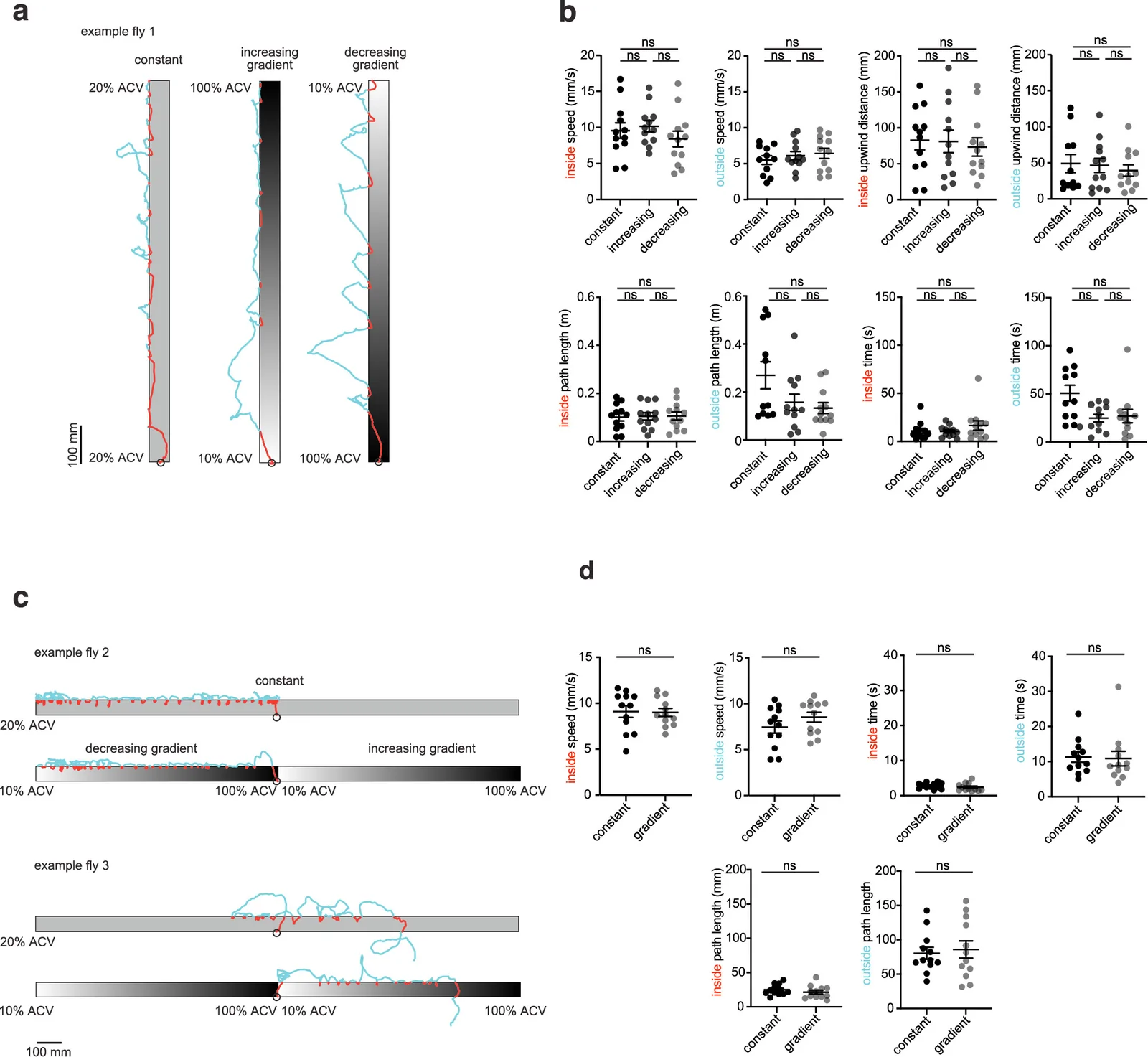
Edge tracking does not depend on the concentration gradient along the longitudinal axis of the plume. (**a**) Representative trajectories of an individual fly tracking an odour corridor of a constant concentration of 20% ACV (left), a plume with an increasing ACV concentration gradient in the upwind direction (10–100% ACV over 1 metre, middle) and a plume with a decreasing ACV concentration gradient in the upwind direction (100-10% ACV over 1 metre, right). Start of each trajectory is marked with a circle. (**b**) Comparison of average behavioural metrics for the three plumes shown in (**a**). Each fly navigated a constant concentration, increasing gradient and decreasing gradient plume in a randomized order across flies. Each point represents the average metrics for a single fly, with interindividual average and SEM shown (n = 12 flies). Wilcoxon signed rank test. (**c**) Representative trajectories for two flies that sequentially navigated a constant concentration 90° plume (top) and 90° plume, in which the fly was positioned at the centre and could select to walk up an increasing (10–100% ACV over 1 metre) or decreasing gradient (100-10% ACV over 1 metre, bottom). (**d**) Comparison of average behavioural metrics for the constant or gradient plumes shown in (c). Each point represents the average metrics for a single fly, with interindividual average and SEM shown (n = 12 flies). n.s. not significant, p > 0.05. Details of statistical analyses and sample sizes are given in Supplementary Table 1.

**Extended Data Fig. 3:**
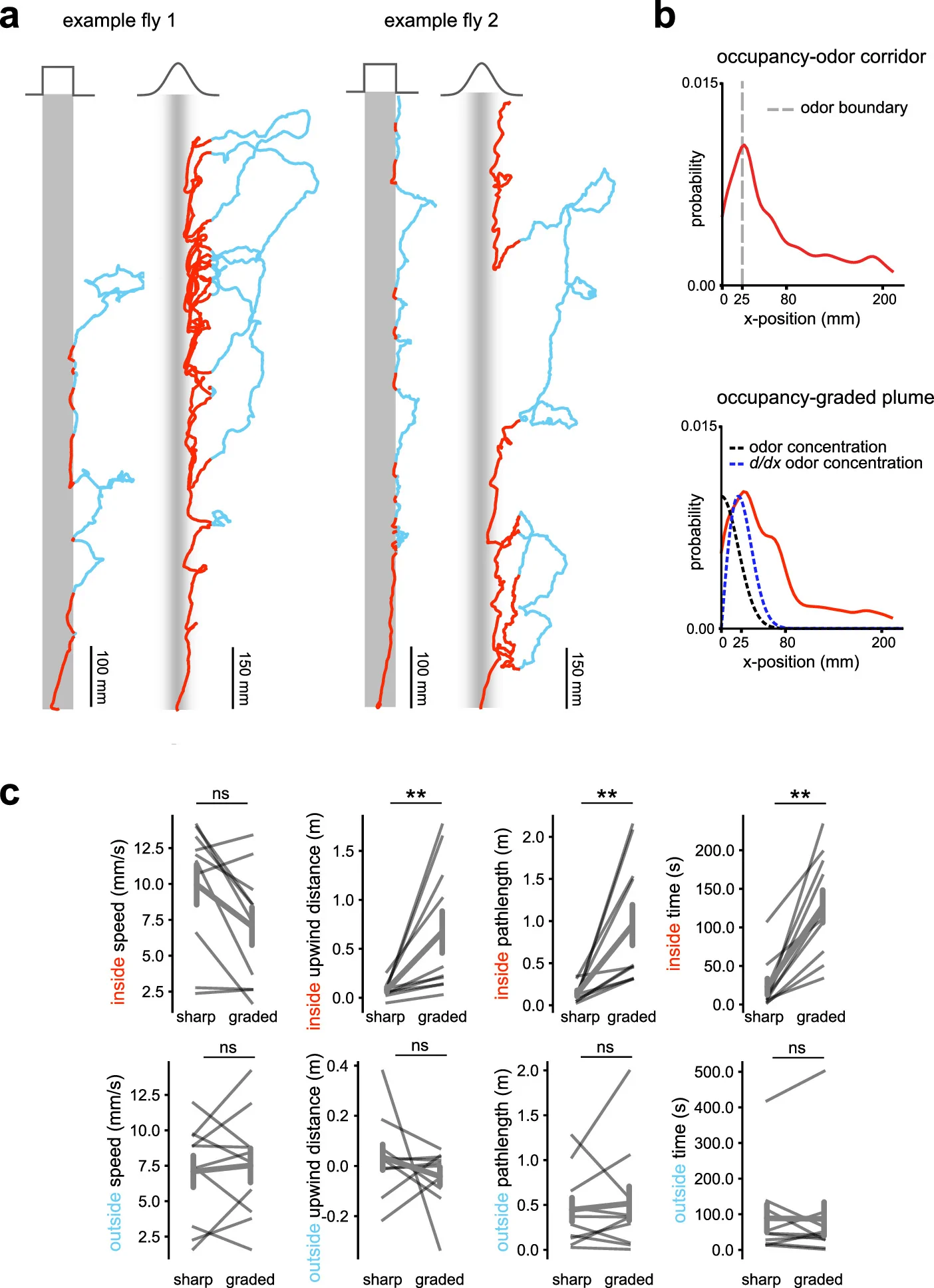
Flies track odour corridors with smoothly graded lateral concentration profiles. (**a**) Representative trajectories from flies that each tracked two odour corridors with the same longitudinal structure, but either sharp lateral boundaries (left) or graded lateral boundaries that followed a Gaussian profile (right). Red traces show trajectories inside the plume; blue traces show trajectories outside the plume. Shading indicates the lateral odour profile for each plume type. For both plumes, peak odour concentration at centre line was 20% ACV. (**b**) Comparison of occupancy as a function of distance perpendicular to an odour corridor (top) or a graded plume (bottom). Flies tracking a graded plume concentrated their trajectories along an offset corresponding to the derivative of the concentration change (blue). (**c**) Comparison of average behavioural metrics for flies tracking an odour corridor with sharp odour boundaries or a graded plume. Each thin line represents the average metrics for a single fly, with thick line representing interindividual average and SEM (n = 10 flies). ** p < 0.01; n.s. not significant, p > 0.05. Details of statistical analyses and sample sizes are given in Supplementary Table 1.

Animals can use perceived changes in odour concentration to localize an odour source^1^. However, flies tracked corridors of a constant odour concentration (20% ACV) as effectively as they did those with an increasing longitudinal gradient, and even tracked corridors in which the longitudinal gradient was reversed, such that they encountered a progressively lower ACV intensity as they advanced upwind (Extended Data Fig. 2a). On average, fly trajectories were indistinguishable across these plumes (Extended Data Fig. 2b). Flies thus do not rely on gradual changes in odour concentration along the plume’s length to edge track, suggesting that the plume’s lateral boundary—where odour gradients are steepest—is the more salient spatial cue. Consistent with this, flies readily tracked plumes with a Gaussian lateral profile characteristic of time-averaged plumes^20^, concentrating their trajectories along the isocline of steepest concentration change (Extended Data Fig. 3). This behaviour aligns with the sensitivity of *Drosophila* olfactory pathways to concentration derivatives^21,22^ and suggests that, even when plumes lack sharp edges, flies track an effective boundary defined by maximal lateral contrast.

**Extended Data Fig. 4:**
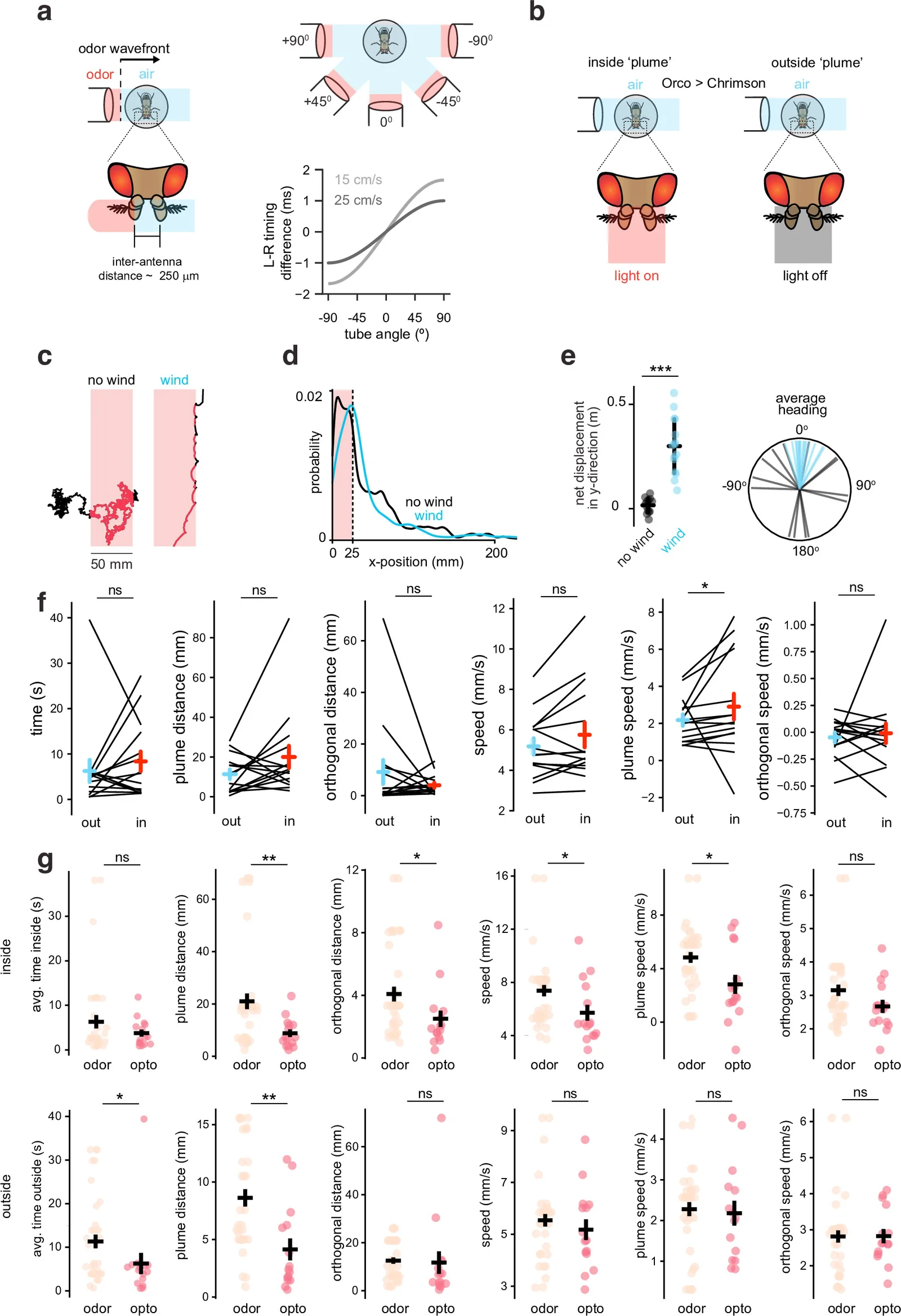
Flies do not seem to require bilateral signals to localize the plume’s edge or to track along its boundary. (**a**) Schematic of the closed-loop olfactory stimulation paradigm illustrating the contact timing of an odour wavefront arriving from different angles (+90°, +45°, 0°, −45°, −90°) to each antenna. Given the small spatial distance between the two antennae and the wind speed, the difference in the timing of odour arrival is <2 ms even when flies are walking in the crosswind direction. (**b**) Schematic of flies navigating a fictive odour plume generated by optogenetic activation of Orco^+^ olfactory sensory neurons expressing CsChrimson. When a fly is inside the boundaries of the fictive plume (left), both antennae are simultaneously stimulated using 660 nm light. Outside the fictive plume (right), the light is off (grey) and there is no stimulation. Wind is maintained in closed loop throughout. (**c**) Representative trajectories of a fly presented with a 660 nm light corridor in the absence (left) or presence (right) of wind. Paths inside the corridor are shown in red while paths outside the corridor are shown in black. (**d**) Probability distributions of the absolute value of flies’ x-positions inside a light corridor (depicted in red) in the presence (blue) or absence of wind (black). n = 14 flies (no wind), 15 flies (wind). (**e**) Left: Average displacement in the y-direction for flies presented with a light corridor in the presence (blue) or absence of wind (black). Right: Average heading inside the light corridor in the presence (blue) or absence of wind (black). n = 14 flies (no wind), 15 flies (wind). (**f**) Comparison of the average behavioural metrics for all bouts inside the optogenetic light corridor (red) or outside the corridor in clean air (blue). Each line represents the average for all inside or outside bouts for an individual fly, with the average across flies ±SEM. shown in red or blue. n = 15 flies. (**g**) Comparison of behavioural metrics for flies tracking a real odour corridor (n = 40, same flies as in Fig. 1) or a fictive optogenetic corridor (n = 15 flies). Each dot represents the average metrics for an individual fly with average across flies ±SEM. shown in black. ***p < 0.001; **p < 0.01; *p < 0.05; n.s. not significant, p > 0.05. Details of statistical analyses and sample sizes are given in Supplementary Table 1. The schematics in **a**,**b** are adapted with permission from ref. ^15^ (Cell Press).

Insects have been proposed to track the boundaries of surface plumes using bilateral comparisons of their sensory appendages^23–25^. In our assay, however, plume boundaries are not defined spatially but arise from rapid temporal changes in odour concentration, resulting in nearly synchronous odour delivery to both antennae (Extended Data Fig. 4a). To assess whether flies use transient sensory asymmetries for edge tracking, we expressed the optogenetic activator CsChrimson in most olfactory sensory neurons using the *Orco* promoter, allowing for simultaneous activation of both antennae as flies crossed into a 660-nm light corridor (Extended Data Fig. 4b). In the absence of wind, *Orco>CsChrimson* flies preferentially remained within the light corridor (Extended Data Fig. 4c,d)—consistent with the intrinsically appetitive nature of broad olfactory sensory neuron activation^24,26^—but progressed minimally along the plume’s length (Extended Data Fig. 4e). However, adding a closed-loop wind source led *Orco>CsChrimson* flies to advance upwind along the boundary of the light corridor using a similar behavioural strategy to edge tracking (Extended Data Fig. 4c,f,g). Flies thus do not seem to require a bilateral comparison of olfactory input to localize the plume’s boundary. Rather, they rely on wind direction and the steep change in odour concentration at the lateral boundary to edge track.

## Returns to the plume are not random

During edge tracking, the wind provides the sole external landmark available to flies to orient themselves but provides no information to guide returns back to the plume. Nevertheless, although many flies in our paradigm adhered closely to the plume’s edge, with only transient outside excursions, others occasionally performed long trajectories that carried them hundreds of millimetres away (Fig. 1c). During these long excursions, flies were immersed in clean air for tens of seconds before returning to the edge, frequently along shorter, more directed paths (Fig. 1c,e), suggesting that they retain information about the direction of the plume. Consistent with this, outside trajectories were asymmetrical, with flies leaving the plume at a shallow angle, but returning angled almost perpendicularly to the plume’s edge along shorter, more direct paths (Fig. 1e,f).

**Extended Data Fig. 5:**
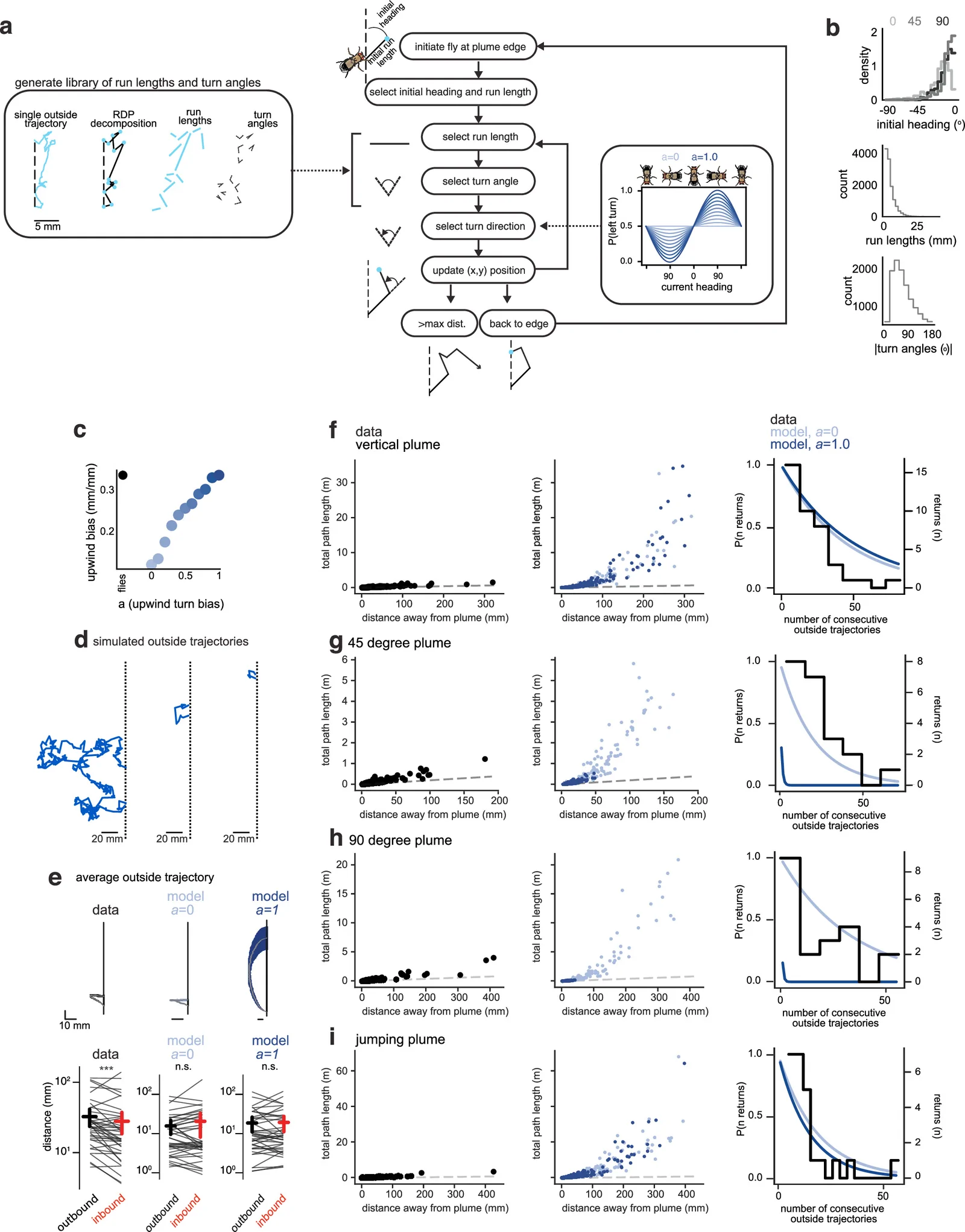
Edge tracking is not the result of random exploration outside the plume. (**a**) Schematic of random-walk model. Left: individual outside trajectories from 40 flies tracking a vertical plume were decomposed into a series run lengths and turn angles using the Ramer-Dougles-Peucker algorithm to generate a library for generating synthetic trajectories with the same underlying statistics. Right: flowchart of how simulated trajectories were generated from this library. The fly was initiated at the plume’s edge with an initial heading and run length matching the distribution observed during actual fly exits from the plume. At each step, a run length, turn angle, and turn direction was chosen to update the new position (x,y) of the fly. The procedure was repeated until the simulated trajectories returned to the edge or travelled greater > 500 mm orthogonal to the plume’s edge. The turn direction was biased by the wind direction as shown in the inset graph which compares the heading distributions for different upwind biases (*a* = 0 to *a* = 1.0). (**b**) Top: Distribution of initial headings for the three different plume geometries: 0° (light grey); 45° (dark grey) and 90°(black). Middle: distribution of run lengths derived from the library of trajectories. Bottom: distribution of turn angles derived from the library of trajectories. (**c**) Relationship between upwind bias and upwind turn bias (*a*) for simulated and real flies. Upwind bias is defined as the distance travelled in the upwind direction divided by total path length. Note that real flies show an upwind bias of 0.35, which corresponds to an upwind turn bias (*a* = 1.0). (**d**) Examples of simulated outside trajectories with an upwind bias (*a* = 1), highlighting that while all three return to the plume’s boundary, longer trajectories become increasingly inefficient and take extended path lengths to do so. (**e**) Average outside trajectories for flies (left) and simulated trajectories (right) tracking a vertical plume with different upwind biases (*a* = 0, *a* = 1). Note that simulations with *a* = 1.0 make progress in the upwind direction but lack directed returns. Because only simulated trajectories that successfully returned to the plume are included in these averages, they progressed further upwind than real flies but were less efficient as shown in (d). Bottom: Comparison of the inbound and outbound segments of outside trajectories (see Methods), defined as leading away from (outbound, black) or returning to (inbound, red) the point farthest from the plume in the crosswind direction for real flies (replotted from Fig. 1f), and simulated trajectories lacking an upwind bias (*a* = 0) and with an upwind bias (*a* = 1.0). Each thin line represents the average for all trajectories of an individual fly, with the interindividual average ±SEM. shown. (**f**–**i**) Comparison of real and simulated trajectories for different plume configurations: vertical (0°) plume (**f**), 45° plume (**g**), 90° plume (**h**), and jumping plume (**i**). Comparison of total path length as a function of distance in the perpendicular direction away from the plume for real flies (left column), model with *a* = 0 (light blue dots) or *a* = 1.0 (dark blue dots). The dashed grey line denotes optimal efficiency—two times the distance away from the plume—representing trajectories that go straight out and back. Right column compares the probability of returning for *n* consecutive outside bouts for real flies in black and random models shown in dark and light blue. Note that the likelihood that a random walk will return to the plume’s boundary decreases with increasing numbers of consecutive bouts, especially when accounting for a fly’s intrinsic upwind drive (a = 1.0, dark blue). In contrast, flies (black) are more effective than a random walk. n.s. not significant; ****p < 0.0001. Details of statistical analyses and sample sizes are given in Supplementary Table 1. The schematic in **a** is adapted with permission from ref. ^15^ (Cell Press).

The observation that flies take lengthy excursions away from the plume and make directed returns suggests that their outside exploration is not random. To examine this, we decomposed a fly’s outside trajectories into their elementary run lengths and turn angles (Extended Data Fig. 5a,b), and then randomly drew from these experimental distributions to generate synthetic trajectories built from the same path statistics. Simulated trajectories could reliably return to the plume’s boundary (Extended Data Fig. 5d) but were highly circuitous, with significantly longer path lengths compared with those of real flies (Extended Data Fig. 5f). Moreover, the segments away from and back to the plume were equivalent in length, in contrast to the more directed returns real flies display (Extended Data Fig. 5e). Incorporating the intrinsic upwind bias that flies exhibit resulted in simulated trajectories that ascended the plume but remained highly inefficient (Extended Data Fig. 5c,e), consistent with the fact that returning to a vertical odour corridor depends on progress in only the crosswind—not the upwind—direction. Edge tracking therefore does not adhere to the statistics of a random search process—rather, the directed returns that flies perform suggest that they rely on a memory of past plume encounters to guide efficient trajectories back to the boundary.

## Flies track diverse plume geometries

**Fig. 2:**
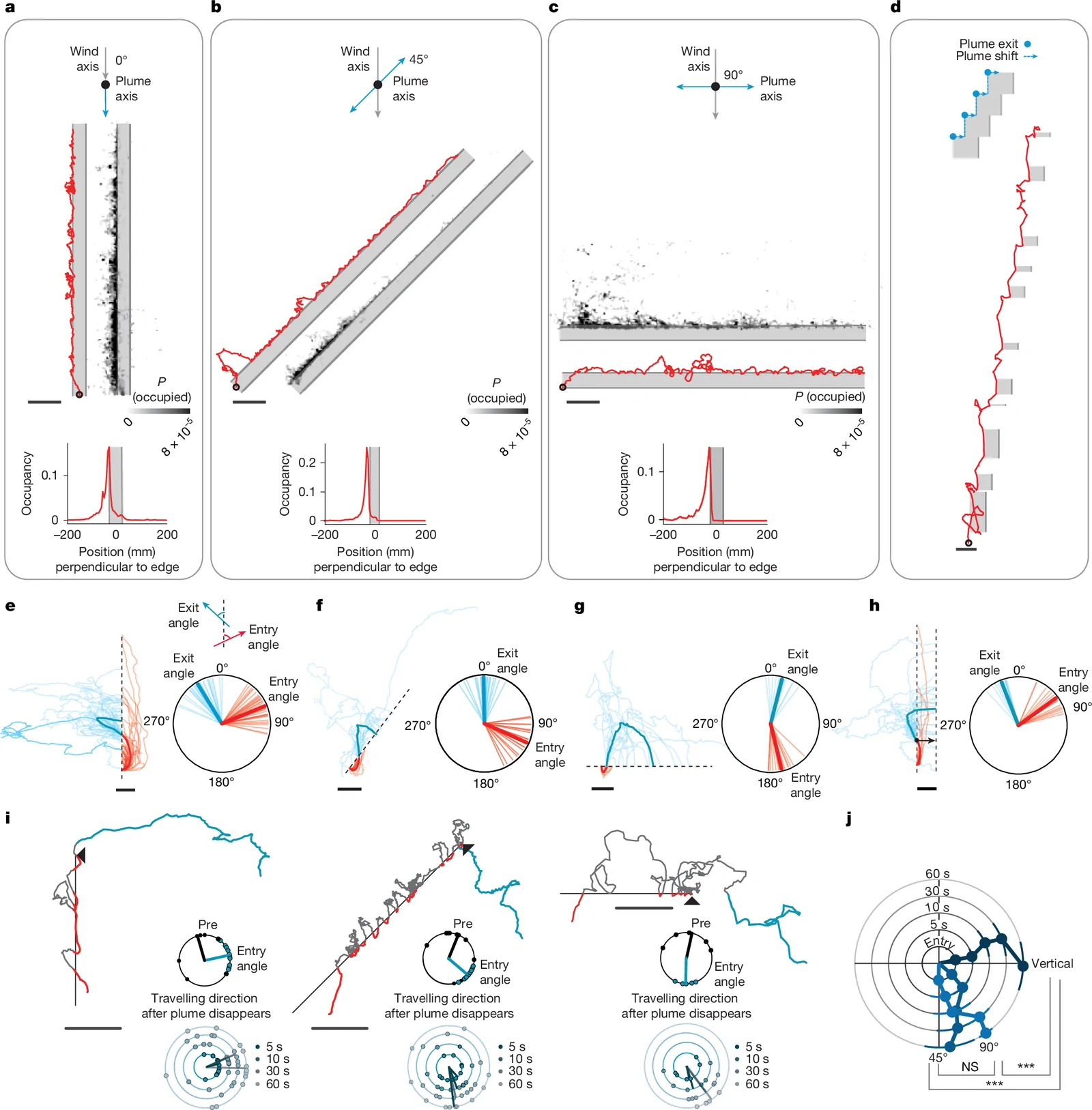
Flies track plumes that are not aligned with the wind. **a**–**c**, Flies track plumes with different orientations relative to the wind. For each, we show a representative trajectory (red, starting position denoted by black circle) and occupancy as a function of distance to the plume. **a**, Vertical plume (*n* = 40 flies). **b**, 45° plume (*n* = 21 flies). **c**, 90° plume (*n* = 20 flies). **d**, Representative trajectory of a fly navigating a ‘jumping’ plume in which the plume shifts 20 mm rightwards each time the fly exits. Scale bars, 100 mm (**a**–**d**). **e**–**h**, Left, aligned and averaged inside and outside trajectories for each plume orientation; thin lines, scaled averages for individual flies; thick line, mean across flies. Right, distributions of entry and exit angles (mean heading in the 0.5 s before and after crossing the plume boundary); thin lines, averages for individual flies; thick line, mean across flies. **e**, Vertical plume (*n* = 40 flies). **f**, 45° plume (*n* = 21 flies). **g**, 90° plume (*n* = 20 flies). **h**, Jumping plume (*n* = 24 flies). Scale bars, 10 mm (**e**–**g**); 20 mm (**h**). **i**, Representative trajectories after the plume vanishes during an outside bout for 0°, 45° and 90° plumes. The plume boundary is indicated by the black line, with inside bouts in red and outside bouts in grey. The black arrowhead marks the point of the plume’s disappearance, with the subsequent 2-min trajectory in blue. For each plume, the top polar plot shows the distributions of the average heading as flies walk in wind in the 2 min preceding tracking (pre; black) and the average entry angle during tracking (blue). The bottom polar plot shows the average heading at the indicated times after plume disappearance; each dot is one fly. 0°, *n* = 12; 45° *n* = 11; 90° *n* = 6. Scale bars, 100 mm. **j**, Comparison of the average entry angle during tracking (innermost circle) and travel direction at the indicated times after plume disappearance for flies in **i**. Each circle reflects a time bin; mean ± s.e.m. NS, not significant; ***P < 0.001. Statistical details and sample sizes are in Supplementary Table 1.

Transient shifts in wind direction can cause a plume to meander, such that it no longer aligns with the wind direction or points to the odour source^18,27^. Reflexively surging upwind after encountering an odour would inevitably cause animals to walk out of a meandering plume, suggesting that it could be advantageous to track the boundary of a plume rather than to follow the wind direction^28^. To examine this possibility, we constructed a set of ‘tilted’ plumes of constant ACV concentration, in which the longitudinal axis of the odour corridor was offset from the wind direction by 45° or 90° (Fig. 2a–c). We found that flies robustly tracked the boundaries of tilted plumes for hundreds of millimetres, even for the 90° plume, which required individuals to choose a left or right crosswind direction and advance along it, moving orthogonal to the wind with minimal backtracking. Indeed, flies even tracked a 90° plume along a diminishing concentration gradient (Extended Data Fig. 2c,d), underscoring that their progression along the plume’s edge appears to rely on an internal sense of direction and not on the instantaneous odour concentration.

**Extended Data Fig. 6:**
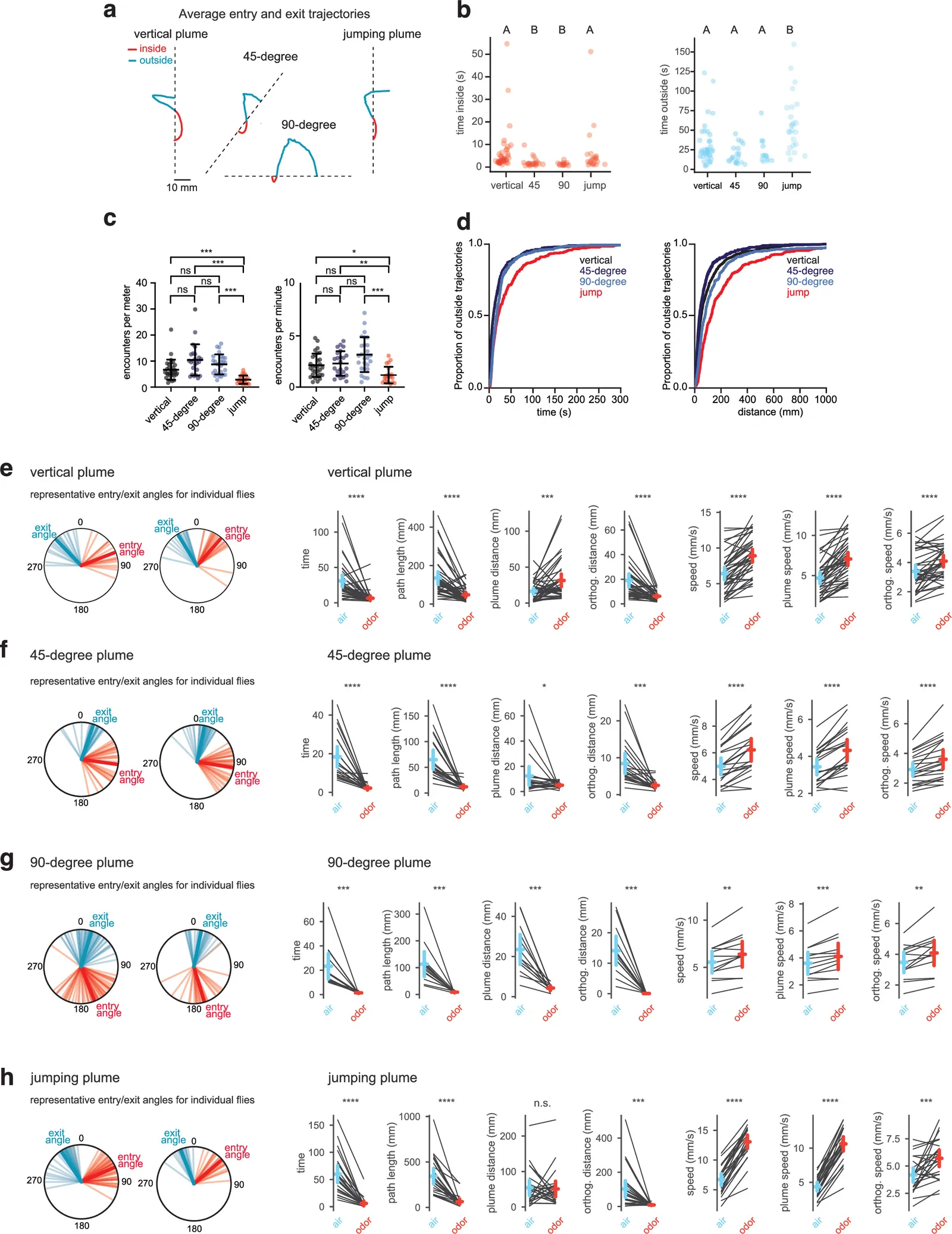
Comparison of edge tracking of plumes with different orientations relative to the wind. (**a**) Comparison of aligned average trajectories for flies tracking indicated plume orientations, n = 20–40 flies, replotted from Fig. 2e–h to highlight the variation in the inside (red) and outside (blue) trajectories of flies depending on the geometry of the plume they are tracking. (**b**) Comparison of time spent inside or outside the plume across different plume geometries, with each dot indicating the average of all trajectories for a single fly. n = 20–40 flies. Letters denote statistically different groups (p < 0.05). (**c**) Comparison of number of encounters per metre of path length (left) or encounters per metre (right) for different plume orientations. Each dot represents a single fly. (**d**) Cumulative distribution of the time and distance of outside trajectories for different plume orientations. (**e**–**h**) Left, entry (red) and exit (blue) angles for two representative individuals tracking the indicated plumes shows that flies use a restricted set of angles as they edge track. Right, comparison of behavioural metrics in clean air outside the plume (blue) and in odour (red) for indicated plumes. (**e**) 0° (with data replotted from Fig. 1); (**f**) 45°; (**g**) 90°; (**h**) jumping plume. Each data point indicates the average for all inside or outside bouts for an individual trajectory. n.s., not significant, *p < 0.05, **p < 0.01, ***p < 0.001, ****p < 0.0001. Details of statistical analyses and sample sizes are given in Supplementary Table 1.

Flies tracked the boundaries of both 45° and 90° tilted plumes using a similar behavioural strategy as was observed for vertical plumes (0°): after entering the odour corridor, they rapidly reoriented to steer upwind, and after losing contact with the plume, they performed directed returns back to the plume’s boundary (Fig. 2e–g and Extended Data Fig. 6a–g). Across plume geometries, flies preferentially exited in the upwind direction, leading them to track tilted plumes almost exclusively along their upwind edge. Nevertheless, both entry and exit angles varied systematically depending on the plume’s orientation. For example, to exit a vertical plume, flies must steer laterally away from the upwind direction. By contrast, flies could exit the 45° and 90° plumes using a large range of upwind angles, yet typically biased their exits in the direction that they were progressing along the plume’s length (Fig. 2e–g and Extended Data Fig. 6a,e–g). Entry angles depended even more strongly on the plume’s orientation, reflecting the fact that animals must walk in a crosswind direction to return to a vertical plume, but downwind to return to a 90° plume. Inside trajectories were shorter in tilted plumes than in vertical plumes (Extended Data Fig. 6b), because flies exited more rapidly when steering upwind within the odour. Outside trajectories were on average the same length despite the fact that flies had to counter their intrinsic upwind drive^6,16,29^ to return to the tilted plume’s boundary (Extended Data Fig. 6b,d). Nevertheless, flies tracked all plume geometries with a similar efficiency (Extended Data Fig. 6c).

Comparing simulated trajectories—built from the experimental distributions of run lengths and turn angles—with the paths of real flies underscored the importance of plume-orientation-dependent biases for effective tracking (Extended Data Fig. 5f–h, left). As observed for a vertical plume, simulated outside trajectories lacking an upwind bias could return to the boundary of a 45° or 90° plume, although much less efficiently than real trajectories. However, incorporating the upwind bias that real flies display outside the plume (Fig. 1b,d) resulted in simulated trajectories that strayed far away from the boundary and often failed to return (Extended Data Fig. 5f–h, right), emphasizing that successful tracking requires countering this upwind drive. Flies thus adapt their outside trajectories depending on the geometry of the plume.

**Extended Data Fig. 7:**
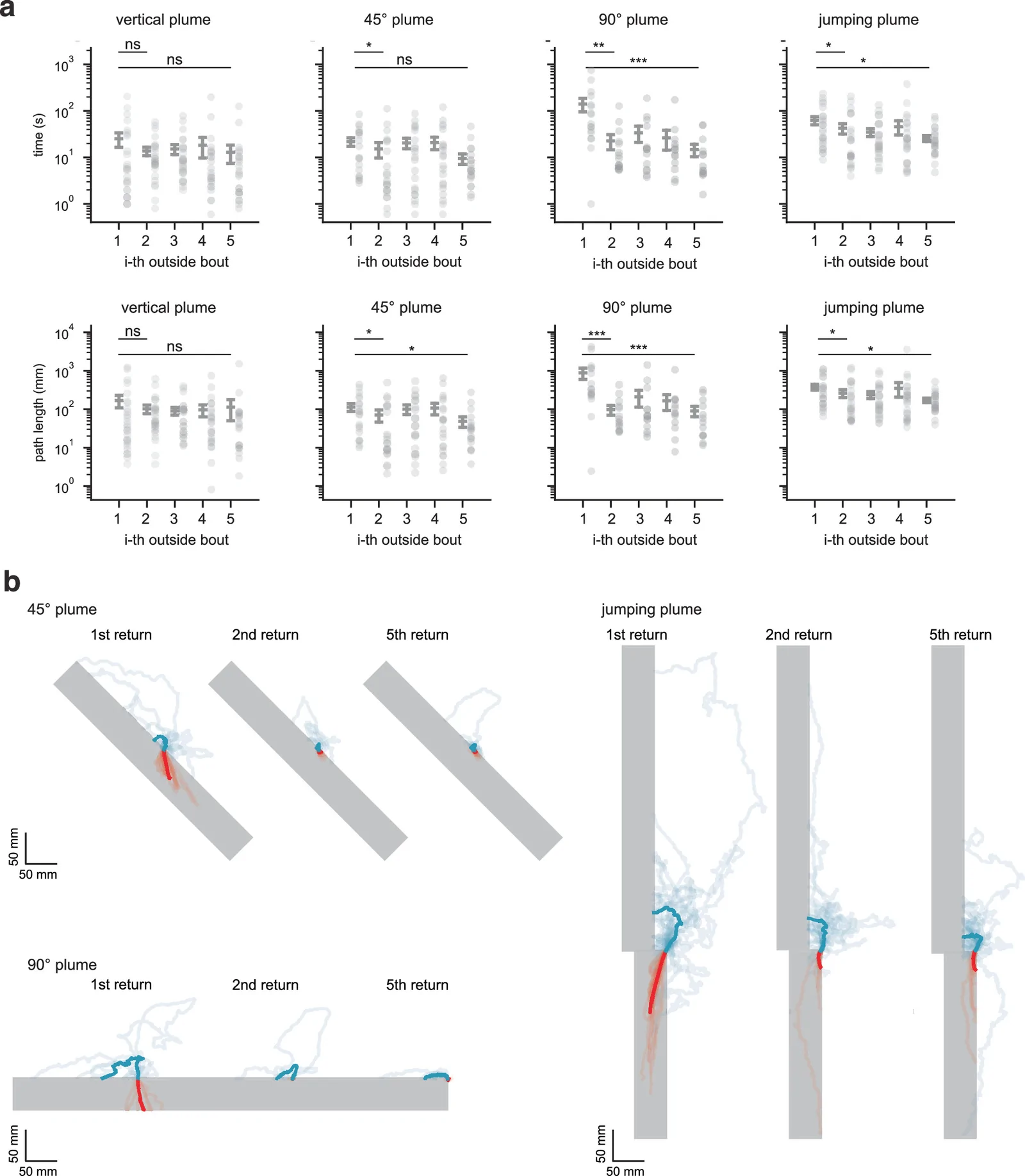
Flies modify their trajectories with experience. (**a**) Plots depicting the change in time (top) or path length (bottom) of sequential outside bouts for flies tracking different plume types (0°, 45°, 90°, and jumping plumes). Each point represents a single bout with the lines representing the mean ± SEM. n = 11−20 flies (**b**) Aligned trajectories showing the first, second, and fifth sequential returns for flies tracking the 45°, 90°, and jumping plume to highlight the increased efficiency of returns with experience. Trajectories for individual flies shown as semi-transparent lines and average across individuals shown as solid lines, with bouts in the plume (red) and outside the plume (blue), n = 7–24. n.s., not significant, *p < 0.05, **p < 0.01, ***p < 0.001, Details of statistical analyses and sample sizes are given in Supplementary Table 1.

Across plumes, return trajectories became more efficient over the first few successive excursions, suggesting that prior experience may contribute to a directional memory to guide a fly’s returns (Extended Data Fig. 7). This effect was not apparent for vertical plumes, where re-encounters can often occur by chance. To directly assess whether flies rely on a memory of the plume’s direction, we allowed flies to track a 45° plume for 10 min before removing it while they were on an outside excursion (Fig. 2i). Before the plume vanished, most flies exited and re-entered it more than three times, which we reasoned was sufficient for them to form a directional memory given their rapid experience-dependent improvement. Consistent with this, after the plume’s disappearance, flies headed in the same direction that they had previously used to return to the plume and maintained this bias, even as it carried them hundreds of millimetres away from the location of their last plume encounter. These directional biases were distinct from the upwind bias that flies generally showed before encountering the plume (Fig. 2i,j).

One implication of storing a directional bias to the edge is that it should allow flies to track dynamic plumes that shift over time. Indeed, flies could readily track a ‘jumping’ plume, in which each time a fly exited the plume’s edge, we jumped the plume 20 mm in the opposite direction (Fig. 2d,h and Supplementary Video 3). Successful tracking required flies to both bias their returns towards the plume’s boundary and walk even further to re-encounter it, resulting in significantly longer trajectories than for stationary plumes (Extended Data Fig. 6b,d,h). Notably, unlike the local search elicited by the loss of a discrete reward, such as a sugar droplet^30^, flies did not dwell around the locations of their last plume encounters, suggesting that edge tracking relies on a directional but not a positional memory.

## Compass signals support edge tracking

Our behavioural analyses suggest that flies rely on a directional memory of the plume’s boundary to bias their outside trajectories and direct their returns. We reasoned that the central complex—a conserved spatial navigation centre in insects^9^—provides an ideal neural substrate for encoding and storing this directional memory. A core spatial signal encoded in the *Drosophila* central complex is a fly’s heading direction, which is represented as a single bolus of activity that rotates around topographically organized EPG neurons in the ellipsoid body^31^. This neural compass can be anchored to stable sensory landmarks, such as the sun^32,33^ or a wind source^34^, to allow animals to maintain an accurate representation of their heading in the world.

**Fig. 3:**
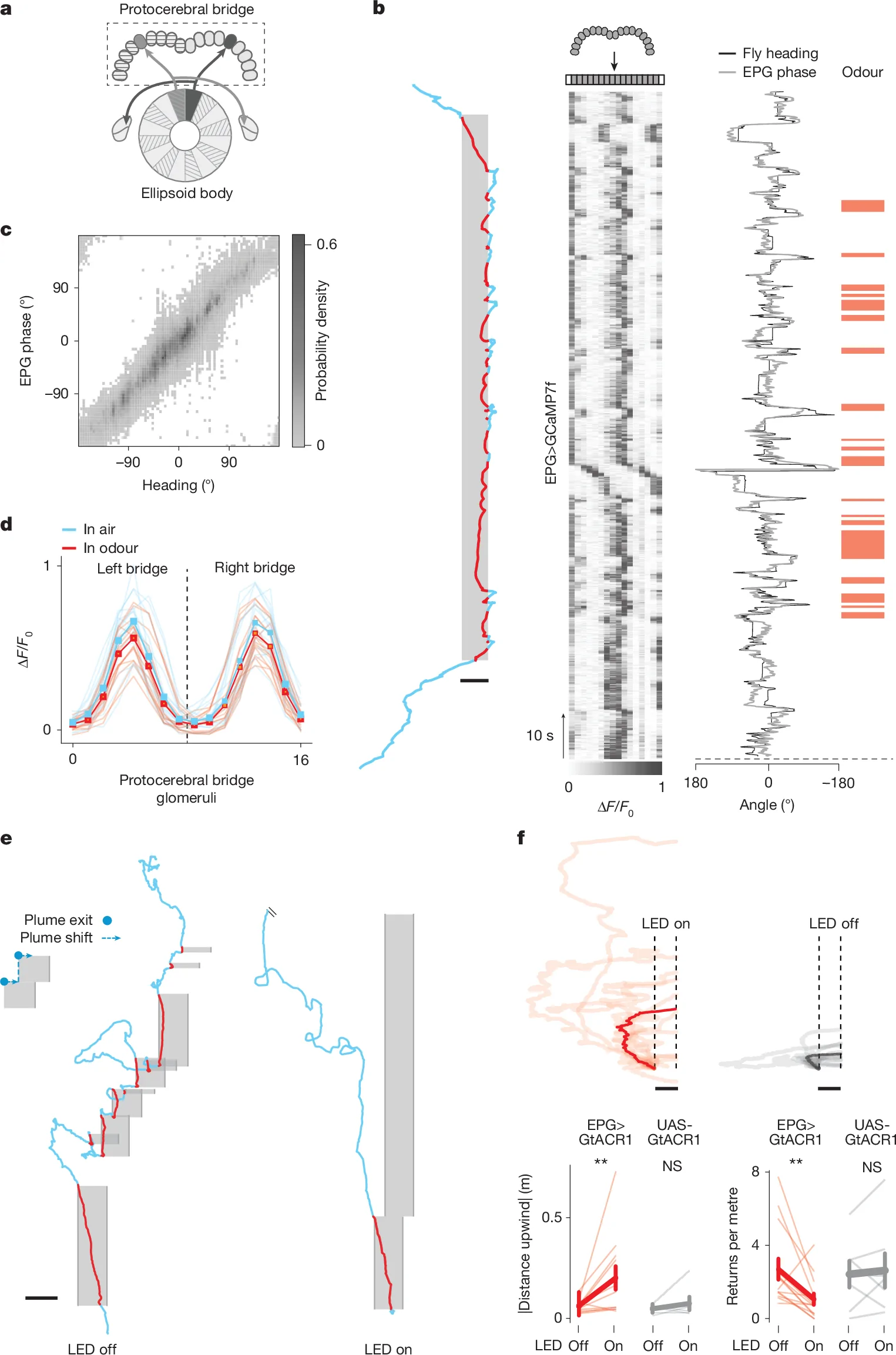
EPG neurons represent a fly’s heading and are required for edge tracking. **a**, Schematic of the central complex neuropils innervated by EPG neurons. Adjacent EPG neurons in the ellipsoid body project to glomeruli in the left and right protocerebral bridge. **b**, Representative recording of EPG activity as a fly tracks a 10-mm plume. Left, trajectory with inside bouts (red) and outside bouts (blue). Middle, heat map of EPG activity (Δ*F*/*F*_0_) across the 16 protocerebral bridge glomeruli as indicated by the schematic at the top. Right, alignment of estimated EPG phase (grey) with the fly’s heading (black) during the same trial; the timing of odour bouts is depicted in red at the far right. **c**, Heat map representing the correspondence between a fly’s heading and estimated EPG phase during edge tracking. *n* = 16 trials from 9 flies. **d**, Amplitude of phase-aligned activity bumps in the left and right protocerebral bridge during edge tracking, in odour (red) and in air (blue). *n* = 16 trials from 9 flies. **e**, Representative trajectory of an EPG>GtACR1 fly tracking a jumping plume in the absence (left) and presence (right) of optogenetic inhibition (LED on throughout the trial). **f**, Top, comparison of average outside trajectories for EPG>GtACR1 flies with EPG neurons silenced (LED on, left) or active (LED off, right). Bottom, distance travelled upwind between plume re-encounters (left) and number of successful return trajectories (right), shown for EPG>GtACR1 and UAS-GtACR1 control flies, with and without optogenetic inhibition. Thin lines, individual flies; thick lines, mean ± s.e.m. EPG>GtACR1 (*n* = 12 flies); UAS-GtACR1 (*n* = 6 flies). NS, not significant; **P < 0.01. Statistical details and sample sizes are in Supplementary Table 1. Scale bars, 10 mm (**b**); 50 mm (**e**); 20 mm (**f**).

To examine the activity of EPG neurons during edge tracking, we performed two-photon functional calcium imaging of their axon terminals within the protocerebral bridge, a linear neuropil that receives two duplicated copies of a fly’s heading direction (Fig. 3a). During edge tracking, the EPG phase remained faithfully aligned to the fly’s heading direction (Fig. 3b,c), underscoring their ability to use a wind source as a directional landmark^34^. The amplitude and width of the boluses of EPG activity were comparable throughout the course of an edge-tracking trial, indicating that this heading signal remains yoked to the wind and unchanged by the presence of odour (Fig. 3d).

An animal’s current heading is thought to be integral to *Drosophila* navigation because this signal acts as a reference for other spatial variables, such as the fly’s travelling direction^35,36^, and is compared with a stored goal direction to guide steering behaviour^6,37–40^. To assess whether edge tracking relies on EPG activity, we silenced these neurons using the light-gated anion channel GtACR1 while flies tracked a jumping plume, in which successful navigation requires them to maintain a persistent bias to return to the plume’s shifted edge (Fig. 2d,h). Flies in which EPG neurons were silenced no longer made directed returns to the plume’s boundary, and instead frequently wandered away in the upwind direction, leading to fewer and less-efficient returns (Fig. 3e,f). LED illumination did not affect tracking in control flies, supporting the specificity of this manipulation (Fig. 3f). EPG neuron activity—and the stable heading representation it conveys—thus appears to be necessary for effective edge tracking, suggesting that navigating structured chemical landscapes depends on core elements of the central complex circuitry.

## A behavioural model of edge tracking

We next investigated which spatial cues flies might use to form or update a memory of the plume’s direction during edge tracking. Although flies could enter or exit an odour corridor over a 180° range, they consistently adopted a narrow range of angles that depended on the plume’s geometry (Fig. 2e–h and Extended Data Fig. 6e–h). We therefore hypothesized that each time a fly crossed the plume’s boundary, it stores its current heading relative to the wind direction as an angular memory that could guide subsequent trajectories.

**Fig. 4:**
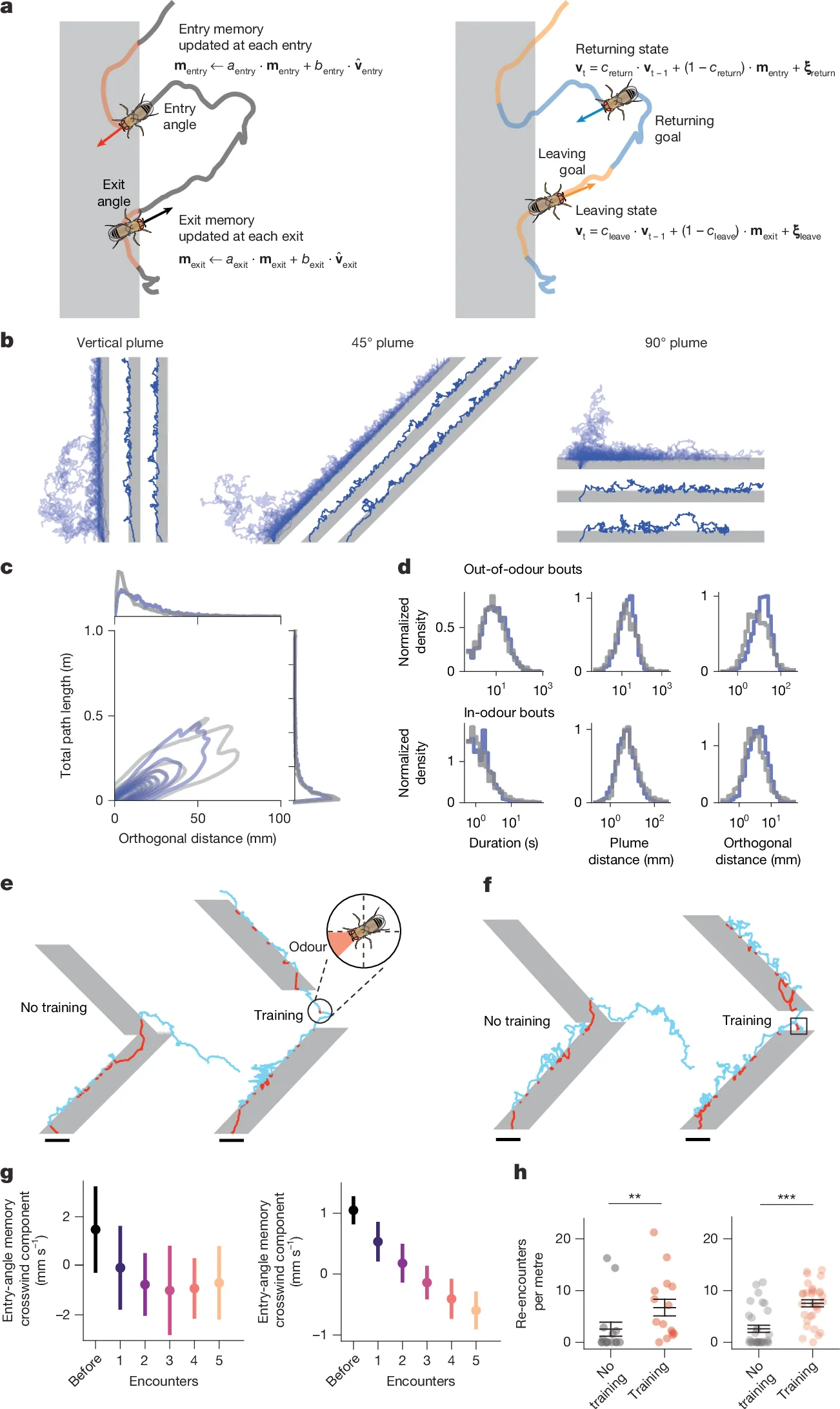
A behavioural model of edge tracking suggests that flies update entry-angle memories rapidly with experience. **a**, Components of the switching state-space model mapped onto a schematized trajectory of a fly tracking a vertical plume (grey). In the leaving state (orange), the fly relies on its exit-angle memory (**m**_exit_) updated at each plume exit (black arrow). In the returning state (blue), it relies on its entry-angle memory (**m**_entry_) updated at each plume entry (red arrow). **b**, Representative simulated trajectories for plumes of different orientations relative to the wind. For each, two individual trajectories (dark lines) are shown alongside an overlay of 25 trajectories (semi-transparent). **c**, Comparable efficiency of simulated and real fly trajectories revealed by plotting total path length versus perpendicular distance from the plume boundary for all outside bouts across 72 flies (grey) and 75 simulations (blue) tracking 0°, 45° and 90° plumes. The first outside bout was excluded for both real and simulated flies, given the lack of an entry-angle memory before the first return. **d**, Histograms comparing metrics across 72 flies (grey) and 75 simulations (blue) for 0°, 45° and 90° plumes. *Y* axis values show normalized counts such that area under curve is 1. **e**, Left, trajectory of a naive fly given two 45° plume segments, showing that, without training, flies continue in the direction needed to re-enter the first segment. Right, a single operant training session (Methods), in which odour was delivered when the fly spontaneously walked between –90° and –135°, enables the fly to track an oppositely oriented 45° plume. **f**, Model simulations of the same experiment as in **e**. Scale bars, 50 mm (**e**,**f**). **g**, Crosswind component of entry-angle memory as a function of odour encounters during operant training, inferred from real (left) or simulated (right) fly trajectories. Memory is a velocity vector indicating the goal, so its units are mm s^−1^. Mean ± s.d.; *n* = 29 simulations; *n* = 10 flies. **h**, A single operant training trial enhances the ability to track a 45° plume segment oriented in the opposite direction. Number of plume re-encounters per metre of the second plume segment for both real (left) and model (right) flies. **P < 0.01, ***P < 0.001. Statistical details and sample sizes are in Supplementary Table 1. The schematics in **a**,**e** are adapted with permission from ref. ^15^ (Cell Press).

**Extended Data Fig. 8:**
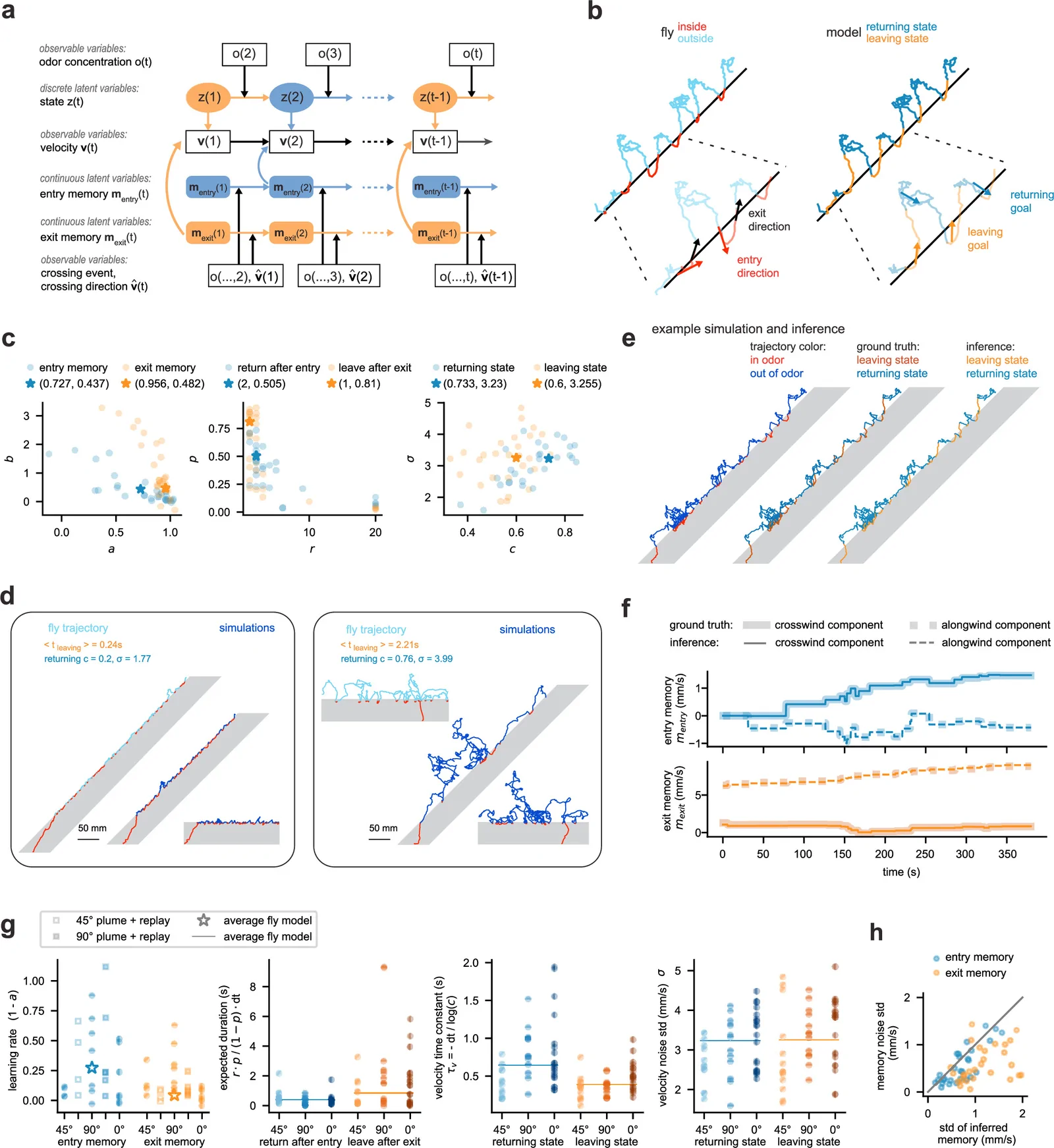
A switching state-space model for edge tracking. (**a**) Diagram of the switching state-space model used to infer fly navigation states (discrete latent variables) and memories (continuous latent variables). The model also infers transition rates between states and parameters of the memory and velocity update rules. See Methods for details. (**b**) Representative example of a real fly trajectory with navigation states and memories inferred. Left: trajectory is coloured according to when the fly is inside the plume (red) or outside the plume (light blue), with arrows indicating entry direction (red) and exit direction (black) as flies cross the plume’s boundary. Right: The same fly trajectory coloured according to leaving state (orange) and returning state (blue) with arrows indicating inferred leaving goal (orange) and returning goal (blue). Note that leaving and returning states are not equivalent to times when the fly is inside or outside the odour plume. (**c**) Scatter plots showing the distribution of parameters for the 28 trajectories (dots) from flies that made at least 30 returns which were used to train the model, and parameters for the average fly model (stars), see Methods for details. Average fly model parameters: w∥ = 6.299 mm/s; w⊥ = 1.695 mm/s; entry memory (a = 0.727, b = 0.437 mm/s) and exit memory (a = 0.956, b = 0.482 mm/s); return after entry (r = 2, p = 0.505) and leave after exit (r = 1, p = 0.81); returning state (c = 0.733, *σ* = 3.23 mm/s) and leaving state (c = 0.6, *σ* = 3.255 mm/s). (**d**) Individual flies display heterogeneity in how closely they track the edge of the plume, as reflected in distinct model parameters corresponding to different state transitions and velocity updates. Each box shows the representative trajectory for an individual fly (left, cyan) used to fit model parameters and two simulations (right, blue) for 45° and 90° plumes. All bouts inside the odour are shown in red. (**e**) Example inference of navigational states (right) compared with ground truth (middle) for a simulated trajectory. (**f**) Example inference of memories (thin lines) compared with ground truth (thick lines) for the simulated trajectory in (e). (**g**) Parameter distributions across different experiments. The first panel shows the learning rate of 28 training trajectories (trajectories including replay experiment are shown by square markers). The second to last panels show state and velocity parameters for 46 edge tracking trajectories with at least 20 returns. (**h**) Joint distribution of the memory noise parameter and standard deviation of inferred memories for 28 training trajectories. Inferred memories here are the mean of variational distributions fitted to individual trajectories. Standard deviation of inferred memories is calculated as the geometric mean of crosswind component standard deviation and alongwind component standard deviation.

To formalize how flies might generate and update these angular memories, we developed a switching state-space model^41,42^, in which simulated flies alternate between ‘leaving’ and ‘returning’ states as they progress along a plume’s boundary (Fig. 4a). These states do not correspond directly to epochs inside or outside the plume, although the transition rates between them depend on the presence of odour (Extended Data Fig. 8a,b). The model generates a velocity vector through an auto-regressive process, with a correlation timescale and noise level determined by the current state. Notably, the velocity vector is biased by the memory of previous exit angles (for the leaving state) or previous entry angles (for the returning state), and these are updated each time the simulated fly crosses the odour boundary. The updated memory is a linear combination of its past entry or exit memory and current heading. We used variational inference to extract the latent leaving and returning states, and latent goal directions, and to learn the parameters that determine the velocity and memory updates from the trajectories of individual flies tracking different plume geometries (Extended Data Fig. 8g and ‘Model’ in Methods). Model parameters varied across individuals, matching the heterogeneity that flies display in their outside trajectories, with some individuals adhering closely to the plume’s boundary and others taking longer and more-circuitous excursions before returning (Extended Data Fig. 8c–f). From these fits, we computed average model parameters to simulate fly trajectories and reveal how leaving and returning goal directions emerge from repeated plume encounters (Fig. 4b).

**Extended Data Fig. 9:**
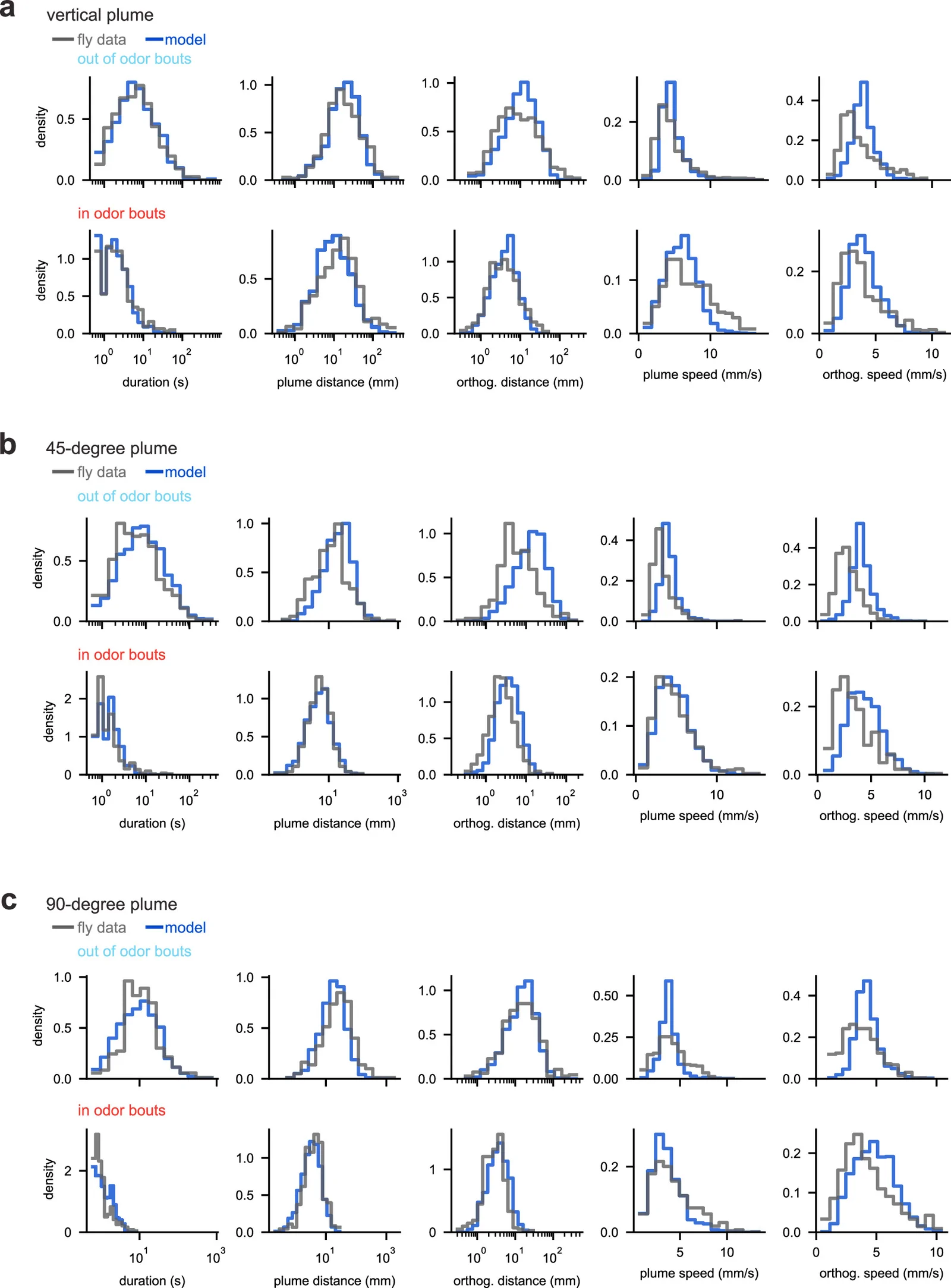
Model captures the average statistics of edge tracking across different plume geometries. (**a**–**c**) Histograms comparing metrics of real and simulated fly trajectories tracking indicated plume orientations: (**a**) vertical (0°) (28 flies, 25 simulations), (**b**) 45° (24 flies, 25 simulations), and (**c**) 90° (20 flies, 25 simulations). These represent the same set of trajectories as in Fig. 4b but now divided by plume geometry. Y-axis values show normalized counts such that area under curve is 1. See Supplementary Table 2 for statistical analyses.

**Extended Data Fig. 10:**
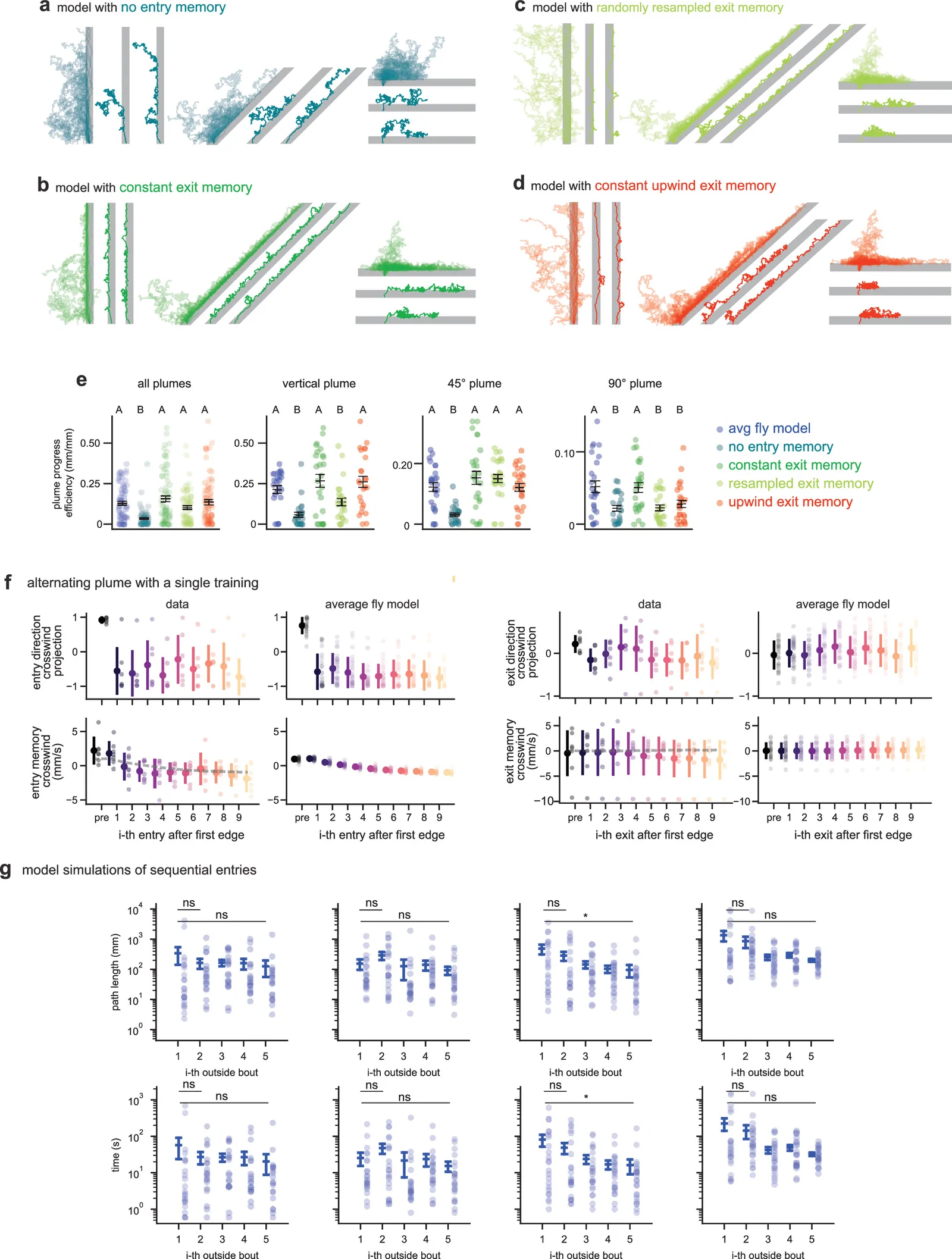
The role of entry- and exit-angle memory in the model. (**a**) Simulated trajectories tracking vertical, 45° and 90° plumes using a model where entry memory is maintained as a null vector (no entry memory). For each plume, two individual representative trajectories are shown (thick lines) along with an overlay of 25 trajectories. (**b**) Same as (**a**) but for simulated trajectories using a model that has a constant exit memory in the upwind direction with a random crosswind component (constant exit memory). (**c**) Same as (**a**) but for simulated trajectories using a model where the exit memory is resampled from the initialization distribution at each exit (resampled exit memory). (**d**) Same as (**a**) but for simulated trajectories using a model with a constant upwind exit memory (upwind exit memory). Note the same seeds were used for all models. (**e**) Comparison of plume progress efficiency (distance between first and last exit divided by total path length) for indicated model variants, mean ± SEM shown in black. Letters denote statistically different groups (p < 0.05). (**f**) Changes of the crosswind components of entry memory, entry direction, exit memory, and exit direction for alternating plume experiment with a single operant training period (see Fig. 4e,f). Results shown for trajectories with more than 9 entries after tracking the first edge. Error bars show standard deviation. Top: Crosswind component of entry (left) and exit (right) angle direction for 7 real flies and 28 simulated trajectories. Bottom: Same as top but for inferred entry (left) and exit (right) angle memory. Dashed grey line represents average model plotted for comparison. (**g**) Plots depicting the change in path length (top) or time (bottom) of sequential outside bouts for simulated flies tracking different plume types (0°, 45°, 90°, and jumping plumes). Each point represents a single bout with the lines representing the average value ± standard error for n = 19–23 simulations. *p < 0.05, ns, not significant p > 0.05. Details of statistical analyses and sample sizes are given in Supplementary Table 1.

The model requires that we initialize the memory vectors at the beginning of a simulation. Without information about the prior experience of a given fly, we set the initial exit memory to be upwind with a narrow randomly chosen crosswind component (Methods), consistent with the upwind bias that flies exhibit as they leave the plume (Fig. 2e–h and Extended Data Fig. 6e–h). The memory of the entry angle was initialized as a null vector—aligned with the assumption that flies have no prior information about which direction to return before encountering a plume, but that an angular goal is established and reinforced with repeated plume encounters. Simulations based on this model reproduced the experimental statistics of edge tracking across different plume geometries (Fig. 4b–d and Extended Data Fig. 9). Removing the entry-angle memory from the model significantly impaired edge tracking across all plume orientations, underscoring that this angular memory is essential for flies to advance efficiently along the plume’s boundary (Extended Data Fig. 10a,e). By contrast, tracking did not require continuous updating of the exit-angle memory, although simulations in which the exit memory was purely upwind or resampled at each exit showed impaired performance in some plume geometries (Extended Data Fig. 10c–e). Perturbations of the model thus reveal that entry- and exit-angle memories differentially contribute to edge tracking: entry angles dynamically instruct an angular goal to guide returns to the plume’s boundary, whereas exit angles define a stable steering bias.

## Edge tracking of dynamic plumes

**Extended Data Fig. 11:**
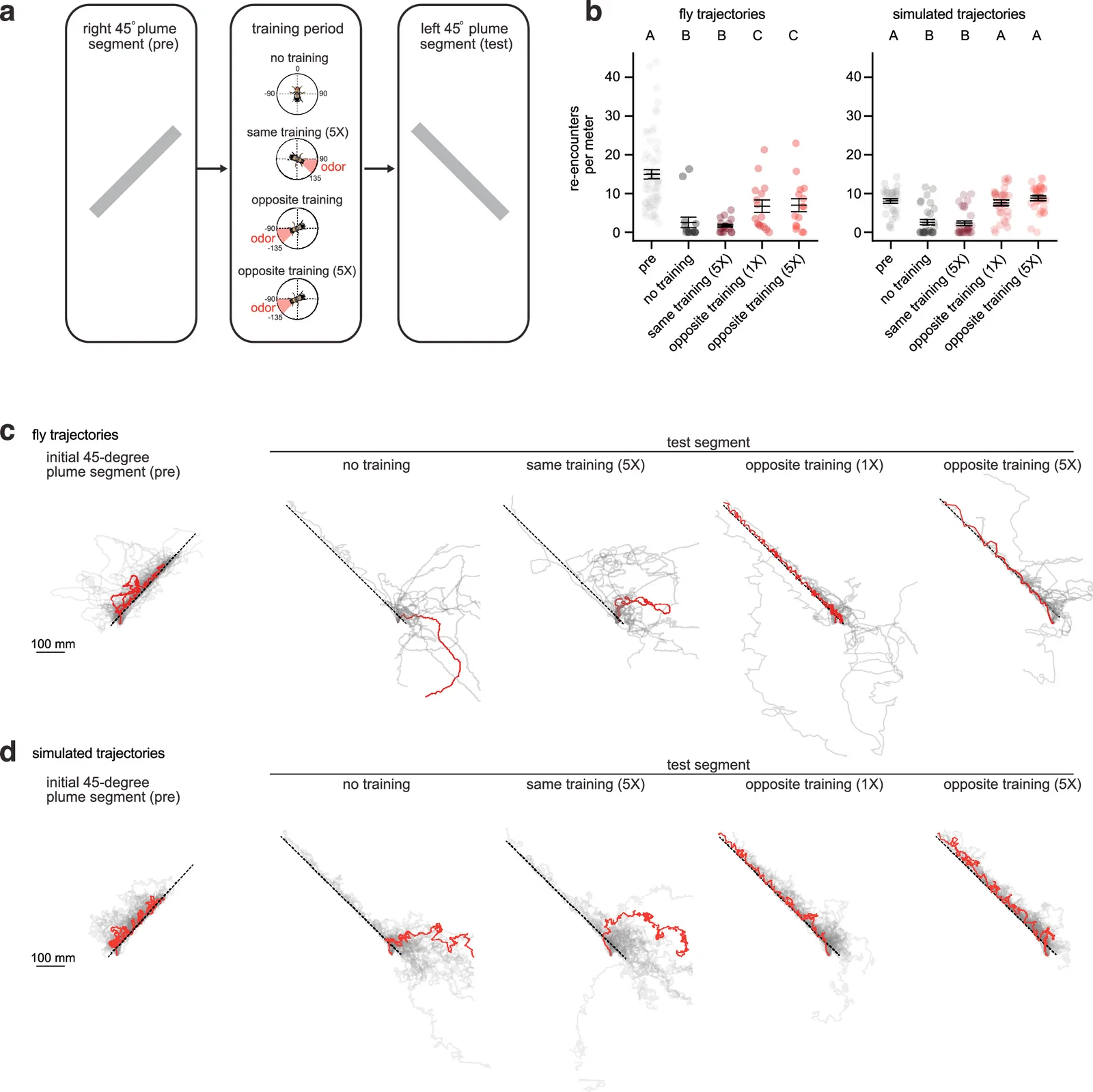
Flies can be operantly trained to track plumes of different orientations. (**a**) Schematic of experimental protocol used to operantly train flies to track plumes of different orientations. After a 5-minute baseline period during which flies walked in clean air, they were given a 45° plume to navigate. Flies that edge tracked a plume for at least 250 mm were then subjected to one of four training conditions: No training, in which flies were immediately tested with a −45° plume in the opposite direction without reinforcement; Same training (5X), in which odour delivery was triggered when flies were heading in the same direction that would lead them back to the initial plume segment (e.g. the 90° to 135° sector), and ceased upon entering a 90° upwind exit zone (−45° to 45°) (training was repeated five times); Opposite training (1X), in which odour delivery was triggered when flies travelled in the direction (e.g. −90° to −135° sector) required to enter the second plume segment. Note that to enter the second plume segment flies must travel in the same direction as used for operant conditioning (−90° to −135°) and so effectively have a second operant training epoch; Opposite training (5X), consisting of five bouts of odour reinforcement triggered by entering the −90° to −135° sector required to enter the second plume segment. Plume orientations (e.g. whether the initial plume segment was oriented 45° or −45°) were interleaved across trials for consistency and the type of trial was defined in advance. (**b**) Comparison of plume re-encounters per metre across the five conditions for real flies (left) and simulated trajectories (right). n = 17 flies for same training, n = 15 flies for no training and opposite training. n = 31 simulations for each condition. Average ± SEM shown. (**c**) Overlaid trajectories for flies tracking the indicated plume segments: initial plume, no training, same training (5X), opposite training (1X), and opposite training (5X). Individual trajectories shown as thin black line with a single representative trajectory shown in red and plume’s edge is depicted as dashed line. (**d**) Same as in (**c**) except for simulated trajectories. Letters denote statistically different groups (p < 0.05). Details of statistical analyses and sample sizes are given in Supplementary Table 1.

To gain experimental evidence that the entry-angle memory can be rapidly updated, we examined how flies adapt their trajectories when the plume geometry changes abruptly. We provided flies with a plume consisting of two 45° segments oriented in opposite directions relative to the wind (Fig. 4e and Extended Data Fig. 11a). Successfully tracking both segments requires distinct entry-angle memories around 90° apart. After tracking the first (+45°) plume segment, most flies showed a persistent directional bias in the downwind direction, analogous to the bias observed when the plume disappeared (Fig. 2i). Consequently, flies rarely re-encountered or tracked the second (−45°) segment (Fig. 4e and Extended Data Fig. 11b,c), consistent with the notion that they require additional experience to update their stored goal direction. We therefore introduced an operant ‘training’ session after flies tracked the first +45° plume segment, by delivering odour when they spontaneously walked in the direction required to enter the second plume’s boundary (for example, −90° to −135°; Fig. 4e and Extended Data Fig. 11a). A single operant session was sufficient to significantly improve tracking of the second plume segment (Fig. 4e,g,h), with no further enhancement from additional training. By contrast, training in the direction required to enter the first plume segment (+90° to +135°) had no effect (Extended Data Fig. 11b,c). Model simulations indicate that the entry-angle memory can shift after only a few successive odour encounters, although memory updating was slower than inferred from real behavioural trajectories (Fig. 4f,g and Extended Data Fig. 10f), implying that the model’s fixed learning rate underestimates flies’ true memory dynamics.

**Extended Data Fig. 12:**
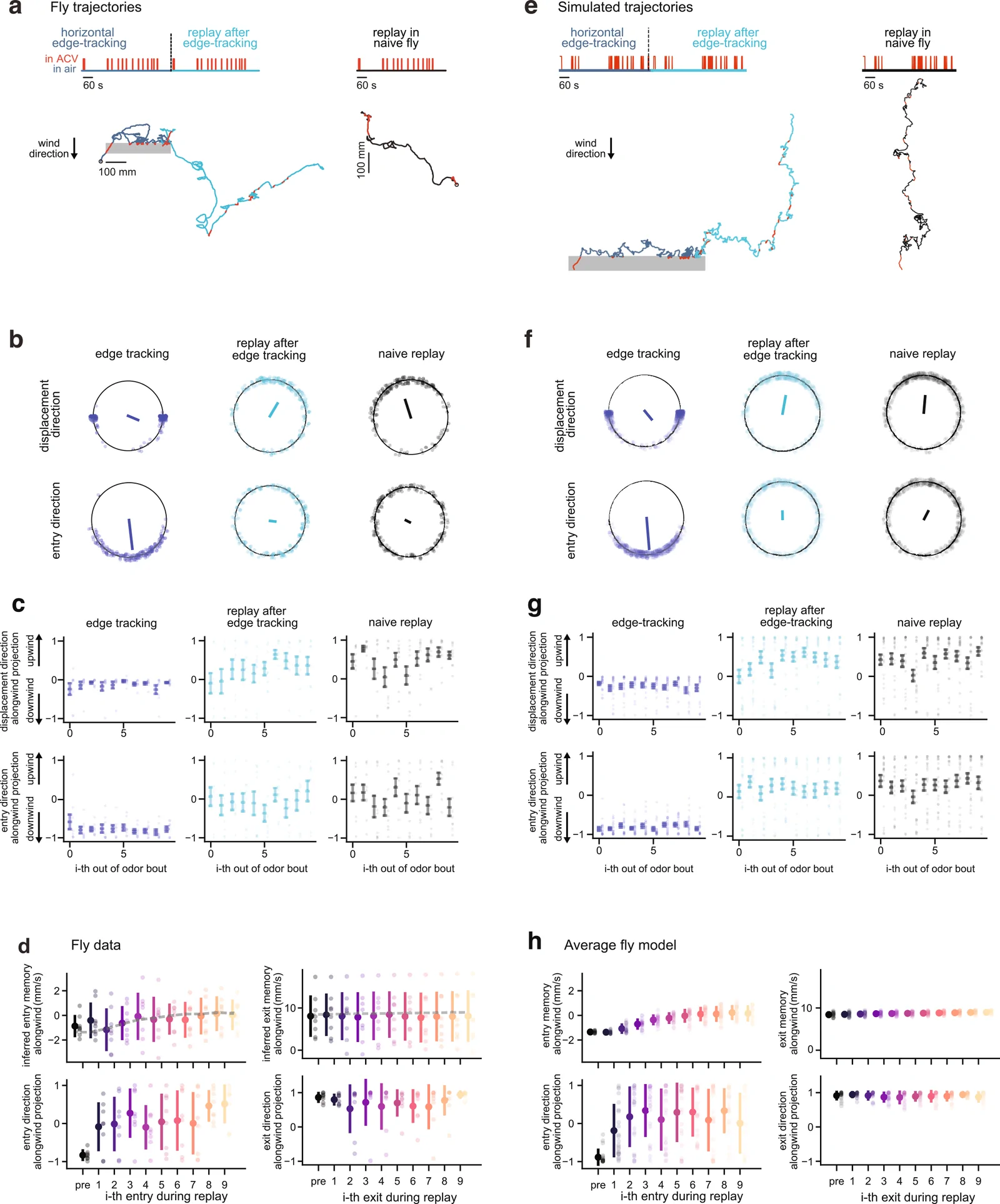
Fly trajectories depend on the spatial structure of their olfactory experience. (**a**) Left: Representative ‘replay’ experiment in which a fly was allowed to edge track along a 90° plume for 10 min, after which the same temporal sequence of odours was presented back to the same fly. Schematic on top depicts the sequence of odour encounters during edge tracking that was replayed to the fly during the replay epoch. Representative trajectory during edge tracking shown as red in odour and dark blue when outside the odour, while trajectory during replay epoch is red inside the odour and cyan when outside the odour. Right: Trajectory of a naive fly when presented with the same odour sequence. (**b**) Polar plots showing the net displacement direction when flies are outside the odour (top) and entry direction when they encounter the odour (bottom) during edge tracking (left, dark blue), replay to the same fly (middle, cyan), or replay to naive flies (right, black). Each point represents a single bout for an individual fly, with the average vector for all flies shown as a line. n = 8-9 flies. Only trajectories with at least 10 effective entries during replay are included. (**c**) The displacement direction and entry direction of flies over sequential outside bouts of edge tracking trials (left, dark blue), replay of the odour sequence to the same flies (middle, cyan), and replay of the odour sequence to naive flies (right, black). n = 8 flies. The alongwind projection is shown, with 1 denoting straight upwind and −1 denoting straight downwind, to emphasize the change in the direction of outside bouts depending on the conditions in which the fly encounters the odour. Error bars show S.E.M. (**d**) Changes of the alongwind component of the entry-angle memory, entry direction, exit memory and exit direction during olfactory replay after tracking a 90° plume. Results shown for trajectories with more than 9 entries during replay. Left: Entry and exit direction and inferred entry- and exit-angle memory for 8 real flies. Inferred memories are the mean of variational distributions fitted to individual trajectories. Dashed grey line indicates average from corresponding model trajectories plotted for comparison. Error bars show standard deviation. (**e**–**h**) Same as (**a**–**d**) but shown for 21 simulated trajectories, with at least 10 effective entries during replay. For simulations, entry- and exit-angle memory represent ground truth memory.

One consequence of such rapid updating is that the entry-angle memory could be quickly overwritten when odour is encountered at irregular or unpredictable angles. To investigate this, we allowed flies to track a 90° plume and then immediately replayed the same temporal sequence of odour encounters back to them but now uncoupled from their movements (Extended Data Fig. 12a), effectively scrambling the entry and exit angles that reinforce a coherent angular memory. During these replay epochs, flies initially maintained the downwind bias required to return to the odour corridor, but this bias dissipated quickly with subsequent encounters (Extended Data Fig. 12b–d), converging towards the upwind heading exhibited by naive flies^3,18^. Our model replicated this effect (Extended Data Fig. 12e–h), reinforcing the idea that the angular memory supporting edge tracking can be rapidly updated, but also overwritten when odour encounters convey inconsistent directional information.

**Fig. 5:**
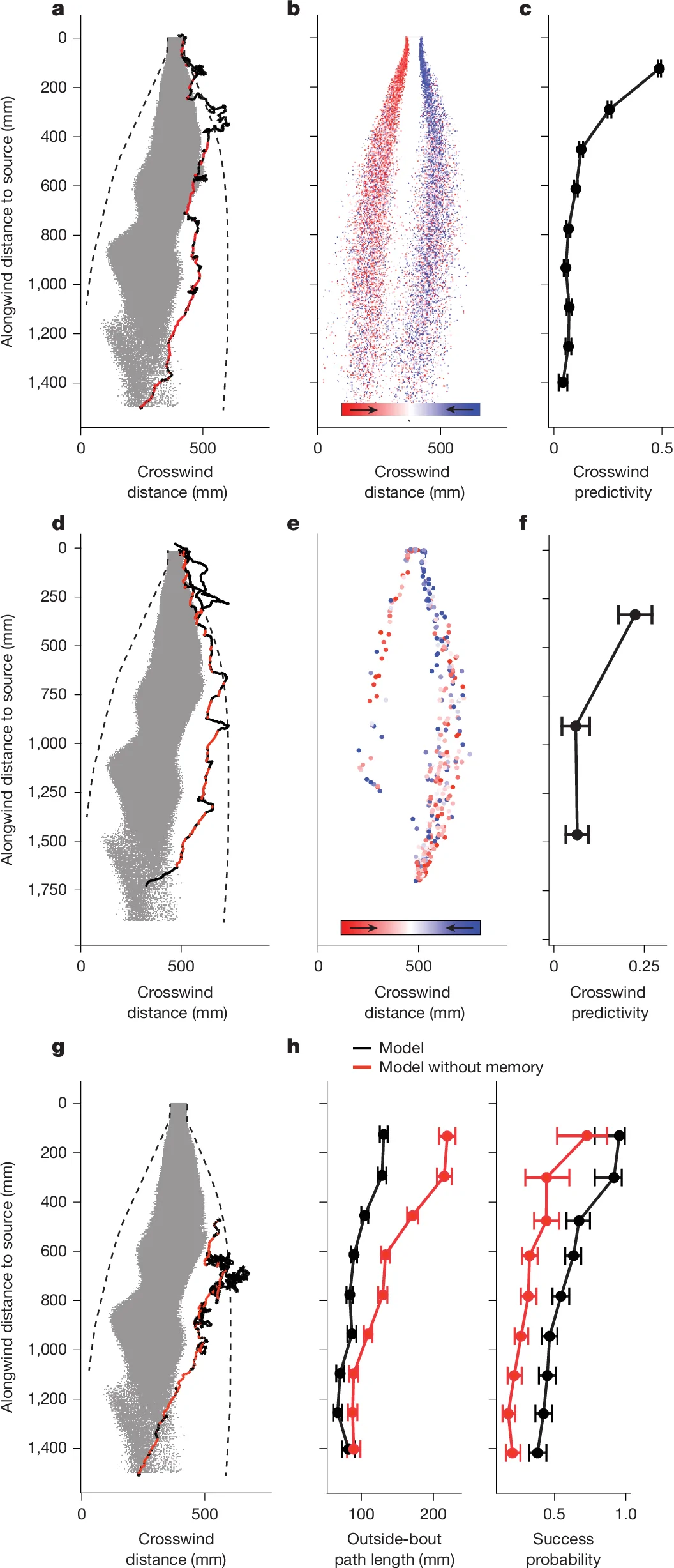
Edge tracking in naturalistic plumes. **a**, Representative simulated trajectory overlaid on a snapshot of a scaled naturalistic plume^17^. Red, epochs in odour; black, epochs out of odour. Dashed line indicates the average plume envelope over a full simulation. **b**, Spatial map of the crosswind component of entry-velocity unit vectors, with each dot representing the average for that pixel location. Red = 1; white = 0; blue = −1. **c**, Crosswind predictivity of entry memory for simulated plumes as a function of alongwind distance to the source, calculated as the product of the crosswind components of the entry-velocity and entry-memory unit vectors (Methods). Mean ± s.e.m.; *n* = 1,850 simulations. **d**, Representative trajectory of a real fly tracking the same naturalistic plume. Colouring and dashed envelope as in **a**. **e**, Spatial map of the crosswind entry directions for real fly trajectories. Each dot represents a single entry. *n* = 12 flies. **f**, Crosswind predictivity of entry memory inferred for real flies (Methods). Entries were grouped into three evenly spaced bins spanning the most downwind to the most upwind entries, excluding entries within 50 mm of the source. Points indicate the mean alongwind position of entries within each bin. 10 out of 12 flies reached within 50 mm of the source. Mean ± s.e.m.; *n* = 12 flies. **g**, Representative simulated trajectory without entry memory; colouring and dashed envelope as in **a**. **h**, Comparison of simulated trajectories with (black) and without (red) entry memory. Left, path length of outside bouts as a function of alongwind distance. Mean ± s.e.m., *n* = 1,850 simulations. Right, probability that a simulated trajectory succeeds in reaching within 50 mm of the source as a function of the alongwind location of the first plume entry (Methods). Only trajectories with one or more effective entries are shown. Error bars, 95% Wilson score confidence intervals. See Supplementary Table 1 for full statistics and sample sizes.

**Extended Data Fig. 13:**
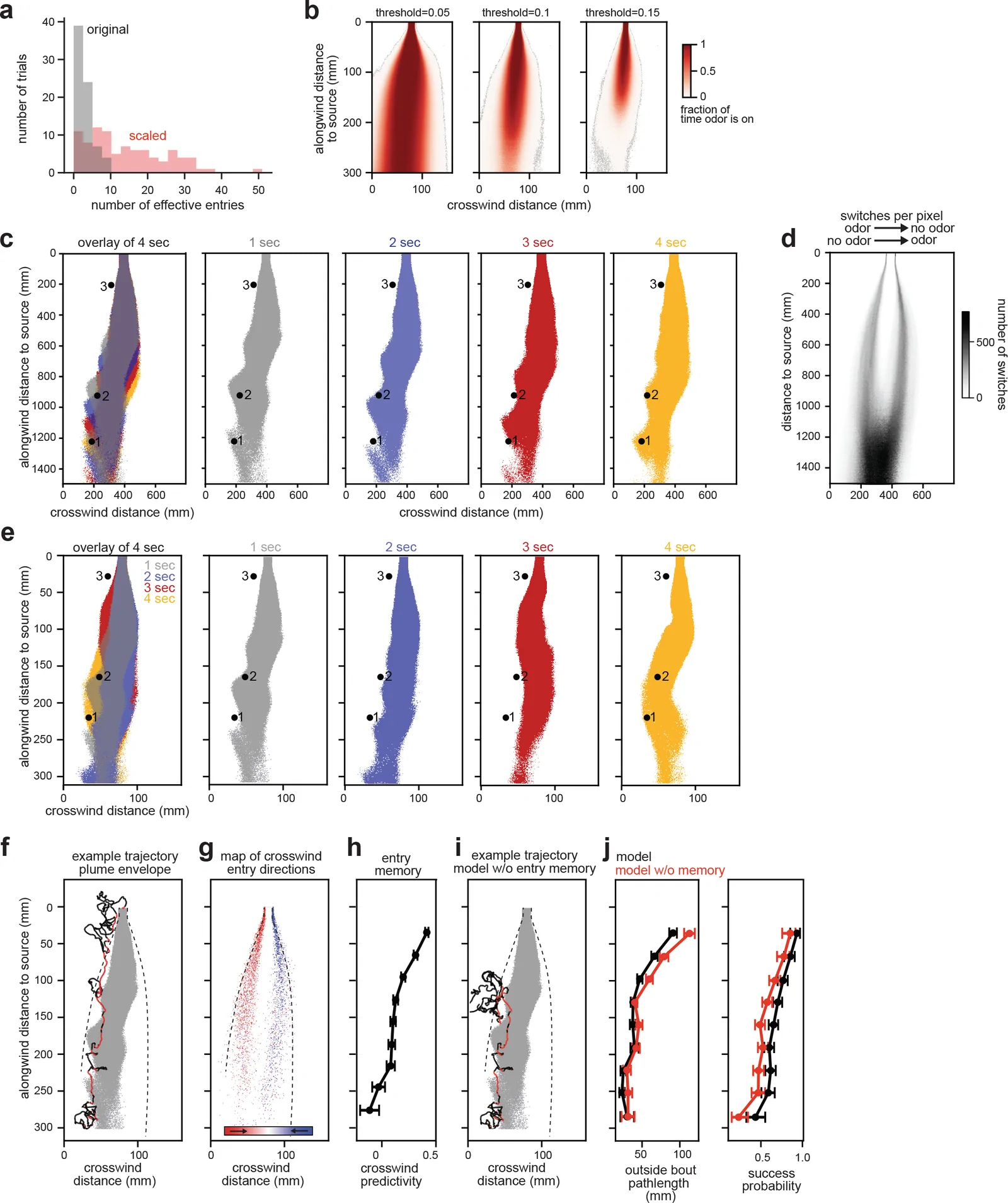
Models of edge tracking in naturalistic plumes. (**a**) Comparison of the number of effective plume trajectories for the edge tracking model applied to a previously recorded surface plume^17^ in its original dimensions (unscaled, black) or isometrically scaled 5-fold (red). Note that the edge tracking model only writes in an entry memory for entry events ≧ 1 sec (see Methods) which avoids spurious events arising from transient plume encounters. (**b**) Spatial maps showing the fraction of time odour exceeds a specific threshold at each pixel across the plume for different thresholds. In simulations, a threshold of 0.1 was used to ensure sufficient odour encounters for navigation while maintaining the turbulent properties characteristic of the downwind region of the plume. (**c**) Four sequential 1 s snapshots of scaled naturalistic plume in different colours (overlaid, left or as individual snapshots (right) to highlight differences in the dynamics of the plume along its length. Note that in more turbulent regions far downwind (points 1 and 2), a simulated fly could exit and successfully re-enter, even if it remains stationary. Closer to the source (point 3), where the plume is more ribbon-like, once a simulated fly exits the plume it cannot re-enter without actively walking into it. (**d**) Spatial distribution of switches in odour per pixel across the plume video. (**e**) Same as (**c**) but for unscaled plume. (**f**) A representative simulated trajectory overlaid onto a single plume snapshot, with red indicating epochs in odour and black- out of odour. The average envelope of the plume is shown as a dashed line. Note that while the simulated fly entered the odour many times, few were sufficiently long (≧1 sec) to write in an entry memory. (**g**) Spatial map showing the average crosswind component of entry-velocity unit vectors at each pixel location, with red = 1, white = 0, blue = −1. Each dot represents the average crosswind entry for a specific pixel location, n = 1593 simulations. (**h**) Memory crosswind predictivity as a function of alongwind location of plume entry. Predictivity is calculated as the product of the crosswind components of the entry-velocity and entry-memory vectors after normalization to unit length (see Methods). Error bar shows SEM. (**i**) A representative simulated trajectory of the model without memory, with red indicating epochs in odour and black out of odour overlaid onto a single plume snapshot. The average envelope of the plume is shown as a dashed line. (**j**) Comparison of simulated trajectories using the average fly model with (black) and without (red) entry memory. Left, path length of outside bouts as a function of distance along plume. Mean ± SEM, n = 1593 simulations. Right, probability of simulated trajectory making it to within 20 mm of the source (counted as a success) as a function of the alongwind location of the first plume entry (see Methods). Only trajectories with at least one effective entry are shown. Error bar: 95% Wilson score confidence intervals. Details of statistical analyses and sample sizes are given in Supplementary Table 1.

Memory mechanisms could be most advantageous if the structure of the plume is sufficiently coherent for entry angles to remain predictive. To assess memory dynamics in more naturalistic environments, we evaluated tracking in the context of a 300-mm surface plume^3,17^, scaled fivefold to better match the dimensions of our odour corridors and allow access to a larger number of excursions from the plume (Extended Data Fig. 13a). As is characteristic of naturalistic plumes^17^, the structure of the plume varied along its length—from a stable, coherent ribbon near the source to dynamic fragmented filaments downstream (Fig. 5a)—allowing us to evaluate tracking performance across distinct turbulence regimes. Both real and simulated flies tracked the plume successfully using a similar strategy, even when starting far downwind in the plume’s most turbulent region, where chance odour encounters occurred at incoherent entry angles and promoted an upwind drive that might support effective tracking even in the absence of memory (Fig. 5a,d, Extended Data Fig. 13c,d and Supplementary Video 4). As the plume narrowed and became more ribbon-like closer to the source, the crosswind component of entry directions became increasingly coherent (Fig. 5b,e). Accordingly, the entry memory generated by our model or inferred from real flies predicted crosswind entry direction more accurately in this region (Fig. 5c,f), producing more efficient return trajectories than did simulations without entry memory (Fig. 5g,h). Similar trends were observed with the unscaled plume (Extended Data Fig. 13e,f), although the benefit of memory was attenuated owing to the fewer effective entries available to generate a coherent memory. Flies thus appear to deploy multiple navigational strategies depending on plume statistics, but can use edge tracking when the turbulence of a plume is sufficiently low for entry angles to be predictive.

## Dynamic goals in the central complex

**Fig. 6:**
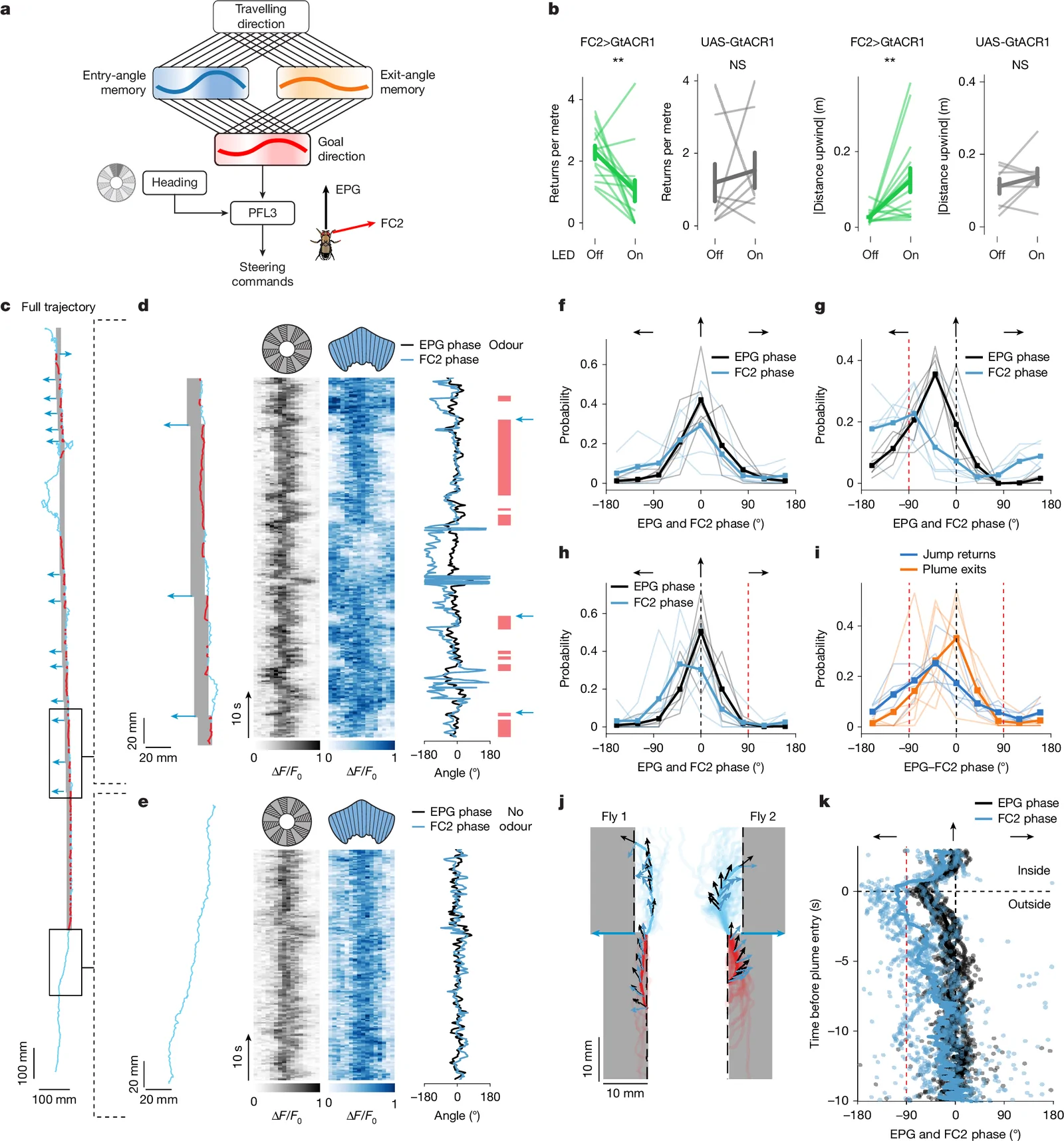
FC2 neurons signal the direction of the plume’s boundary before returns. **a**, Schematic of potential implementation of the model. Plume boundary crossings write in an entry- or exit-angle memory that is alternately read out in returning or leaving states. These memories specify a goal direction in FC2 neurons that is compared with the current heading by PFL3 neurons to drive corrective steering. **b**, Successful returns and upwind distance travelled by FC2>GtACR1 and control flies with LED off or on. Thin lines, individual flies; thick lines, mean ± s.e.m. (*n* = 9–14 flies). **c**, Representative experiment simultaneously imaging FC2 and EPG activity as flies transitioned from walking in clean air to tracking a 10-mm jumping plume. After the first 20 entries, the plume was periodically shifted 3 mm away during returns (blue arrows) to minimize incidental re-encounters. **d**,**e**, Representative epochs during edge tracking (**d**) and upwind walking before plume encounter (**e**). Left, trajectories; middle, EPG and FC2 activity (Δ*F*/*F*_0_); right, estimated EPG (black) and FC2 (blue) phases. Red bars indicate odour encounters; arrows indicate plume jumps. **f**, Distributions of EPG and FC2 phases during pre-odour upwind walking, centred around the upwind direction. **g**, Phase distributions in the 1 s preceding returns to the jumped plume, skewed towards the plume boundary direction (red dashed line). Black dashed line denotes upwind. **h**, Phase distributions in the 0.5 s preceding plume exits. **i**, Distribution of FC2–EPG phase differences during returns (blue) and exits (orange). During returns, FC2 phases lead EPG phases toward the plume boundary. **j**, Mean EPG and FC2 phase vectors overlaid on averaged exit and return trajectories for two representative flies. Transparent lines indicate individual trajectories. **k**, Progression of EPG and FC2 phases before returns to the jumped plume and after re-entry into the plume. Dots represent data from individual flies; bold lines, mean across flies. Red dashed line indicates the direction of the plume boundary. *n* = 6 flies for imaging experiments. NS, not significant; **P < 0.01. Statistical details are in Supplementary Table 1. The schematic in **a** is adapted with permission from ref. ^15^ (Cell Press).

Although our model is derived only from behaviour, its components map well onto the circuitry of the fan-shaped body (Fig. 6a), a region of the central complex that encodes signals that could guide navigation through its grid-like organization of intersecting columnar and tangential neurons. Columnar neurons integrate wind and heading signals to represent spatial variables^35,36,43,44^—such as a fly’s travelling direction—as vectors through sinusoidal population activity, whereas tangential neurons receive olfactory input and other contextual signals^38,45^ and are poised to modulate these vector representations during navigation. On the basis of this suggestive circuit architecture, we hypothesize that the memory of a fly’s entry direction is stored as a sinusoidal pattern in the strengths of plastic synapses between tangential and columnar neurons, analogous to other anatomically inspired models of angular memory formation in *Drosophila*^37,39^. Memory updates could be driven by tangential neurons that are activated at the time of plume boundary crossings^45^. Distinct odour-responsive tangential neurons could gate the use of exit- and entry-angle memories, allowing flies to recall different goal directions when they are in the leaving and returning states.

**Extended Data Fig. 14:**
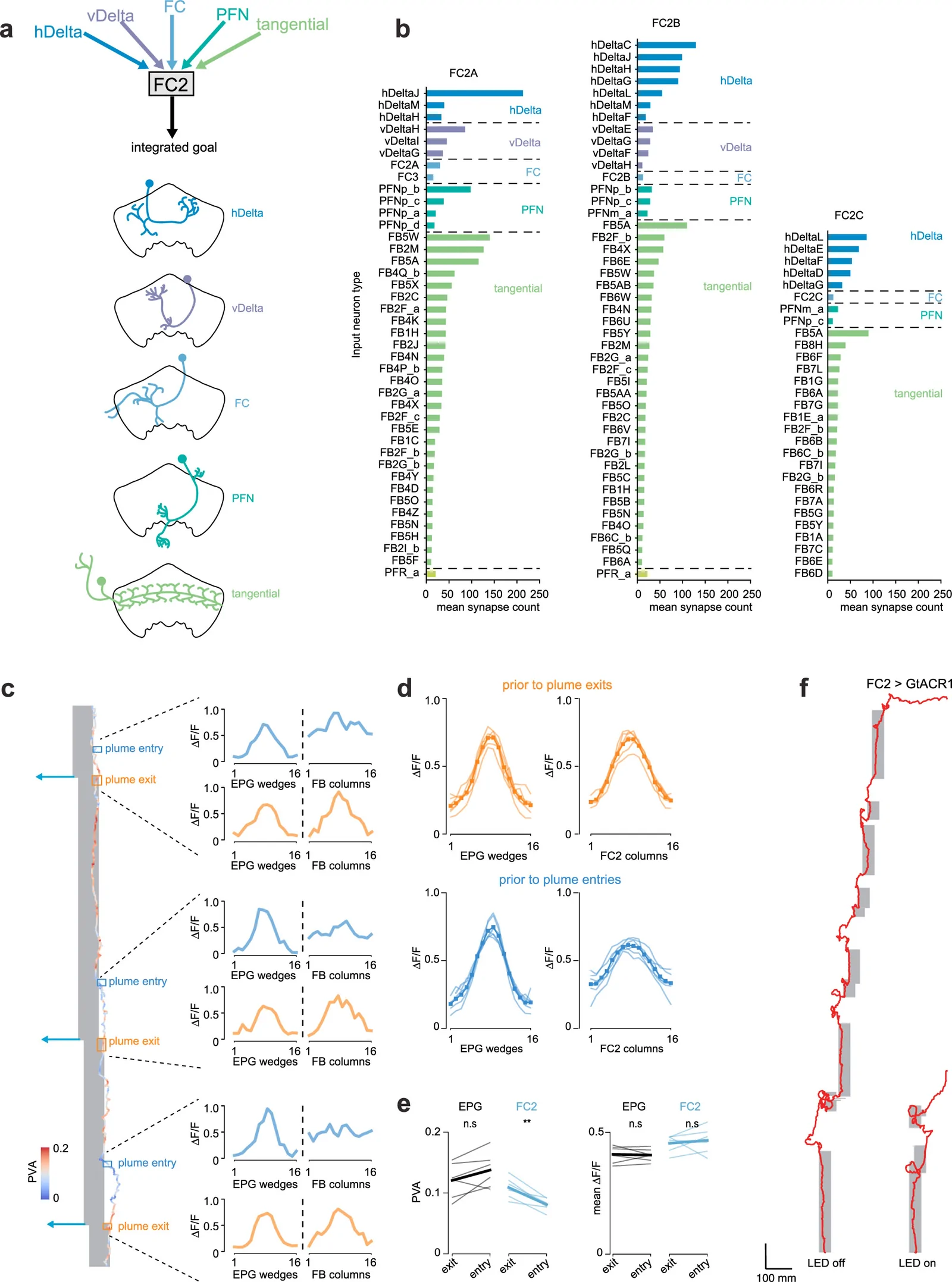
Dynamic goal signals in FC2 neurons during edge tracking. (**a**) Schematic showing different classes of neurons pre-synaptic to FC2. (**b**) Mean synapse count of inputs to different FC2 populations by input type (hemibrain connectome v1.2.1 https://neuprint.janelia.org/). (**c**) Representative experiment in which the activity of FC2 and EPG neurons were synchronously recorded as a fly tracks the edge of a 10 mm wide jumping plume (same fly as in Fig. 6c). Left: trajectory of fly as it makes three sequential excursions inside and outside of a jumping plume, coloured by population vector amplitude (PVA) of FC2 neurons across columns of the fan-shaped body. Note that the PVA provides a measure of the coherence of the bump in the FC2 population which is larger inside the plume than outside the plume. Right: Phase nulled EPG wedges or FC2 columns, averaged over 0.5 sec prior to plume exits (orange) and entries (blue). (**d**) Mean phase-nulled EPG wedges/FC2 columns during exits (orange) and entries (blue) to the jumped plume. (**e**) Comparison of phase coherence (mean PVA) and net activity (mean ΔF/F across all wedges/columns) at exits and entries to the jumped plume. n = 6 flies. (**f**) Representative experiment showing the trajectory of an FC2 > GtACR1 fly navigating a jumping plume in the absence (left) and presence (right) of optogenetic inhibition via LED illumination throughout the edge-tracking trial. **p < 0.01, ns, not significant p > 0.05. Details of statistical analyses and sample sizes are given in Supplementary Table 1.

To gain functional evidence for this model, we examined the activity of FC2 neurons—a columnar population that encodes a fly’s goal as a stable bolus of activity during menotaxis^6^, in which a fly walks at a constant bearing relative to an external landmark. FC2 neurons receive input from multiple tangential and columnar populations^38^ (Extended Data Fig. 14a,b) and directly synapse onto PFL3 neurons, an output pathway of the central complex that controls steering by comparing a fly’s current heading with its goal^6,37,38,40^. FC2 neurons are thus poised to flexibly represent a fly’s changing goals during edge tracking. To examine this possibility, we simultaneously recorded the activity of EPG and FC2 neurons as flies transitioned from walking in clean air to tracking a jumping plume, which promotes longer return paths and prevents flies from incidentally re-encountering the plume’s boundary (Fig. 6c–e).

As flies initially walked upwind in clean air, the phases of EPG and FC2 neurons remained mostly aligned (Fig. 6e,f), matching their relationship during menotaxis^6^. During edge tracking, however, the phase of FC2 neurons frequently diverged from the EPG signal (Fig. 6d). Notably, the FC2 phase tracked a fly’s current heading inside the odour and as it first exited the plume, but began to point in the direction of the plume’s boundary several seconds before the fly turned in that direction (Fig. 6d,g–k). The delay between the emergence of this FC2 signal and the subsequent turning of the fly (Fig. 6k) mirrors the time course of the corrective steering that flies show when an arbitrary goal angle is optogenetically imposed on this columnar population^6^. Consistent with a role in guiding returns, optogenetic silencing of FC2 neurons significantly impaired edge tracking (Fig. 6b and Extended Data Fig. 14f), paralleling the phenotype observed with EPG silencing. Edge tracking therefore requires both the neurons that express the entry-angle memory and the allocentric reference frame in which it is stored.

## Discussion

Odours serve as key navigational cues for many species, despite carrying no inherent directional information. Animals must therefore integrate chemical cues with spatial signals to track an odour to its source, a requirement that is thought to strongly shape the evolution of navigational circuits^46,47^. Indeed, the close evolutionary relationship of ancestral forebrain circuits, such as the piriform and entorhinal cortex in mammals^48–50^, and the extensive connectivity between the mushroom body and central complex in insects^38,45,51^, exemplify how diverse species have evolved the ability to store memories of their odour experience and bind these to internal representations of space.

Here we explored how flies rely on memory mechanisms to track along a plume’s boundary. Our analyses suggest that each time a fly enters a plume, it dynamically updates and stores an angular memory of its current heading direction relative to the wind. Consistent with this idea, FC2 neurons that encode a fly’s allocentric goal^6^ selectively signal the direction of the plume’s boundary during return trajectories. Edge tracking thus shares key computational features with the vector-based navigation used by central place foragers, such as ants and bees, to home to their nests^9,10^. One notable difference, however, is that unlike a nest, the boundary of an odour plume is dynamic. This could explain why edge-tracking flies store only the relative direction to the plume’s boundary, rather than its specific position. Consistent with this, flies do not revisit the site at which they last exited the plume but instead bias their trajectories in the remembered direction of the plume’s boundary as they continue to advance along its length. These results underscore that odours do not represent the ultimate ‘goal’ during plume tracking, but rather serve as chemical signposts that an animal can use to track towards the source.

During edge tracking, flies encounter a continuous odour corridor; however, behaviourally, they create a temporally intermittent experience by sampling its edge. Although edge tracking might minimize sensory adaptation^21,22^, flies seem to be relatively indifferent to odour concentration, suggesting that it is not the primary driver of this behavioural strategy. Rather, we propose that the plume’s lateral boundary—as the site of the steepest concentration gradient—holds special signficance, by providing maximal information about the plume’s structure^52^ even if it shifts. Indeed, neural networks trained to track a meandering plume adopt a plume-skimming strategy that mimics edge tracking^28^, demonstrating that following a plume’s boundary rather than the wind direction represents a particularly robust strategy. Exploiting environmental contrast by following the steepest sensory gradient has been proposed to underlie navigation across many biological scales, from the paths of axonal outgrowth^53^ to sonar localization by bats^54^, suggesting that edge tracking may represent a general algorithm.

For memories to have predictive value, their timescales must be aligned with the stability of the environment. Once formed, the memory of the plume’s edge appears to be persistently maintained, but can nevertheless be updated rapidly through additional odour encounters. Such flexibility is likely to be advantageous in more naturalistic plumes, where memory mechanisms might facilitate tracking only when the plume’s structure is sufficiently stable for entry angles to remain predictive. In more turbulent regimes, in which odour filaments arrive from erratic directions, edge tracking confers less benefit, and flies are likely to resort to purely reflexive strategies or other forms of short-term memory, such as using plume intermittency, to guide their navigation towards the odour source^11,55^. Different tracking algorithms are therefore likely to be dynamically recruited depending on the coherence and stability of the olfactory environment.

Our data suggest that central complex circuits, beyond encoding a single stable goal, can support the flexible formation and sequential deployment of several goals as flies weave into and out of the plume. In the fan-shaped body, distinct populations of odour-responsive tangential neurons^45^ are poised to modulate columnar neurons that encode spatial variables—such as current heading or travelling direction^35,36,38,39^ —and write in, then subsequently recall, an exit- or entry-angle memory. These distinct angular goals might be maintained in parallel and differentially accessed as a fly alternates between the leaving and returning state. Although FC2 neurons signal the entry-angle memory as flies return to the plume, we found no evidence that they encode the exit angle, suggesting that additional columnar neurons guide steering out of the plume during the leaving state. Alternatively, an explicit memory-based mechanism in the central complex might not be required. Rather, flies could reflexively surge upwind, simply by suppressing their entry-angle goal when they are in the plume. Consistent with this, FC2 neurons become rapidly aligned with the fly’s heading inside the plume, concordant with the observation that many columnar inputs to FC2 neurons are inhibited by odour (data not shown), allowing other pre-synaptic populations—such as PFN neurons that convey heading-related signals^35,36^—to dominate. Together, these observations highlight how FC2 neurons can dynamically switch between encoding heading-related signals inside the plume and encoding memory-related signals outside the plume, when flies only have access to the wind direction and stored entry angles to guide their navigation.

The alternation between different goals in the leaving and returning states could tune edge-tracking trajectories^10^, potentially giving rise to the striking variation we observe across individuals, with some flies adhering closely to the plume’s boundary and others taking more-exploratory, circuitous routes (Extended Data Fig. 6d). Tangential neurons that innervate different layers of the fan-shaped body integrate signals from distinct lateral horn and mushroom-body pathways^38,45^, which could allow them to dynamically regulate the strength of memory updating or the timescales of the leaving and returning states depending on the innate or learned valence of an odour or other contextual cues.

The central complex is thought to underlie navigation in many insect species, from the long-distance migrations of monarch butterflies to ants homing to their nest^9,10^. Here we reveal how flies dynamically update angular memories to store the direction of a plume, not their distance from it, exemplifying a basic building block for these more complex navigational feats.

## Methods

### Fly husbandry

Flies were maintained at 23–25 °C and 60–70% relative humidity under a 12-h light–dark cycle. The quality and composition of the fly food was a key factor for eliciting robust edge-tracking behaviour in tethered flies, particularly during imaging experiments. We found that flies that were raised for several generations on Wurzburg food^39^ exhibited robust edge tracking more consistently than did those raised on standard cornmeal–agar–molasses food. All behavioural experiments were performed using starved flies, which were removed from food and placed in vials containing only a water-soaked KimWipe or cotton plug for 16–24 h before tethering. For optogenetic experiments, flies were reared in complete darkness. Two days before an experiment, one-to-two-day old flies were transferred to a food vial containing 0.4 mM all-*trans*-retinal (Sigma, R2500). Sixteen to twenty-four hours before an experiment, flies were removed from food and placed in a vial containing a Kimwipe soaked in 1–2 ml of 0.2 mM all-*trans*-retinal in water.

#### Detailed fly genotypes

For all behavioural experiments examining edge tracking of an odour plume (Figs. 1, 2, 4 and 5 and Extended Data Figs. 1–3, 5–7, 9, 11 and 12), we used Canton-S. For perturbations of EPG neurons (Fig. 3), we used the SS00098 EPG split Gal4 line: 19G02-p65AD/+; R22E04-DBD/UAS-GtACR1-EYFP. For functional imaging of EPG neurons (Figs. 3 and 6 and Extended Data Fig. 14), we used the 60D05-Gal4 driver: UAS-jGCaMP7f/+; 60D05-Gal4/+. For perturbations of FC2 neurons (Fig. 6 and Extended Data Fig. 14), we used VT065306-AD; VT029306-DBD/UAS-GtACR1-EYFP. For functional recording of FC2 neurons (Fig. 6 and Extended Data Fig. 14) we used VT065306-AD/UAS-syt-jGCaMP7f; VT029306-DBD/60D05-Gal4. For optogenetic activation of olfactory sensory neurons (Extended Data Fig. 4), we used: UAS-Chrimson.mVenus/Orco-GAL4, w^1118^ UAS-CsChrimson.mVenus, Orco-GAL4, w*.

*Drosophila stock sources*. EPG split line: 19G02-p65ADZp (in attP40); R22E04-ZpGdbd (in attP2) (Bloomington *Drosophila* Stock Center (BDSC) 93169); FC2 split line: VT065306-AD; VT029306-DBD (gift from G. Maimon); R60D05-Gal4 (BDSC 39247); 10XUAS-sytGCaMP7f (attP2) (BDSC 94619); 20XUAS-IVS-CsChrimson.mVenus(attP18) (BDSC 55134); Orco-GAL4.C(142t52.1), w[*] (BDSC 23909); UAS-GtACR1.d.EYFP(attP2) (BDSC 92983); 20XUAS-IVS-jGCaMP7s(VK00005) (BDSC 79032).

### Fly tethering and dissection

All assays were performed using 1–5-day old female flies. Flies were briefly anaesthetized (less than 10 s) using CO_2_ and tethered to a custom-milled fly-plate similar to what has been previously described^56^. Flies were mounted to the fly-plate using a strand of hair or a single paintbrush bristle, which was used to secure their heads and subsequently their bodies to the plate before gluing the eyes and thorax using UV-curable glue. In all assays, the filament was removed after successful tethering and flies were placed in a dark, climate-controlled space (25 °C, 40–60% relative humidity) to recover for 15–30 min before the start of the experiment. Flies were then transferred to the closed-loop apparatus and allowed to walk freely on the ball for at least 15 min before experiments.

For functional imaging experiments, fly preparation varied accordingly. After tethering, the proximal portion of the extended proboscis was glued to minimize movement during recording while allowing the distal portion of the mouthparts to move freely during experiments. Flies were provided a recovery period of 30–120 min after tethering. After the recovery period, the fly-plate was then filled with saline (108 mM NaCl, 5 mM KCl, 2 mM CaCl_2_, 8.2 mM MgCl_2_, 4 mM NaHCO_3_, 1 mM NaH_2_PO_4_, 5 mM trehalose, 10 mM sucrose and 5 mM HEPES sodium salt, pH 7.5 with osmolarity adjusted to 275 mOsm). The cuticle covering the posterior portion of the brain was then cut using a 30-gauge needle and removed using forceps to facilitate optical access to central complex structures. Obstructing trachea were removed taking care to not damage the antennae or the antennal nerves. Flies were subsequently transferred and allowed to walk on the ball for at least 15 min.

### Preparation of the olfactory environment

A virtual olfactory environment was created for walking tethered flies using a previously described^16^ closed-loop olfactory system with the addition of custom Python scripts that allowed for two-dimensional (2D) rendering of odour plumes.

#### Tethered locomotion

For tethered locomotion experiments, a spherical treadmill based on previous studies was designed. A 6.0–6.5-mm-diameter ball was shaped from LAST-A-FOAM FR-4618 (General Plastics) by a custom-made steel concave file. The ball rested in an aluminium base with a concave hemisphere 6.75 mm in diameter with a 1-mm channel drilled through the bottom and connected to an airflow. The ball was recorded at 60–61 frames per second using a Point Grey Firefly camera (Firefly MV 0.3 MP Mono USB 2.0, Point Grey, FMVU03MTM-CS) with an Infinity lens (94-mm focal length) focused on the ball, with illumination from infrared LED lights. Ball rotation was calculated in real time using FicTrac software^57^ running on computers with processor speeds of at least 3 GHz.

#### Closed-loop arena

The heading of the fly, as calculated by FicTrac, was transmitted to a RaspberryPi 4 through a serial port. Custom Python code was used to translate heading into tube position, controlled by motors, as described below. The closed-loop air delivery system was custom designed using OnShape (https://www.onshape.com) and 3D printed using VisiJet Crystal material at XHD resolution in a 3D Systems ProJet 3510 HD Plus. O-ring outside dimension and inside dimension gland surfaces were designed with excess material for printing and then manually modified on a lathe for improved RMS (surface) finishing. The 360° tube rotation was driven by a bipolar stepper motor (SureStep DC integrated NEMA 17 stepper) controlled through an integrated driver and coupled by a Dust-Free Timing Belt (XL Series, 1/4″ width, McMaster-Carr, 1679K121, trade no. 130 × L025) to the rotating tube system, which rotated mounted on an Ultra-Corrosion-Resistant Stainless Steel Ball Bearing (3/4″ shaft diameter, 1-5/8″ inner diameter, McMaster-Carr, 5908K19). The air channel was kept airtight using oil-resistant O-rings (1/16″ fractional width, dash no. 020, McMaster-Carr, 2418T126). Motor rotation was measured by a rotary encoder (CUI Devices, AMT10 Series) that was used to correct for skipped steps.

#### Airflow and odour delivery

Odour delivery was achieved by directing a continuous stream of humidified clean air through a 2-mm-diameter tube made of VisiJet Crystal material directed at the fly’s antennae. An anemometer (Kanomax 6006-DE) was used to ensure that airspeeds reaching the fly were maintained at 15–25 cm s^−1^. Air to the system was first passed through a charcoal filter and humidified by bubbling the air through a deionized water reservoir. The airflow was then split between the spherical treadmill and three mass flow controllers (MFCs; Alicat, MC-Series, 1000SCCM). Downstream of each MFC, air passed through the headspace of odour vials containing either ACV (Heinz) or deionized water. The ratio of air entering each vial was dependent on the fly’s position relative to the odour plume, such that when a fly is outside the boundaries of the plume, 100% of the air is directed into the water vial; when the fly is within the boundaries of the plume, the air is directed at an experimenter-determined ratio between the water vial and the odour vials. Air streams coming from the odour vials then merge with a Y-connector before entering the air delivery system. Thus, by controlling how much of the air passes through each vial, the MFCs control the total odour concentration of the airstream reaching the fly. The MFCs were controlled using a RaspberryPi 4 running custom Python scripts.

#### Optogenetic stimulation

All optogenetic experiments were done on flies with intact cuticles. Flies were reared in the dark, transferred to retinal food and tethered as described above. For optogenetic experiments, a fibre-coupled LED was controlled by a T-cube LED driver (Thorlabs, LEDD1B) to deliver light to the fly’s head in closed loop with its behaviour. For the activation of Orco^+^ sensory neurons, a 660-nm (red) LED (Thorlabs M660FP1) was turned on (0.863 μW mm^−2^) when the fly was inside the fictive odour plume. For EPG inhibition experiments, a 530-nm (green) LED (Thorlabs M530F2) was turned on (0.752 μW mm^−^^2^) for the duration of the entire trial.

#### Closed-loop parameters

Behavioural measurements (sampled at 60 Hz) were determined by the rotation of the foam ball and obtained from FicTrac (*x* position, *y* position, heading, roll, pitch and yaw). These were saved alongside MFC flow values, experimental variables (odour on, LED on) and a time stamp in a single .log file.

### Edge-tracking behavioural assays

Tethered behavioural assays were performed in a dark, climate-controlled room (25–27 °C, 40–60% relative humidity). The closed-loop olfactory system was enclosed in a black tarpaulin as a further shield from other potential light sources, such as computer monitors and indicator lights on system hardware. To prevent odour build-up in the enclosed space, a vacuum line (60 cm s^−1^) was placed at the back of the enclosure as an exhaust. Unless otherwise stated, all experiments started with a 2–5-min baseline period in which flies walked in clean air delivered in closed loop. At the end of this baseline period, flies were placed at the centre of the odour plume’s short axis, with the exception of the 90° plume, in which flies were placed at the centre of the odour plume’s long axis. The geometry of the plume (0°, 45° or 90°) was determined before the start of the experiment. Unless otherwise stated, each plume’s short axis measured 50 mm and each long axis 1,000 mm. For EPG and FC2 imaging experiments, the plume’s short axis was 10 mm. For EPG and FC2 silencing experiments, all flies completed paired LED-on and LED-off trials in pseudo-random order. For the jumping-plume experiments in Figs. 2d,h, 3e,f and 6 and Extended Data Figs. 5i, 6, 7 and 14, the edge of the plume was shifted 20 mm in the direction opposite to the fly’s heading as the fly exited the plume. Owing to the propensity of flies to engage in straighter trajectories because of the heat of the two-photon laser^6^, during functional imaging of FC2 neurons (Fig. 6 and Extended Data Fig. 14), once the fly had made 20 plume entries, the plume was jumped 3 mm on every third subsequent entry. For all experiments, trials were terminated if one of these three criteria was met: (1) the fly travelled the length of the long axis of the plume provided (1,000 mm), except in the case of FC2 imaging, in which experiments were terminated at 2,000 s; (2) the fly travelled more than 500 mm perpendicular to the plume’s edge; (3) the fly travelled 100 mm downwind from its starting position on the plume.

A small fraction of tethered flies (less than 10%) were excluded from the study because they did not acclimate to walking on the ball, because they did not show a behavioural response to ACV (by turning upwind and increasing their speed) or because continuous tracking of the trajectory was lost by FicTrac.

#### Odour concentration-gradient plumes

Gradient plumes were created with the same dimensions (50 mm by 1,000 mm) but featured one of three odour concentration gradients. (1) Vertical plume with increasing upwind odour concentration: odour concentration increased linearly in the upwind direction, starting at 10% ACV at the plume’s downwind end (0 mm) and reaching 100% ACV at the upwind end (1,000 mm). (2) Vertical plume with decreasing upwind odour concentration: odour concentration decreased linearly in the upwind direction, starting at 100% ACV at the downwind end (0 mm) and diminishing to 10% ACV at the upwind end (1,000 mm). (3) 90° plume with crosswind odour concentration gradient: odour concentration varied linearly along the lateral axis, increasing from 10% to 100% ACV in one crosswind direction or decreasing from 100% to 10% ACV in the opposite direction over 1,000 mm. Flies were at first positioned at the interface between these two gradients, allowing them to track up or down the concentration gradient in the crosswind direction.

#### Graded lateral plumes

Plumes with graded lateral concentration profiles followed a Gaussian profile characteristic of naturalistic plume envelopes. To make these plumes most comparable to the 50-mm constant-concentration odour corridor, the peak odour concentration was set to 20% at the plume’s midline, and the plume’s width was adjusted to 160 mm so that the half-maximal concentration occurred at ±25 mm from the plume’s midline. Flies tested in a plume with a graded lateral boundary were also tested in a plume with a sharp lateral boundary (Extended Data Fig. 3) in pseudo-random order.

#### Optogenetically generated fictive odour plumes

Fictive odour plumes were the same dimensions as a vertical odour plume (50 mm by 1,000 mm). Flies were positioned in the centre of the plume’s short axis and optogenetic stimulation was provided by a 660-nm LED whenever the fly was within the plume’s boundaries.

#### Disappearing plumes

Flies tracked a constant-concentration plume for 10 min. After this period, during their first trajectory outside the plume, the plume was removed, leaving the fly walking in closed-loop wind without further olfactory input.

#### Dynamic plumes

Flies were presented with a looped video of a previously recorded surface plume that was binarized to match the composition of concentrations in odour corridors. The video spatial dimensions were scaled up by bicubic interpolation to provide a larger stimulus in which flies were likely to make more than one to five returns (total alongwind length was 2,030 mm instead of the original 300 mm), and the replay speed was scaled down (see ‘Dynamic plume analysis’). Flies were initialized at a position 1,700 mm downwind and at the centre line of the fictive source. During exploration, odour delivery was referenced to the fly’s spatial position relative to the video *X*, *Y* dimensions and to the presence of odour at that referenced pixel in the frame of the *T* dimension of the video matching the experimental time elapsed. Given the irregular plume structure and the time-varying intermittency of odour filament encounters even when the fly was stationary, many odour encounters were shorter than 1 s. Because the odour concentration required around 1 s to reach set point in our system (Extended Data Fig. 1a), each odour encounter was programmed to deliver a 1-s odour signal at minimum. Each experiment was stopped when (a) 20 min had elapsed; (b) the fly wandered more than 500 mm from the outer boundaries of the plume envelope and did not return within 10 min; or (c) the fly located the source (10 out of 12 flies).

#### Replay experiments in which the odour sequence was replayed back to the fly in open loop

For odour-replay assays, we allowed flies to track a 90° plume for 10 min and recorded the time stamps of odour onset and offset in a separate log file. This file was used to generate the temporal sequence of odour pulses delivered during the replay epoch of the experiment, which was initiated 10 s after the edge-tracking epoch back to the same fly. The same temporal sequence of odour pulses was also presented to a naive fly that had never edge tracked. Consequently, for every replay experiment, we collected data from one naive fly, and each naive fly received a distinct temporal sequence of odour pulses defined by the matched replay experiment. During replay, wind was delivered in closed loop but the fly’s fictive position had no effect on the timing of odour delivery.

#### Operant training paradigm

The operant training paradigm consisted of three phases:

1. Initial edge-tracking phase: flies were allowed to track a 45° plume. After tracking for 250 mm along the plume’s edge, the plume disappeared as flies were on their next outside trajectory.
2. Operant training phase: during training, a fly’s average heading and speed was continuously calculated over a 2-s sliding window. Flies triggered the delivery of odour when their average heading was in a 45° range of entry angles (either −90° to −135° or 90° to 135°, depending on whether training was in the same direction as the initial plume or in the opposite direction; Extended Data Fig. 11a) while sustaining an average speed higher than 2 mm s^−1^ over that 2-s sliding window, to ensure that the flies did not trigger odour stimulation while standing still. Odour was delivered for a minimum of 2 s and terminated when flies oriented in the 90° range of upwind exit angles (−45° to 45°) while sustaining an average speed higher than 2 mm s^−1^. The number of training epochs (for example, one or five odour stimulation periods) during this training phase, and whether they were in the same or opposite direction as the initial plume segment, were determined before the start of the experiment.
3. Test edge-tracking phase: after completing the predetermined number of training epochs, flies were positioned at the centre of the short axis of the future test plume, oriented opposite to the direction of the 45° plume they had tracked during the initial phase. Note that after flies completed this training phase, they had to enter the test odour using the same heading that was used for the training phase, and so effectively had an additional epoch of reinforcement. In the test phase, the plume extended 20 mm below the fly’s starting position to increase the chances of it successfully entered the test plume.

### Analyses of edge tracking behaviour

#### Trajectory averages

To construct average trajectories (Figs. 1e, 2e–h, 3f and 6j and Extended Data Figs. 5e, 6a and 7b), *x* and *y* coordinates for each outside or inside bout were upsampled to contain 10,000 points through linear interpolation between data points using the pandas library in Python. This allowed us to average *x* and *y* coordinates regardless of the length of the inside or outside bout. Upsampled *x* and *y* coordinates were averaged to produce one set of (*x*, *y*) coordinates for each fly. To construct an average across flies, the averages generated from individual flies were averaged together.

#### Entry and exit angles

Entry and exit angles were determined by calculating the mean travelling direction in the 0.5 s before and 0.5 s after crossing the plume’s boundary.

#### Analysis of inbound and outbound segments

The outside trajectories of flies tracking a vertical plume were simplified into straight-line segments using the Ramer–Douglas–Peucker (RDP) algorithm^58^, which simplifies a set of *x*, *y* coordinates by iteratively reducing the number of points in the trace. The parameter *ε* determines the maximum allowed distance between the simplified and original trajectories and was 1 mm. These trajectories were then averaged using the linear interpolation method described above providing a single average trajectory for each fly. From this average trajectory, ‘outbound’ (away from the edge) and ‘inbound’ (back towards the edge) path lengths were calculated as follows: (1) outbound path length was calculated as the path length from the point of exiting the plume to the farthest point orthogonal to the plume’s edge; and (2) inbound path length was calculated as the path length from the farthest point orthogonal to the plume’s edge back to the plume’s boundary. These three points—plume exit point, farthest point and plume entry point—define a triangle, the upwind angles of which define inbound and outbound angles as indicated in Fig. 1f. For analyses of simulated inbound and outbound trajectories in a random search model (Extended Data Fig. 5d,e), we took the total number of outside bouts for each fly and then randomly selected the same number of outside simulated outside trajectories. From this collection of random trajectories, we used the method above to generate an average trajectory and then calculated outbound and inbound lengths.

#### Definition of anemotaxis

Trajectories of flies before ACV exposure (pre-air period) were divided into anemotaxis bouts, defined in a similar manner to menotaxis bouts in a previous study^6^. Flies’ trajectories were simplified into straight-line segments using the Ramer–Douglas–Peucker algorithm (*ε* = 10 mm). Segments of length greater than 50 mm were assigned as anemotaxis bouts. See Fig. 6e,f.

#### Random-walk model

We simplified the outside trajectories of 40 flies tracking a vertical plume using the Ramer–Douglas–Peucker algorithm (*ε* = 1 mm) and extracted the straight-line distances (run lengths) that flies travelled between successive turns and the angles between consecutive straight-line vectors (turn angles) to capture the changes in heading direction. Run lengths and turn angles were each stored in a separate library. We simulated random-walk trajectories by randomly sampling from the two empirical distributions of run lengths and turn angles. Simulated flies started at the plume’s edge and the initial run length was assigned a heading that was drawn randomly from the initial segment heading directions from the plume (Extended Data Fig. 5b). At each step: (1) a run length was randomly sampled from the empirical distribution of run lengths; (2) a turn angle was randomly sampled from the empirical distribution of turn angles; and (3) the simulated fly moved forwards by the run length and then adjusted its heading by the turn angle.

The simulation continued until the simulated fly either re-encountered the plume edge or exceeded a maximum distance travelled of 500 mm in the direction perpendicular to the plume’s edge, which is the same maximum distance that flies were allowed to travel in experiments before a trial was terminated.

To reflect the natural tendency of flies to walk upwind even in the absence of odour cues^16^, we modified the random-walk model to include an upwind bias. We adjusted the turn direction on the basis of a sinusoidal probability function whose magnitude scaled between 0 and 1 (value *a*). When *a* = 0, the probability of turning left or right is the same for all heading directions. As the values of *a* increase from *a* = 0 to *a* = 1, the fly becomes progressively more biased to turn left when it is facing right, and to turn right when facing left. The value of *a* was selected to match that of real flies (Extended Data Fig. 5c), mirroring the upwind bias observed in actual flies outside the odour plume.

Given that random exploration outside the plume should not depend on the geometry of the plume tracked, the same model was used to simulate outside trajectories for flies tracking the 45°, 90° and jumping plumes, drawing from the same empirically derived libraries of run lengths and turn angles (Extended Data Fig. 5g–i). The only difference between the models was the initial heading, which was sampled from the initial segment heading directions for the 45° and 90° trajectories.

### Functional imaging

All functional imaging experiments were performed on an Ultima or Investigator two-photon laser scanning microscope (Bruker) equipped with galvanometers driving a Chameleon Ultra II Ti:Sapphire laser. Emitted fluorescence was detected with GaAsP photodiode (Hamamatsu) detectors. Images were acquired with an Olympus 40×, 0.8 NA or 20×, 1.0 NA objective at 512 × 512 pixel resolution. The laser was tuned to 920 nm for all experiments. The out-of-objective power for all experiments was around 15 mW and never exceeded 25 mW. For volumetric imaging, three to five slices were recorded using a piezoelectric Z-focus (Bruker) at a rate of 3–10 Hz.

#### Data alignment

To align the imaging and behavioural data, we used a 3.3-V digital pulse from a RaspberryPi to initiate recording of an imaging stack through the PrairieView (Bruker) I/O interface. The RaspberryPi continued to send 3.3-V pulses every 10 s, which were recorded with PrairieView for confirmatory alignment of the imaging and behavioural data. Behavioural variables were captured at 60–61 frames per second, whereas neural activity (fluorescence) was captured at 3–10 frames per second. To align these two distinct time series, a standardized and regular time series was generated for each trace containing 100-ms time bins (0, 100, 200, 300 and so on). Behavioural data were binned at the centre of each time point and averaged. Imaging data were upsampled using linear interpolation at the same 100-ms time bins. Custom Python scripts were used to synchronize and log incoming FicTrac variables and outgoing MFC and motor commands, allowing us to align the wind direction, odour concentration, behavioural data and imaging frames.

#### Image registration and regions of interest

All images were stored as individual tiff files, which were registered using the StackReg function of the pystackreg library. Regions of interest were drawn using custom Python scripts, Fiji or a custom MATLAB GUI. To quantify EPG neuron activity recorded from the protocerebral bridge, we followed the methods described in a previous study^35^ and manually defined each of the 16 glomeruli from the registered stacks corresponding to each imaging plane.

FC2 neuron activity during edge tracking was recorded in the fan-shaped body. EPG neuron activity in the ellipsoid body was recorded synchronously to allow for direct comparison of their phase relationships during an experiment. To quantify FC2 neuron activity, we manually defined the lateral borders of the fan-shaped body, extended these lines down to a point and divided the arc defined by these intersecting lines into 16 wedges. Pixels were assigned to each wedge if they resided within the wedge and within the footprint of the FC2 neurons, defined by masks that were drawn manually in each imaging layer on the basis of their arborization pattern. To quantify EPG neuron activity recorded in the ellipsoid body, we divided the ellipsoid body radially into 16 sub-wedges from a manually defined centre. Pixels were assigned to each wedge if they resided within the wedge and within the footprint of the EPG neurons, defined by masks that were drawn manually in each imaging layer on the basis of their arborization pattern.

Δ*F*/*F*_0_ values were determined by the formula (*F − F*_0_)/*F*_0_, where *F* is the mean pixel value within a wedge or glomerulus and *F*_0_ is the mean of the lowest 10% of *F* values.

#### Neuronal phase analysis

Phase was determined by two methods. For recordings of EPG neurons in the protocerebral bridge, phase was calculated as described previously^59^. For each time point, the Fourier transform was taken on the Δ*F*/*F*_0_ activity vector of the 16 glomeruli. Phase was determined as the phase of the Fourier spectrum at a period of 8, the peak periodicity of the power spectrum. For recordings of EPG neurons in the ellipsoid body and FC2 neurons in the fan-shaped body, phase was defined as the population vector average of wedge signals^31,35^. In summary, the 16 wedges of the ellipsoid body and fan-shaped body were assigned angles from −180° to 180°, equally dividing 360° of azimuthal space. Angles were assigned so that ellipsoid body and fan-shaped body wedges were in correspondence as per the connectome^38^. In the ellipsoid body, wedges were counted anticlockwise starting from the most ventral wedge to the right of the midline viewed from posterior to anterior. In the fan-shaped body, wedges were counted from right to left midline viewed from posterior to anterior. For each time point, each wedge was assigned a vector with length equal to the Δ*F*/*F*_0_ value and direction given by its assigned angle. Phase was the angle of the population vector average of these vectors at each time point.

#### Phase nulling and bump amplitude assessment

Phase nulled EPG and FC2 signals were calculated as described previously^35,59^. At each frame, we rotated the signal by the estimated phase of the activity bump in that frame, such that all bumps were aligned. To compare EPG bumps recorded in the protocerebral bridge in and out of odour, we separated frames corresponding to when the fly was in or out of the odour and averaged 122 together with the signal from all these frames to get the average bump profile. The mean of phase-nulled FC2 and EPG bumps in Extended Data Fig. 14d was taken over a 0.5-s window before entries to the jumped plume and the preceding plume exit.

FC2 and EPG bump amplitude was assessed by two measures: the mean Δ*F*/*F*_0_ across wedges and the population vector amplitude (PVA). Both measurements were taken over the same time period for the assessment of phase-nulled bumps. PVA was calculated as described previously^6^. The 16 wedges of the ellipsoid body and fan-shaped body were assigned activity-weighted vectors as for neuronal phase analysis (see above). PVA was calculated as the hypotenuse of the mean vector across wedges.

#### Phase calibration to allocentric coordinates

The anatomical locations of activity bumps in the central complex correspond to azimuthal orientations in allocentric space. However, the mapping from anatomical to external coordinates varies between individuals and can remap during an experiment^31^. To produce EPG and FC2 signals aligned to external coordinates, we rotated phase and wedges by an offset at each time point, such that phase angle 0° was aligned upwind and the central two wedges corresponded to 22.5° to the left and right of the upwind direction (Fig. 6d,e). The offset was the rolling mean (over 2 s) of the difference between the EPG phase and the fly’s heading, because the EPG signal provided a faithful neural correlate of heading relative to the wind (Fig. 3b–d). To rotate wedges, this offset was converted to an integer value varying between −8 and 8 by rounding to the nearest 22.5°. The same offset was applied to EPG and FC2 signals.

### Model

#### Time discretization

We bin the data into time intervals of 0.2 s and run the behavioural model in discrete time steps with this same interval. Because we are modelling the motion of the fly, we exclude data points when the fly’s speed is less than 1 mm s^−1^.

#### Edge-crossing events for memory update

To avoid spurious events and to account for the finite width of the odour boundary, we define an edge crossing as when a fly is on one side of the odour boundary for three consecutive time points and then on the other side for three consecutive time points. We define the fly’s velocity unit vector at the time of the crossing, $\hat{{\bf{v}}},$ as its velocity averaged over this five-time-step interval (1 s), normalized to unit length.

#### State

The model has two states: returning and leaving. When the fly leaves the odour plume in the leaving state, a time interval is drawn from a negative binomial distribution NB(*r*_leave_, 1 − *p*_leave_) and, after that number of time steps, the state switches to the returning state if the fly is still out of the plume. When the fly enters the odour plume in the returning state, a time interval is drawn from a negative binomial distribution NB(*r*_return_, 1 − *p*_return_) and, after that number of time steps, the state switches to the leaving state if the fly is still in the plume. Otherwise, no transitions occur. The parameters *r*_leave_, *p*_leave_, *r*_return_ and *p*_return_ are determined by fitting to data by the procedure described below.

#### Memory

The model stores 2D vector memories of plume exit and entry directions. The entry-memory vector **m**_entry_ is updated at the time of each entry by

$${{\bf{m}}}_{\mathrm{entry}}\leftarrow {a}_{\mathrm{entry}}{{\bf{m}}}_{\mathrm{entry}}+{b}_{\mathrm{entry}}\hat{{\bf{v}}}+{{\boldsymbol{\epsilon }}}_{\mathrm{entry}},$$

where $\hat{{\bf{v}}}$ is the entry-velocity unit vector defined above, and the two components of **ϵ**_entry_ are random variables drawn from a normal distribution with zero mean and variance ${{\sigma }}_{{\rm{entry}}}^{2}$. The exit memory vector **m**_exit_ is updated at the time of each exit by

$${{\bf{m}}}_{\mathrm{exit}}\leftarrow {a}_{\mathrm{exit}}{{\bf{m}}}_{\mathrm{exit}}+{b}_{\mathrm{exit}}\hat{{\bf{v}}}+{{\boldsymbol{\epsilon }}}_{\mathrm{exit}},$$

with $\hat{{\bf{v}}}$ the exit-velocity unit vector and **ϵ**_exit_ drawn from a normal distribution with zero mean and variance ${{\sigma }}_{{\rm{exit}}}^{2}$. In simulations, we ignore updates for which the exit $\hat{{\bf{v}}}$ is more than 60° away from the upwind direction. This follows the intuition that flies have an innate tendency to exit upwind. Other than during these plume boundary crossings, **m**_entry_ and **m**_exit_ remain unchanged. For real flies, the values of the memory vectors at the beginning of a trajectory depend on unknown previous history. We therefore model **m**_entry_(0) as the null vector and **m**_exit_(0) with a trajectory-specific Gaussian prior *N* $({{\boldsymbol{\mu }}}_{0},{\sigma }_{0}^{2}I)$ during fitting.

The parameters *a*_entry_, *b*_entry_, ${\sigma }_{{\rm{entry}}}^{2},$ *a*_exit_, *b*_exit_, ${\sigma }_{{\rm{exit}}}^{2}$, **μ**_0_ and ${\sigma }_{0}^{2}$ are determined by the fitting procedure described below. During fitting, nonzero values of ${\sigma }_{0}^{2}$, ${\sigma }_{{\rm{entry}}}^{2}$ and ${\sigma }_{{\rm{exit}}}^{2}$ allow unexplained variation of latent memories. However, during simulation, we set ${\sigma }_{0}^{2}={\sigma }_{{\rm{entry}}}^{2}={\sigma }_{{\rm{exit}}}^{2}=0$ to test the effectiveness of our hypotheses without introducing extra noise into the memories. When simulating from models fitted to a single trajectory, as in Extended Data Fig. 8d, we set the initial exit memory to **μ**_0_. When simulating from the average fly model (parameters computed from multiple trajectories; Fig. 4b), we set the upwind component of the initial exit memory to a fixed value ${w}_{\parallel }$ equal to the median of the upwind components of** μ**_0_ from all training trajectories, and we sample the crosswind component from a uniform distribution over the range from −2*w*_⊥_ to +2*w*_⊥_, where *w*_⊥_ is the median of the absolute value of the crosswind components of **μ**_0_ from all training trajectories.

#### Velocity update

In the leaving state, velocity is updated at every time step with a decay term, a bias towards the current exit memory and a noise term,

$${\bf{v}}(t)={c}_{\mathrm{leave}}{\bf{v}}(t-1)+{(1-c}_{\mathrm{leave}}){{\bf{m}}}_{\mathrm{exit}}+{{\boldsymbol{\xi }}}_{\mathrm{leave}}(t),$$

with ${{\boldsymbol{\xi }}}_{\mathrm{leave}}(t)$ drawn from a Gaussian distribution with zero mean and variance ${\sigma }_{\mathrm{leave}}^{2}$. Similarly, in the returning state, the velocity update is

$${\bf{v}}(t)={c}_{\mathrm{return}}{\bf{v}}(t-1)+{(1-c}_{\mathrm{return}}){{\bf{m}}}_{\mathrm{entry}}+{{\boldsymbol{\xi }}}_{\mathrm{return}}(t),$$

with ${{\boldsymbol{\xi }}}_{\mathrm{return}}(t)$ drawn from a Gaussian distribution with zero mean and variance ${\sigma }_{\mathrm{return}}^{2}$. The parameters *c*_leave_, *c*_return_, ${\sigma }_{\mathrm{leave}}^{2}$ and ${\sigma }_{\mathrm{return}}^{2}$ are determined by the fitting procedure described below.

#### Variational inference and parameter learning

For each trajectory, we fitted a trajectory-specific model using a variational expectation-maximization procedure. In the variational E-step, we inferred the binary leaving and returning states* z*_1:*T*_, entry memories **m**_entry,__1:*T*_ and exit memories **m**_exit__,1:*T*_ over the *T* time steps of the trajectory, while holding the model parameters fixed. Because the exact posterior *p*(*z*_1:*T*_,**m**_entry__,1:*T*_,**m**_exit,1:*T*_|**v**_1:*T*_,*o*_1:*T*_) is intractable, where **v**_1:T_ denotes observed velocities and *o*_1:*T*_ denotes odour observations, we used the mean-field approximation *q*(*z*_1:*T*_,**m**_entry__,1:*T*_,**m**_exit,1:*T*_) = *q*(*z*_1:*T*_)*q*(**m**_entry__,1:*T*_)*q*(**m**_exit,1:*T*_), following a previous study^41^. We optimized this variational distribution by coordinate ascent on the evidence lower bound (ELBO). In the M-step, we updated the model parameters by maximizing the ELBO while holding the current variational distribution fixed. We alternated between these two steps until the ELBO converged. When reporting inferred memories from real fly trajectories (Figs. 4g and 5f and Extended Data Figs. 8h, 10f and 12d), we used the mean of trajectory-specific variational memory distributions *q*(**m**_1:*T*_).

State durations were modelled with negative binomial distributions NB(*r*, 1 − *p*), parameterized by *r* and *p*. For *r* > 1, *q*(*z*_1:*T*_) was represented over an expanded Markov chain, in which each behavioural state was augmented with *r* − 1 substates^60^. We then updated *r* and *p* for both leaving and returning states by fitting negative binomial distributions to state durations in samples from *q*(*z*_1:*T*_). For simplicity, we fixed *r* = 1 during all but the final iteration of the fitting algorithm.

For the average fly model, we fitted a single set of optimal parameters (except for **μ**_0_ and ${\sigma }_{0}^{2}$, which are still trajectory specific) to latent variable distributions calculated using the above procedure from the 28 trajectories with no fewer than 30 returns (Extended Data Fig. 8c,h). We chose a relatively large threshold on the number of returns to provide a high number of data points for determining the memory update parameters.

Average fly model parameters (Extended Data Fig. 8c): *w*_∥_ = 6.299 mm s^−1^; *w*_⊥_ = 1.695 mm s^−1^; entry memory (*a* = 0.727, *b* = 0.437 mm s^−1^) and exit memory (*a* = 0.956, *b* = 0.482 mm/s); return after entry (*r* = 2, *p* = 0.505) and leave after exit (*r* = 1, *p* = 0.81); returning state (*c* = 0.733, *σ* = 3.23 mm s^−1^) and leaving state (*c* = 0.6, *σ* = 3.255 mm s^−1^).

#### Comparison with data

We remove odour changes lasting only one or two time steps from simulated trajectories before comparing to fly data, which matches the preprocessing procedure for fly data that removed odour changes lasting less than 0.5 s.

### Dynamic plume analysis

#### Plume video

We used a previously recorded 3,600-frame video (4 min long) of a surface odour plume^3,17^ in two forms: one in the original scale, with the plume video sampled at 15 Hz and a spatial resolution of 0.74 mm per pixel, and another in an expanded scale stretched fivefold in both space and time, simulating an expanded video sampled at 3 Hz with a spatial resolution of 3.7 mm per pixel. Because the edge tracking model depends on binary odour states, we binarized the odour signal with a threshold of 0.1 to assure sufficient odour encounters while preserving the turbulent properties at the downwind end of the plume, allowing us to explore how the model performs with different degrees of turbulence (Extended Data Fig. 13b).

#### Model simulations and threshold for success

We used the average fly model to simulate 2,000 trajectories for each dynamic plume type. For the unscaled plume, we set the simulation time step to 1/15 s (the frame rate of the video) and converted model parameters accordingly. We initialized trajectories uniformly within a 200 mm × 100-mm rectangular region spanning 100–300 mm downwind of the source and −60 to 40 mm in the crosswind direction, to assess how tracking success depends on the initial position within the plume. A trial was considered a success if the minimum distance of the simulated trajectory to the source was less than 20 mm. For the scaled plume, we set the simulation time step to 0.2 s, as in edge-tracking simulations, and we used the video frame closest in time at each step. We initialized trajectories uniformly within a 1,000 mm × 500-mm rectangular region spanning 500–1,500 mm downwind of the source and −300 to 200 mm in the crosswind direction. For the scaled plume, a trial was considered a success if the minimum distance to the source was less than 50 mm.

#### Effective entries and exits in dynamic plumes

Effective entry and exit events during simulation were defined over a 1-s interval (entry: 0.5 s odour off, 0.5 s odour on; exit: 0.5 s odour on, 0.5 s odour off), to be consistent with memory updating in edge-crossing events (plotted in Fig. 5b,e and Extended Data Fig. 13g). When comparing models with and without entry memory, we included only trajectories with at least one effective entry. For the unscaled plume, we further excluded trajectories for which the first effective entry was within a 20-mm alongwind distance from the source, yielding 1,593 out of 2,000 trials. For the scaled plume, we further excluded trajectories for which the first effective entry was within a 50 mm alongwind distance from the source, yielding 1,850 out of 2,000 trials.

#### Entry-memory crosswind predictivity

We denote ${\hat{{\bf{m}}}}_{\mathrm{entry}}={({m}_{\perp },{m}_{\parallel })}^{T}$ as the entry-memory vector normalized to unit length and $\hat{{\bf{v}}}={({v}_{\perp },{v}_{\parallel })}^{T}$ as the entry-velocity unit vector. Crosswind predictivity is calculated as the product of *m*_⊥_ and *v*_⊥_ at the time of each entry.

### Statistics and reproducibility

Data were processed and analysed using custom scripts written in Python and MATLAB. No statistical methods were used to predetermine sample sizes; sample sizes were selected on the basis of common practice in the field and were comparable with those used in previous studies. No randomization of experimental sessions and no blinding to experimental conditions were used during data collection or analysis. The principal behavioural finding—that flies track odour plumes by repeatedly exiting and returning to the plume boundary (edge tracking)—was highly reproducible across flies, experimental paradigms and experimenters. Unless otherwise stated, all statistical comparisons were performed using two-tailed non-parametric tests. Statistical details, including sample sizes, statistical tests and exact *P* values, are provided in the corresponding figure legends or Supplementary Table 1.

### Reporting summary

Further information on research design is available in the Nature Portfolio Reporting Summary linked to this article.

## Online content

Any methods, additional references, Nature Portfolio reporting summaries, source data, extended data, supplementary information, acknowledgements, peer review information; details of author contributions and competing interests; and statements of data and code availability are available at https://doi.org/10.1038/s41586-026-10827-7.

## Supplementary information

Supplementary Tables Supplementary Tables 1 and 2

Reporting Summary

Peer Review file

Supplementary Video 1 Video showing a fly navigating within the closed-loop olfactory paradigm. The fly walks on an air-supported ball with a tube providing a constant airstream yoked to its angular heading. Odour is introduced into the airstream contingent on the fly’s virtual position, creating precisely defined chemical landscapes for navigation.

Supplementary Video 2 Video showing a fly tracking the edge of a vertical plume. Left: rear view of the fly walking on an air-supported ball, with the air/odour tube positioned in front. Middle: enlarged view of the fly’s trajectory as it crosses the boundary of the fictive odour plume. Right: fly trajectory mapped onto a 1-metre gradient plume.

Supplementary Video 3 Video showing the trajectory of a fly tracking a jumping plume. The boundaries of the plume are shown as white lines, which jump 20 mm in the opposite direction of the fly’s heading each time the fly exits. Fly trajectory shown in grey/magenta.

Supplementary Video 4 Video showing the trajectory of a fly tracking a naturalistic plume. The fading trail shows the previous 30-s trajectory of the fly, with red indicating times inside the odour and black indicating times outside the plume. Trajectory scaled to represent around 2× the width of a fly for visualization purposes. Replayed at 10× speed.

## Data Availability

Data supporting the findings of this study are available via Zenodo at https://doi.org/10.5281/zenodo.20751812 (ref. ^61^). Additional requests will be fulfilled by the corresponding author.

## Code availability

Code for the switching state-space model is available at https://github.com/rutalaboratory/Edge-Tracking-Model.
