# Supplementary material for: A vector-based strategy for olfactory navigation in Drosophila
Source: Nature. 2026 Jul 22;657(8131):443–54. doi: 10.1038/s41586-026-10827-7 (PMC13558081; doi:10.1038/s41586-026-10827-7)
Supplement: Supplementary file 1 [file 41586_2026_10827_MOESM1_ESM.pdf]

nature portfolio

https://doi.org/10.1038/s41586-026-10827-7

Supplementary information

A vector-based strategy for olfactory navigation in *Drosophila*

In the format provided by the authors and unedited

Supplementary Information Guide:

Supplementary Video 1: Video showing a fly navigating within the closed loop olfactory paradigm. The fly walks on an air-supported ball with a tube providing a constant airstream yoked to its angular heading. Odor is introduced into the airstream contingent on the fly's virtual position, creating precisely defined chemical landscapes for navigation.

Supplementary Video 2: Video showing a fly tracking the edge of a vertical plume. Left: Rear view of the fly walking on an air-supported ball, with the air/odor tube positioned in front. Middle: Enlarged view of the fly's trajectory as it crosses the boundary of the fictive odor plume. Right: Fly trajectory mapped onto a 1-meter gradient plume.

Supplementary Video 3: Video showing the trajectory of a fly tracking a jumping plume. The boundaries of the plume are shown as white lines, which jump 20 mm in the opposite direction of the fly's heading each time the fly exits. Fly trajectory shown in grey/magenta.

Supplementary Video 4: Video showing the trajectory of a fly tracking a naturalistic plume. The fading trail shows the prior 30 sec trajectory of the fly with red indicating times inside odor and black outside the plume. Trajectory scaled to represent ~2x the width of a fly for visualization purposes. Replayed at 10x speed.

Supplementary Table 1 Statistics and Sample Sizes

Supplementary Table 2 Pearson correlation between binned histogram values from model simulations and fly data

Table S1
Statistics and Sample Sizes

Figure	Group	Sample size	Statistical tests	p value	Additional notes
**1d**	in air, in odor for flies tracking a vertical plume	n = 40 flies	Paired t-test	p=0.00096	plume distance
				p=2.60e-06	orthogonal distance
			Wilcoxon signed rank	p=4.60e-10	speed
				p=4.60e-10	plume speed
				p=8.85e-06	orthogonal speed
**1f**	outbound, inbound	n = 40 flies	Wilcoxon signed rank	p=1.82e-12	angle
			Paired t-test	p=0.00014	distance
**2j**	traveling direction of flies after plume disappears	n=12 flies (0°)<br>n=11 flies (45°)<br>n=6 flies (90°)	One way Watson-Williams with Bonferroni correction	p= 2.0e-09	0° vs 90 °
				p= 5.3e-08	0° vs. 45°
				p=0.05	45° vs. 90°
**3f**	EPG >GtACR1<br>returns per meter	n = 12 flies	Wilcoxon signed rank	p=0.0049	660 nm LED on vs. LED off
	UAS>GtACR1<br>returns per meter	n = 6 flies	Wilcoxon signed rank	p=0.5625	
	EPG >GtACR1<br>|distance upwind|	n = 12 flies	Paired t-test	p=0.0087	
	UAS>GtACR1<br>|distance upwind|	n = 6 flies	Wilcoxon signed rank	p=0.7108	
**4h**	flies/model with or without operant training	n = 15 flies	Kruskal-Wallis H-test	p = 0.00463	plume re-encounters plume with or without operant training
		n = 31 simulations	Kruskal-Wallis H-test	p = 1.07e-05	
**5f**	Entry memory for flies along dynamic	n=12 flies	Mann-Whitney U rank test	height (1429-878), p=0.655<br>height (1429-327), p=0.007<br>height (878-327), p=0.006	
**5h**	Outside bout path length for average fly model with or without entry memory	n=1850 simulations	Welch's t-test	y=1421.33, p=0.5792<br>y=1260.00, p=0.0206<br>y=1098.67, p=0.0245<br>y=937.34, p=0.0088<br>y=776.01, p=4.8393e-09<br>y=614.68, p=1.9503e-09<br>y=453.34, p=1.8777e-13<br>y=292.01, p=3.5233e-13<br>y=130.68, p=2.8275e-12	p value for each alongwind location
	Success rate for average fly model with or without entry memory	n=1850 simulations	Two-proportion z-test	y=1418.35, p=2.430e-05<br>y=1258.73, p=1.7881e-10<br>y=1104.40, p=1.259e-08<br>y=944.69, p=1.1480e-07<br>y=782.35, p=5.8053e-08<br>y=617.732, p=1.7660e-13<br>y=476.317, p=0.00031<br>y=300.492, p=1.73220e-05<br>y=130.597, p=0.03932	p value for each alongwind location bin
**6b**	FC2 >GtACR1<br>returns per meter	n = 14 flies	Wilcoxon signed rank	p = 0.009	530 nm LED on vs. LED off
	UAS>GtACR1<br>returns per meter	n = 9 flies	Wilcoxon signed rank	p = 0.461	
	FC2 > GtACR1<br>|distance upwind|	n = 14 flies	Wilcoxon signed rank	p = 0.005	
	UAS>GtACR1<br>|distance upwind|	n = 9 flies	Wilcoxon signed rank	p = 0.547	
**ED 2b**	tracking a vertical plume of constant, increasing or decreasing odor concentration	n=12 flies	Kruskal–Wallis with Dunn's multiple-comparisons test	p>0.05	inside speed, outside speed, inside plume distance, outside plume distance, inside pathlength, outside pathlength,

					inside time, outside time
**ED 2d**	tracking a vertical plume of constant, increasing or decreasing odor concentration	n=12 flies	Wilcoxon signed rank	p>0.05	inside speed, outside speed, inside time, outside time, inside pathlength, outside pathlength
**ED 3c**	sharp, graded plume<br>inside plume	n=10 flies	Wilcoxon signed rank	p = 0.06445	speed
				p = 1.953e-3	upwind distance
				p = 1.953e-3	pathlength
				p = 1.953e-3	time
	sharp, graded plume<br>outside plume	n=10 flies	Wilcoxon signed rank	p = 0.8457	speed
				p = 0.8457	upwind distance
				p = 0.8457	pathlength
				p = 0.7695	time
**ED 4e**	Orco>Chrimson flies<br>wind, no wind	n=14 flies (no wind)<br>n=15 flies (wind)	Mann-Whitney U	p = 5.097e-6	upwind distance
**ED 4f**	Orco>Chrimson flies<br>inside, outside fictive plume (with wind)	n = 15 flies	Wilcoxon signed rank	p = 0.4887	time
				p = 0.1514	plume distance
				p = 0.4887	orthogonal distance
				p = 0.1205	speed
				p = 0.03015	plume speed
				p = 0.5245	orthogonal speed
**ED 4g**	optogenetic, odor plume<br>inside plume	n = 40 flies (Canton-S, acv plume)<br><br>n = 15 flies (Orco>Chr, fictive plume)	Mann-Whitney U	p = 0.7287	time
				p = 2.344e-3	plume distance
				p = 0.02205	orthogonal distance
				p = 0.01784	speed
				p = 0.0402	plume speed
				p = 0.07282	orthogonal speed
	optogenetic, odor plume<br>outside plume	n = 40 flies (Canton-S, acv plume)<br><br>n = 15 flies (Orco>Chr, fictive plume)	Two-sided Mann-Whitney U	p = 0.03831	time
				p = 2.053e-3	plume distance
				p = 0.1105	orthogonal distance
				p = 0.6556	speed
				p = 0.5587	plume speed
				p = 0.8506	orthogonal speed
**ED 5e**	pathlength for outside bouts<br>real flies (1)<br>simulated trajectories, *a*=0 (2)<br>simulated trajectories, a=1 (3)	n=755 outside trajectories from 40 flies or 755 simulated trajectories	Kruskal–Wallis with Dunn's multiple-comparisons test	(1, 2) p=3.65107e-7<br>(1, 3) p=2.131035e-4<br>(2, 3) p=0.562384	pathlength for outside bouts
**ED 6b**	flies tracking plumes of different orientations	n = 40 flies (0°)<br>n = 21 flies (45°)<br>n = 20 flies (90°)<br>n = 24 flies (jump)	Kruskal–Wallis with Dunn's multiple-comparisons test	(0, 45) p=0.000150<br>(0, 90) p=0.000008<br>(0, jump) p=0.992539<br>(45, 90) p=1.00000<br>(45,jump) p=0.055501<br>(90, jump) p=0.003577	time of inside bouts
				(0, 45) p= 0.173647<br>(0, 90) p=1.00000<br>(0, jump) p= 0.002681<br>(45, 90) p=1.00000<br>(45,jump) p=0.000003 (90, jump) p= 0.002253	time of outside bouts
**ED 6c**	tracking vertical, 45-degree, horizontal and jumping plume	n = 30 flies (0°)<br>n=24 flies (45°)<br>n = 27 flies (90°)	Kruskal–Wallis with Dunn's multiple-comparisons test	(0,45) p = >0.9999<br>(0,90) p = 0.2164<br>(0, jump) p = 0210<br>(45,90) p = 0.7296<br>(45, jump) p = 0.0084	Encounters per minute

		n = 20 flies (jump)		(90, jump) p = <0.0001	
				(0,45) p = 0.1042<br>(0,90) p = 0.4143<br>(0, jump) = 0.0009<br>(45,90) p = >0.9999<br>(45, jump) p = <0.0001<br>(90, jump) p = <0.0001	Encounters per meter
**ED 6e**	in air, in odor<br>flies tracking a vertical plume	n=40 flies	Paired t-test	1.66e-06	time
			Paired t-test	1.39e-06	path length
			Paired t-test	0.00096	plume distance
			Paired t-test	2.60e-06	orthogonal distance
			Wilcoxon signed rank	4.60e-10	speed
			Wilcoxon signed rank	1.82e-11	plume speed
			Wilcoxon signed rank	8.85e-06	orthogonal speed
**ED 6f**	in air, in odor<br>flies tracking a 45-degree plume	n=21 flies	Paired t-test	8.02e-06	time
			Paired t-test	2.38e-05	path length
			Wilcoxon signed rank	0.0175467	plume distance
			Paired t-test	0.002057	orthogonal distance
			Wilcoxon signed rank	2.86e-06	speed
			Wilcoxon signed rank	4.77e-06	plume speed
			Wilcoxon signed rank	4.77e-06	orthogonal speed
**ED 6g**	in air, in odor<br>flies tracking a 90-degree plume	n=13 flies	Paired t-test	0.000724	time
			Wilcoxon signed rank	0.000244	path length
			Wilcoxon signed rank	0.000244	plume distance
			Wilcoxon signed rank	0.000244	orthogonal distance
			Wilcoxon signed rank	0.00244	speed
			Wilcoxon signed rank	0.000732	plume speed
			Wilcoxon signed rank	0.002441	orthogonal speed
**ED 6h**	in air, in odor<br>flies tracking a jumping plume	n=21 flies	Paired t-test	1.67e-07	time
			Paired t-test	2.48e-05	path length
			Paired t-test	0.882320	plume distance
			Wilcoxon signed rank	0.000908	orthogonal distance
			Wilcoxon signed rank	1.19e-07	speed
			Wilcoxon signed rank	1.19e-07	plume speed
			Wilcoxon signed rank	0.000568	orthogonal speed
**ED 7a**	time of sequential outside bouts for different plume types	n = 28 flies (0°)<br>n = 24 flies (45°)<br>n = 20 flies (90°)<br>n = 24 flies (jump)	Mann–Whitney U test	Vertical (0°):<br>(1, 2), p=0.33208<br>(1, 5), p=0.46519<br>45°:<br>(1, 2), p=0.02060<br>(1, 5), p=0.05442<br>90°:<br>(1, 2), p=0.00102<br>(1, 5), p=0.00060	p values for comparing (bout 1 vs bout 2), or (bout 1, bout 5)

				Jumping:<br>(1, 2), p=0.03282<br>(1, 5), p=0.02284	
**ED 7a**	Path length of sequential outside bouts for different plume types	n = 28 flies (0°)<br>n = 24 flies (45°)<br>n = 20 flies (90°)<br>n = 24 flies (jump)	Mann–Whitney rank test	Vertical (0°):<br>(1, 2), p=0.32342<br>(1, 5), p=0.65437<br>45°:<br>(1, 2), p=0.01805<br>(1, 5), p=0.03955<br>90°:<br>(1, 2), p=0.00039<br>(1, 5), p=0.00070<br>Jumping:<br>(1, 2), p=0.03636<br>(1, 5), p=0.01257	p values for comparing (bout 1 vs bout 2), or (bout 1, bout 5)
**ED 10e**	average fly model (1)<br>model w/ no entry memory (2)<br>model w/ constant exit memory (3)<br>model w/ resampled exit memory (4)<br>model w/ upwind exit memory (5)	n= 75 simulations for each model	Kruskal–Wallis with Dunn's multiple-comparisons test	(1, 2) p=7.931e-08<br>(1, 3) p=0.9079<br>(1, 4) p=0.1889<br>(1, 5) p=0.7689<br>(2, 3) p=4.150e-08<br>(2, 4) p=5.014e-05<br>(2, 5) p=3.879e-07<br>(3, 4) p=0.1529<br>(3, 5) p=0.6822<br>(4, 5) p=0.3078	All plumes
**ED 10e**	average fly model (1)<br>model w/ no entry memory (2)<br>model w/ constant exit memory (3)<br>model w/ resampled exit memory (4)<br>model w/ upwind exit memory (5) (5)	n= 25 simulations for each model	Kruskal–Wallis with Dunn's multiple-comparisons test	(1, 2) p=3.753e-04<br>(1, 3) p=0.6436<br>(1, 4) p=0.09204<br>(1, 5) p=0.4488<br>(2, 3) p=5.831e-05<br>(2, 4) p=0.06119<br>(2, 5) p=1.601e-05<br>(3, 4) p=0.03176<br>(3, 5) p=0.7686<br>(4, 5) p=0.0146	vertical plume
**ED 10e**	average fly model (1)<br>model w/ no entry memory (2)<br>model w/ constant exit memory (3)<br>model w/ resampled exit memory (4)<br>model w/ upwind exit memory (5)	n= 25 simulations for each model	Kruskal–Wallis with Dunn's multiple-comparisons test	(1, 2) p=1.291e-04<br>(1, 3) p=0.4462<br>(1, 4) p=0.2289<br>(1, 5) p=0.9829<br>(2, 3) p=4.434e-06<br>(2, 4) p=4.870e-07<br>(2, 5) p=1.408e-04<br>(3, 4) p=0.6589<br>(3, 5) p=0.4335<br>(4, 5) p=0.2207	45-degree plume
**ED 10e**	average fly model (1)<br>model w/ no entry memory (2)<br>model w/ constant exit memory (3)<br>model w/ resampled exit memory (4)<br>model w/ upwind exit memory (5)	n= 25 simulations for each model	Kruskal–Wallis with Dunn's multiple-comparisons test	(1, 2) p=6.733e-03<br>(1, 3) p=0.7282<br>(1, 4) p=1.246e-02<br>(1, 5) p=4.644e-02<br>(2, 3) p=2.234e-03<br>(2, 4) p=0.8330<br>(2, 5) p=0.4.725<br>(3, 4) p=4.421e-03<br>(3, 5) p=1.934e-02<br>(4, 5) p=0.6117	90-degree plume
**ED 10g**	time of sequential outside bouts for simulated trajectories	n = 25 simulations (0°)<br>n = 25 simulations (45°)<br>n = 25 simulations (90°)<br>n = 25 simulations (jump)	Mann–Whitney rank test	Vertical (0°):<br>(1, 2), p=0.21070<br>(1, 5), p=0.71385<br>45°:<br>(1, 2), p=0.43845<br>(1, 5), p=0.36595<br>90°:<br>(1, 2), p=0.41610<br>(1, 5), p=0.04797<br>Jumping:<br>(1, 2), p=0.38644<br>(1, 5), p=0.19055	p values for comparing (bout 1 vs bout 2), or (bout 1, bout 5)
**ED 10g**	Path length of sequential outside bouts for simulated trajectories	n = 25 simulations (0°)	Mann–Whitney rank test	Vertical (0°):<br>(1, 2), p=0.21562	p values for comparing (bout 1

		n = 25 simulations (45°)<br>n = 25 simulations (90°)<br>n = 25 simulations (jump)		(1, 5), p=0.70464<br>45°:<br>(1, 2), p=0.47404<br>(1, 5), p=0.24528<br>90°:<br>(1, 2), p=0.33372<br>(1, 5), p=0.04559<br>Jumping:<br>(1, 2), p=0.34814<br>(1, 5), p=0.25489	vs bout 2), or (bout 1, bout 5)
**ED 11b**	flies with or without operant training<br>pre (1)<br>no training (2)<br>same training, 5X (3)<br>opposite training, 1X (4)<br>opposite training, 5x (5)	n=62 flies (initial segment)<br>n=17 flies (same training)<br>n=15 flies (no training)<br>n=15 flies (1X opposite training)<br>n=15 flies (5X opposite training)	Kruskal–Wallis with Dunn's multiple-comparisons test	(1, 2) p=1.2915e-08<br>(1, 3) p=1.1801e-09<br>(1, 4) p=2.4689e-03<br>(1, 5) p=2.9562e-03<br>(2, 3) p=0.93497<br>(2, 4) p=0.036068<br>(2, 5) p=0.032403<br>(3, 4) p=0.024944<br>(3, 5) p=0.022205<br>(4, 5) p=0.96551	fly trajectories re-encounters per meter
**ED 11b**	model with or without operant training<br>pre (1)<br>no training (2)<br>same training, 5X (3)<br>opposite training, 1X (4)<br>oppostie training, 5x (5)	n=31 simulations for each condition	Kruskal–Wallis with Dunn's multiple-comparisons test	(1, 2) p=1.0397e-06<br>(1, 3) p=8.4910e-07<br>(1, 4) p=0.58465<br>(1, 5) p=0.51096<br>(2, 3) p=0.96829<br>(2, 4) p=1.4420e-05<br>(2, 5) p=3.0019e-08<br>(3, 4) p=1.2026e-05<br>(3, 5) p=2.3903e-08<br>(4, 5) p=0.22861	model trajectories re-encounters per meter
**ED 13j**	Outside bout path length for average fly model with or without entry memory	n=1593 simulations	Welch's t-test	y=284.80, p=0.92955<br>y=253.66, p=0.23023<br>y=222.50, p=0.46208<br>y=191.34, p=0.79200<br>y=160.19, p=0.17362<br>y=129.04, p=0.61093<br>y=97.89, p=0.02869<br>y=66.73, p=0.07374<br>y=35.58, p=0.01254	P value for each alongwind location bin
	Success rate for average fly model with or without entry memory	n=1593 simulations	Two-proportion z-test	y=285.50, p=0.01147<br>y=252.35, p=0.04257<br>y=222.21, p=0.00179<br>y=190.93, p=0.08857<br>y=160.09, p=0.00012<br>y=129.46, p=0.00256<br>y=99.98, p=0.03737<br>y=67.43, p=0.12873<br>y=35.90, p=0.11251	P value for each alongwind location bin
**ED 14e**	PVA for EPG and FC2 during edge tracking	n= 6 flies	Paired t-test	EPG, p = 0.34<br>FC2, p = 0.0077	EPG and FC2 neurons at exit or entry
	∆F/F EPG and FC2 during edge tracking	n=6 flies	Paired t-test	EPG, p = 0.87<br>FC2, p = 0.67	

Table S2: **Pearson correlation between binned histogram values from model simulations and fly data** (**Extended Data Fig. 9**).

Vertical Plume

Bout	Duration	Plume Distance	Orthog. Distance	Plume Speed	Orthog. Speed
Out of odor	0.974	0.966	0.897	0.894	0.513
In odor	0.982	0.919	0.949	0.879	0.819

45° Plume

Bout	Duration	Plume Distance	Orthog. Distance	Plume Speed	Orthog. Speed
Out of odor	0.902	0.814	0.567	0.551	0.332
In odor	0.927	0.989	0.851	0.976	0.629

90° Plume

Bout	Duration	Plume Distance	Orthog. Distance	Plume Speed	Orthog. Speed
Out of odor	0.907	0.911	0.971	0.846	0.740
In odor	0.876	0.955	0.900	0.968	0.757
