# Supplementary material for: A vector-based strategy for olfactory navigation in Drosophila
Source: Nature. 2026 Jul 22;657(8131):443–54. doi: 10.1038/s41586-026-10827-7 (PMC13558081; doi:10.1038/s41586-026-10827-7)
Supplement: Supplementary file 2 [file 41586_2026_10827_MOESM2_ESM.pdf]

nature portfolio

Corresponding author(s): Vanessa Ruta

Last updated by author(s): 2026/06/11

Reporting Summary

Nature Portfolio wishes to improve the reproducibility of the work that we publish. This form provides structure for consistency and transparency in reporting. For further information on Nature Portfolio policies, see our Editorial Policies and the Editorial Policy Checklist.

Statistics

For all statistical analyses, confirm that the following items are present in the figure legend, table legend, main text, or Methods section.

n/a	Confirmed	
☐	☒	The exact sample size (n) for each experimental group/condition, given as a discrete number and unit of measurement
☐	☒	A statement on whether measurements were taken from distinct samples or whether the same sample was measured repeatedly
☐	☒	The statistical test(s) used AND whether they are one- or two-sided<br>*Only common tests should be described solely by name; describe more complex techniques in the Methods section.*
☒	☐	A description of all covariates tested
☐	☒	A description of any assumptions or corrections, such as tests of normality and adjustment for multiple comparisons
☐	☒	A full description of the statistical parameters including central tendency (e.g. means) or other basic estimates (e.g. regression coefficient) AND variation (e.g. standard deviation) or associated estimates of uncertainty (e.g. confidence intervals)
☐	☒	For null hypothesis testing, the test statistic (e.g. F, t, r) with confidence intervals, effect sizes, degrees of freedom and P value noted<br>*Give P values as exact values whenever suitable.*
☒	☐	For Bayesian analysis, information on the choice of priors and Markov chain Monte Carlo settings
☒	☐	For hierarchical and complex designs, identification of the appropriate level for tests and full reporting of outcomes
☐	☒	Estimates of effect sizes (e.g. Cohen's d, Pearson's r), indicating how they were calculated

Our web collection on statistics for biologists contains articles on many of the points above.

Software and code

Policy information about availability of computer code

Data collection	Behavior of tethered flies was recorded using FicTrac v2.0. Custom scripts in Python (v3.7+) were used to log behavioral variables from FicTrac and synchronize these with hardware signals from a Raspberry Pi 4 to control the closed-loop olfactory system. Two-photon calcium imaging data was acquired on Prairie View software 5.7.64.300 and 5.8.64.800 (Bruker).
Data analysis	Behavioral trajectories were analyzed using Python v3.7+. Functional imaging data (EPG and FC2 neuron recordings) were processed using a combination of Fiji (ImageJ), custom Python scripts, and a custom MATLAB GUI. Images. Simulations based on the random search model or the switching state-space model were implemented in custom Python scripts (https://github.com/rutalaboratory/Edge-Tracking-Model).

For manuscripts utilizing custom algorithms or software that are central to the research but not yet described in published literature, software must be made available to editors and reviewers. We strongly encourage code deposition in a community repository (e.g. GitHub). See the Nature Portfolio guidelines for submitting code & software for further information.

Data

Policy information about availability of data

All manuscripts must include a data availability statement. This statement should provide the following information, where applicable:

- Accession codes, unique identifiers, or web links for publicly available datasets
- A description of any restrictions on data availability
- For clinical datasets or third party data, please ensure that the statement adheres to our policy

All data supporting this study are available upon request.

Research involving human participants, their data, or biological material

Policy information about studies with human participants or human data. See also policy information about sex, gender (identity/presentation), and sexual orientation and race, ethnicity and racism.

Reporting on sex and gender	n/a
Reporting on race, ethnicity, or other socially relevant groupings	n/a
Population characteristics	n/a
Recruitment	n/a
Ethics oversight	n/a

Note that full information on the approval of the study protocol must also be provided in the manuscript.

Field-specific reporting

Please select the one below that is the best fit for your research. If you are not sure, read the appropriate sections before making your selection.

☒ Life sciences ☐ Behavioural & social sciences ☐ Ecological, evolutionary & environmental sciences

For a reference copy of the document with all sections, see nature.com/documents/nr-reporting-summary-flat.pdf

Life sciences study design

All studies must disclose on these points even when the disclosure is negative.

Sample size	Preliminary experiments were used to assess variance. Sample sizes were not predetermined but kept consistent within comparison groups and comparable to other studies and were sufficient to observe robust and reproducible behavioral or neural effects across flies. Experiments were repeated across multiple flies and independent trials to ensure reproducibility.
Data exclusions	Flies that failed to acclimate to walking on the treadmill or did not show a behavioral response to odor (i.e., no upwind movement or increased speed upon odor exposure) were excluded from analysis (<10%). These exclusion criteria were pre-determined.
Replication	All key findings were replicated across multiple flies (typically 6-25), independent trials and independent experimenters. Behavioral assays, imaging results, and model predictions were consistent across replicates. Replication was verified by consistency of edge-tracking behavior and neural response patterns within and across animals.
Randomization	Flies were randomly selected from the population of identically-reared animals of the same genotype and all testing was conducted under identical environmental conditions. Behavioral experiments were performed interleaved with their controls (where relevant). In cases where the same flies were subject to multiple different plume types (e.g. with and without gradients) the order of plume type was randomized.
Blinding	Investigators were not blinded to experimental conditions during data collection, as experimental paradigms and genotypes were readily identifiable. Behavioral and neural measurements were extracted using automated analysis pipelines and predefined criteria, reducing the potential for experimenter bias during analysis.

Reporting for specific materials, systems and methods

We require information from authors about some types of materials, experimental systems and methods used in many studies. Here, indicate whether each material, system or method listed is relevant to your study. If you are not sure if a list item applies to your research, read the appropriate section before selecting a response.

Materials & experimental systems

n/a	Involved in the study
☒	☐ Antibodies
☒	☐ Eukaryotic cell lines
☒	☐ Palaeontology and archaeology
☐	☒ Animals and other organisms
☒	☐ Clinical data
☒	☐ Dual use research of concern
☒	☐ Plants

Methods

n/a	Involved in the study
☒	☐ ChIP-seq
☒	☐ Flow cytometry
☒	☐ MRI-based neuroimaging

Animals and other research organisms

Policy information about studies involving animals; ARRIVE guidelines recommended for reporting animal research, and Sex and Gender in Research

Laboratory animals	This study used female 1-5 day old Drosophila melanogaster. Wild-type strain is Canton-S. Transgenic genotypes used in this study are listed in the Methods.
Wild animals	This study did not use wild animals.
Reporting on sex	Only female flies were used for behavioral and imaging experiments due to their larger size and greater robustness.
Field-collected samples	No field-collected sample were used in this study.
Ethics oversight	Drosophila melanogaster is not subject to institutional animal care and use regulations; therefore, no ethical approval was required for this work.

Note that full information on the approval of the study protocol must also be provided in the manuscript.

Plants

Seed stocks	n/a
Novel plant genotypes	n/a
Authentication	n/a
