# Supplementary material for: A vector-based strategy for olfactory navigation in Drosophila
Source: Nature. 2026 Jul 22;657(8131):443–54. doi: 10.1038/s41586-026-10827-7 (PMC13558081; doi:10.1038/s41586-026-10827-7)
Supplement: Supplementary file 3 [file 41586_2026_10827_MOESM3_ESM.pdf]

nature portfolio

Peer Review File

A vector-based strategy for olfactory navigation in Drosophila

Corresponding Author: Professor Vanessa Ruta

Any redactions in this file are there to maintain patient confidentiality, the confidentiality of unpublished data, or to remove third-party material.

This file contains all reviewer reports in order by version, followed by all author rebuttals in order by version.

Attachments originally included by the reviewers as part of their assessment can be found at the end of this file.

Version 0:

Reviewer comments:

Referee #1

(Remarks to the Author)
Navigating the natural environment towards a goal requires efficient use of sensory cues that can fluctuate over time. Olfactory cues are particularly dynamic as their spatiotemporal structure depends on the characteristics of the wind, making the task especially challenging. This study by Siliciano et al. focuses on olfactory navigation and reports that Drosophila uses two strategies, namely tracking the edge of the odor plume and remembering the past angles with which the animal entered the plume, to execute the task. These strategies can be described under the framework of vector-based computation, where the flies continuously update memories of plume entering angles (vectors). This proposal is novel as previous studies mainly focused on the upwind surge upon odor contact and also did not demonstrate the involvement of memories. The authors provide multiple lines of evidence in support of the proposal by making full use of their olfactory virtual reality functioning in closed loop, where the shape of the odor plume as well as the direction of the wind can be set at will. They show that edge-tracking behavior is robustly observed under odor plumes with various shapes, orientations and even with gaps. The behavior reflects the fly's previous experiences and can be broadly recapitulated with a model incorporating memories about plume entering angles. Edge tracking requires the compass neurons in the central complex. Finally, the authors find using calcium imaging that information about the dynamic goal, the direction to recapture the odor plume, is encoded in FC2 neuron in the fan-shaped body of the central complex.
This work represents an important contribution to the field by establishing "edge tracking" as a plausible strategy that is possibly widely used by insects to navigate the complex olfactory landscape to reach a food source. Although I am very much supportive of the work, I would like to add some comments to hopefully further strengthen the study.

Major comments:
1. The authors examined the impact of suppressing EPG neurons on edge tracking. It is important to also test the impact of FC2 suppression on edge-tracking. Will the flies completely lose the capability to edge track? Or will they still be able to follow the vertical plume, walking only within it? This experiment could further lend support to the role of these neurons in short-term memory storage.
2. To explore the fly's behavior to track the edges of odor plumes, the authors focus on the plumes that are precisely shaped in quadrilaterals with different angles with respect to the wind direction. To investigate the relevance of this behavioral strategy in the wild, it will be informative to level up the complexity of the landscape, trying to mimic the natural plumes as much as possible. Under such conditions, it is expected that flies encounter odors with constantly changing irregular geometries so that the entry and exit angle memories alone might make them wander around excessively, making it difficult for them to advance efficiently toward the food source. Therefore, the fly's behavior in the presence of irregular and dynamic plumes could be tested. What would be the behavioral strategy of flies in this complex situation? Will the switching state model still recapitulate the behavior in this scenario?
3. In experiments where the flies were allowed to track a plume for 10 min before it was removed (described in lines 239-247, Fig. 2i, j), why was this particular time chosen? Was there a correlation between the tracking time and the time it takes for the directional bias to vanish passively? One can ask a similar question in the context of operant training described in Fig. 5. Is it possible to examine the relationship between the number of training and the strength of memory as well as the

time constant of memory decay?
4. I acknowledge the value of proposing a plausible biological mechanism for storing entry angles as attempted in Figure 6a. However, this is a hypothesis not addressed in this study. Therefore, I would suggest removing or moving the figure panel to Extended Data Figs.

Minor comments:
5. I would suggest to discuss the authors' thought on why flies (and also the switch state model) track the upper part (closer to the wind source) of 45-degree and 90-degree plumes. From what it is shown in Fig. 2a-c, 4a and 5b, there is not a single case of tracking the lower edge. Also, most of the edge tracking for these configurations do not stretch as long as the vertical edge tracking as observable in Fig. 2a-c, where the density of trajectories decreases midway especially for the 45-degree plume. Why is this the case?
6. In Fig. 6c, almost immediately after the fly enters the plume, the FC2 phase becomes aligned to the EPG phase. What could be the mechanisms behind this rapid shift? It would at least be appropriate to show the time posterior to plume entry in the same way as it is shown for the time prior to plume entry in Figure 6j.
7. Although lines 41-44 are a good summary of the work, I feel that the use of the words 'dynamic landmark' is confusing, since the main point of the work is that flies do not memorize a point (landmark) in space.
8. Line 491 describes a prior study on insects that can track the surface of plumes. It might be better to mention this in the Introduction. Also, as Jun et al. eLife (2015) describe edge tracking in freely behaving flies, this reference should be discussed.
9. In lines 510-514, the analogy between edge tracking and the ant's navigational strategy is a bit of a stretched. Ants have to memorize several directional vectors associated with visual landmarks, while edge tracking as described in this work, only stores a single vector.
10. Extended Data Fig.1a and 1c are not referred to in the text.
11. Line 400. Fig. 4d does not exist.
12. Line 1104. Singh et al. appears twice in the list (36 and 69).
13. The title of Fig. 1 could describe the biological finding rather than the experimental system.
14. The bottom right figure in Fig. 1g was hard to comprehend. There may be a way to better describe it.
15. Extended Data Fig. 4a. Although the legend describes that "the difference in the timing of odor arrival is < 1 ms, the graph goes beyond 1 ms.
16. Extended Dat Fig. 5d. Although the legend describes "Note that in contrast to real flies, simulations lacking an upwind bias (a=0) never progress along the length of the plume.", I cannot recognize a difference between real flies and simulations.
17. Extended Data Fig. 8. How is the goodness of match between the fly and the model evaluated?
18. Extended Data Fig. 9f. Please explain the label for y axis in more detail (entry memory alongwind (mm/s)). Why is the unit mm/s?
19. Extended Data Fig. 10c. The label c needs to be inserted into the legend.

Referee #2

(Remarks to the Author)
Summary assessment: This paper presents an exceptional study of how fruit flies navigate structured chemical landscapes, revealing a strategy called "edge tracking" that relies on vector-based computations within the central complex. Using an innovative virtual reality paradigm for olfactory navigation, the authors demonstrate that flies track an appetitive odor corridor by following its boundary, alternating between rapid exits from the plume and directed returns to its edge. The work provides strong evidence that flies store and dynamically update memories of the direction required to return to the plume's boundary, and identifies neural correlates of this directional memory in FC2 neurons of the central complex. This groundbreaking study significantly advances our understanding of sensory-guided navigation and constitutes a major contribution to the field.

Key strengths and contributions of the work: The manuscript presents work of outstanding quality addressing a fundamental question related to sensory navigation with strong relevance to the ethology of flies. The virtual reality paradigm developed for this study offers unprecedented control of plume boundaries with the ability to manipulate these boundaries in closed-loop based on the fly's behavior. This new assay enables the study of navigation behavior with a precision and resolution not previously achieved.

The authors have identified edge tracking as an essential behavior for navigating plumes and demonstrate that this strategy relies on memories of the relative direction to the plume's boundary, not its location. The work shows that flies use the plume's boundary as a dynamic landmark, remembering it as a chemical signpost. The stimulation protocols take full advantage of the virtual olfactory environment to test specific hypotheses about plume tracking. The authors propose a series of ingenious experimental variants, including the "jumping plume" paradigm that elegantly demonstrates the role of directional but not positional memory.

The data analysis and modeling create exemplary synergy to formulate and test hypotheses. The switching state space model derived from behavioral data captures the essence of edge tracking behavior and provides insights into the timescales of memory formation and updating. The authors make a solid case for the involvement of the central complex in plume navigation through loss-of-function experiments with EPG neurons. The finding that FC2 neurons signal the direction back to the odor boundary during returns provides an entry point into understanding the neural circuit correlates of directional memory.

Several clever control experiments strengthen the work's conclusions, including Orco>CsChrimson controls to test the role of left-right comparison (Extended Figure 4), tests of longitudinal gradients to rule out their contribution (Extended Figure 3),

sensory replay experiments to demonstrate that edge tracking requires using recent entry and exit angles to update goal direction (Figure 4), and operant training protocols showing that entry angles can be dynamically updated through successive odor encounters (Figure 5).

This manuscript makes a superb contribution to our understanding of insect navigation, revealing that odor tracking is not merely a reflexive process but incorporates sophisticated vector-based computations. The work demonstrates impressive methodological innovation, advanced and meticulous data analysis, and a seamless integration of behavioral experiments with computational modeling. The findings advance our understanding of olfactory navigation in flies while simultaneously offering profound insights into fundamental principles of sensory-guided navigation conserved across diverse species.

While this is already an outstanding manuscript with exceptional experimental design and data analysis, I offer the following suggestions to further enhance the clarity of certain results and strengthen how key conclusions are established:

1. Analysis of traversal gradients: The authors convincingly demonstrate that flies do not follow longitudinal odor gradients in their assay, instead exploiting edge tracking. It would be valuable to examine how flies respond to the presence of a more graded traversal odor gradient. Would they switch from edge tracking to meandering across the center of the trail, or would they still focus on edge tracking? This would address the authors' speculation that edge tracking might be a strategy to avoid sensory adaptation.

2. Correlation between exit and entry angles: While the data are quantified in multiple ways, a relatively straightforward analysis of the relationship between exit angles and subsequent entry angles could be informative. For vertical plumes, are larger exit angles correlated with larger subsequent entry angles? This analysis might reveal whether flies are capable of fine-grained course correction at the level of their (first) turn toward the plume upon its exit.

3. Learning curve for plume encounter efficiency: Figure 5 convincingly demonstrates that entry angles can be updated through just a small number of plume encounters. It would strengthen the manuscript to more thoroughly document how the precision of returns to the plume improves with successive encounters across different plume geometries. While Extended Figure 10a touches on this analysis, a more systematic characterization of this learning trajectory would be valuable. Establishing this behavioral feature earlier in the manuscript would provide important context for the sophisticated manipulations that follow and further support the memory-based nature of edge tracking.

4. Visualization of random search model trajectories: In Figure 1g, the authors discuss results of synthetic trajectories generated with a random-search model, but no example trajectories are shown. Including representative trajectories would help readers better understand the contrast with real fly behavior, strengthening the conclusion that edge tracking does not follow the statistics of a random search process.

5. Integration of plume statistics: The authors propose that tracking the plume envelope rather than wind direction might be advantageous (line 196). This hypothesis could be strengthened by quantitatively relating it to the observed timescales of fly responses and memory updating, compared with statistical properties of natural odor plumes. Incorporating quantitative features from the literature on plume statistics (e.g., Celani and Vergassola, Cardé and Willis) would help establish whether the memory mechanisms observed in this study operate on timescales compatible with the dynamics of natural plumes that flies would encounter in their environment.

6. Comparison of ACV vs. Orco>CsChrimson behavior: Extended Figure 4 suggests that flies undergoing Orco>CsChrimson stimulation may spend more time inside the virtual odor corridor than flies experiencing stimulation with real vinegar (ACV). A more direct comparison of the probability distributions of positions would help clarify whether the edge tracking behavior observed with ACV generalizes to other odor stimuli.

7. Response to wind direction changes independent of movement: An interesting extension would be to test how flies respond when the wind direction changes independently of their movement. This would help address how flies distinguish changes due to their own actions from those due to external factors, a key challenge in natural environments with stochastic airflows. While this may exceed the scope of the current work, it would connect to the authors' speculation about "more reflexive strategies" when odor encounters are truly stochastic (lines 529-530).

8. Evolutionary context: While the discussion of evolutionary conservation of navigational toolkits is insightful, there remains a gap between what is demonstrated and what is speculated. The authors might consider tempering some of these assertions or more clearly distinguishing between established findings and hypotheses.

Minor points: Extended Figure 10 appears to be missing the label of panel (c) in its caption. I found no other typos in the manuscript and wish to commend the authors for the exceptional quality of the manuscript, the clarity and neatness of the figures, and the thoroughness of the methodology.

Referee #3

(Remarks to the Author)
This paper provides novel and convincing evidence that the tracking of chemical plumes by Drosophila involves memory, encoded in the central complex, of the direction (relative to the wind direction) of movement of the animal when crossing the edge of the plume. This enables an edge following strategy, including extended periods outside the plume, towards the source. This strategy is suggested to exploit the saliency of the sharp change in odour intensity at the edge, rather than

trying to detect and follow shallow or non-existent gradients in plumes, and to be more efficient that the surge-and-cast strategy previously thought to explain insect plume following.

My main concern with the work, as presented, is that the authors make very free, and in my view rather inaccurate, use of navigational terminology, such as 'landmark' and 'vector' in describing their findings. Indeed the mechanism proposed seems to be a form of menotaxis: the animal remembers a desired heading direction (the direction it was travelling when odour was encountered) relative to an external directional cue (the wind) and can flexibly switch to travelling in this direction to regain contact with the edge of the plume. As such, there are no landmarks ("dynamic" or otherwise) or vectors (i.e. representations of "direction + distance") involved, and comparisons to ant homing and route following are just misleading. In my additional comments I have highlighted specific instances where I find this framing misleading or confusing.

The manuscript is otherwise excellent in most respects, presenting a very thorough exploration of the behaviour and the neural correlates, accompanied by explanatory modelling. However I suggest it could be improved by consideration of the following points:

1) It is not fully clear what is the natural correspondence to simulated plumes that cross the wind direction at angles up to 90 degrees. For example, would the odour source itself need to be moving to produce such a plume? There is brief discussion of this around line 193, referring to transient changes in wind direction. It would be helpful to illustrate what exactly it would mean to 'track the envelope of the plume rather than the wind direction'.

2) It is really not clear to me what the replay experiments are meant to prove (fig 4). It seems self-evident that (line 321) "odor encounters uncoupled" from the animal's movement through space will not produce coherent spatial behaviour, other than perhaps evoking upwind turning, which is what seems to happen. Given fig 2 already shows flies "maintain a directional bias based on their past plume encounters" (line 341), the replay results at most seem to show one or two inconsistent encounters with odour quickly erode this tendency. This is more convincingly shown in the later operant experiments (fig 5) so I think this whole section and figure could be dropped (or made supplementary) simplifying the paper.

3) My impression of the model results is that it is not necessary to assume the fly has two memories (entry and exit angle) but that the exit could be explained by the fly consistently turning into and then slightly past the wind direction when it is in the plume. The FC2 activity results also seem to indicate only setting of a goal direction towards the plume. This possibility is briefly addressed in the discussion (lines 559-562) but as a single memory would be a more parsimonious account, which is still consistent with the data, I think it should be given more weight.

Additional comments:

Line 1: The first sentence of the abstract seems both disputable (odors are not essential for navigation, and odors might guide behaviour without requiring navigation) and unnecessary.

Line 13 and throughout: I am not sure what is meant by a 'dynamic landmark' and it seems a confusing term for understanding how the plume boundary is used.

Line 45, if the memory only indicates a direction, and not a distance, then it might be better not to so prominently feature the term 'vector' throughout the paper (including the title) as 'vector' strictly implies direction and distance (angle and length). Although the term 'vector' is often used loosely in biology to refer only to direction, I think it is crucial to clear understanding of the neural mechanisms of navigation to maintain a distinction between the animal having directional guidance only vs. an intended travel vector with an explicit endpoint.

Line 55-60: I assume that the validation of the chemical landscape produced by the system is provided in previous papers (although the references 15,16 do not have obviously relevant titles). A more explicit reference to how the system was validated (i.e. measured to be producing the intended odor landscape) would be helpful.

Line 60-62: Is it certain that the fly has no other sensory cues for orientation in this paradigm? Presumably it still has proprioceptive cues to its own rotation, for example. I appreciate that the wind cue is probably sufficiently dominant for the purposes of the experiment, but other possible cues should at least be mentioned.

Line 90: a 95% confidence interval of 1.91 to 2.15 seconds spent in the plume seems surprisingly tight, as though flies are performing a very fixed response (consistent with the possibility above that they do not need a memory to direct their exit of the plume. Perhaps this could be characterised further, maybe excluding the 4% of times the flies crossed to the other side of the plume?

Line 311: I agree these results show the flies efficiency in tracking the plume is influenced by EPGs, but do the EPG-deficient flies not still exhibit a form of (less efficient) edge tracking?

Line 315: Is it essential that flies track both entry and exit angles? The evidence provided here (lines 334-335) seem to suggest that odor onset causes steering upwind, not requiring memory, whereas the behaviour after odor offset (in this case a downwind bias) cannot be explained without memory.

Line 492-3 I do not think it is appropriate to describe memory of a heading direction as 'an olfactory landmark'.

Line 500-501 I think it is extremely misleading to describe this as a “vector based strategy similar to what central place foragers... use to home to their nests”. It is absolutely crucial for homing to integrate the directional and distance information in travel vectors. As the following sentences makes clear, there is no evidence that vectors are involved or required for plume edge-tracking, only directions.

Line 511-512 Work on ant navigation over the past decades has cast doubt on the ‘local vector’ account of route following, as it is possible to use rotational match of visual snapshots to recover directions without requiring any association to a vector. See e.g. Collett, M., Chittka, L., & Collett, T. S. (2013). Spatial memory in insect navigation. Current Biology, 23(17), R789-R800. There is also a big difference between ‘serially sampling sensory landmarks’ in the sense of ants storing multiple snapshots (indicating fixed locations along a route) and in the sense of the fly updating a single estimate of the desired direction to re-enter the plume – these are simply not comparable in my view.

Overall the figures are very dense and the authors might want to consider removing some of the subfigures (or moving them to supplementary material). Some specific comments:

Fig 1d) is extremely hard to read (even magnified on screen and it is not clear it is necessary. 1g is not very intuitive, and overall, I think the demonstration that random path segments do not reproduce the edge following could be kept wholly as supplementary, as it is not a surprising finding, although useful as a basic check.

2i and j, any comment on the lack of difference for 45 and 90 degrees after plume is gone? Could this be indicative of a simpler strategy – setting a goal either 90 degrees or 180 degrees to wind direction? Could it be explored in the model whether a very approximate goal is sufficient?

Fig 4, See my comments above, it is also quite hard to understand what is plotted in these figures, and seems somwhat pointless to plot ‘entry direction’ for flies that are experiencing ‘entry’ (odour onset) uncoupled to their action.

Fig 5, how meaningful are stats when the model has been tuned to fit data? 5c, is there a difference here between simulation and flies, with flies more efficient, at least for longer bouts? 5g seems overly complex, making the same point in three different plots. The model seems to show very minor shift for one encounter (5g), is this really enough to make such a difference in success after one training trial (5h)?

Extended data 2, seems large variability across flies? Any comment?

Extended data 1 and 4, the optogenetic introduces no onset/offset gradient? How does this affect behaviour, and could it be simulated?

Version 1:

Reviewer comments:

Referee #1

(Remarks to the Author)
The authors have addressed all the issues raised by this reviewer.

Referee #2

(Remarks to the Author)
This manuscript addresses a question of broad significance for our understanding of sensory-guided navigation in Drosophila. The authors have responded to my previous suggestions with care and thoroughness. All the points I raised in my initial report were seriously considered in the revision. Among the new additions, I found particularly exciting the data in Extended Fig. 3 showing the elegant observation that flies accumulate in the region where the slope of the lateral concentration gradient is maximum. This result is consistent with the established sensitivity of Drosophila olfactory neurons to concentration derivatives. The inclusion of representative simulated trajectories in Extended Fig. 5d is helpful for appreciating the contrast with real fly behavior. The complementary quantification of behavior in real (ACV) and optogenetic olfactory corridors is also a valuable addition. In response to Reviewer 1, the new experimental evidence supporting the role of FC2 neurons shown in Fig. 6 is solid and strengthens the case for the necessity of these neurons in edge tracking, which complements the EPG silencing results that were already compelling in the initial submission.

Analysis of edge tracking in naturalistic plumes: The authors now test their model in plume structures displaying heterogeneous conditions of turbulence. These simulations highlight the role of entry memory in augmenting navigation

success. Using replay of plumes published in past work is a clever strategy. I understand the decision to spatially scale up the dimensions of the plume. Slowing down the playback is indeed a reasonable approach to preserve the overall velocity of odor fluctuations. However, there is no guarantee that the geometric properties of the enlarged plume and the spatiotemporal information a fly collects while moving through it will be comparable to those of the original plume. As the authors themselves note, the spatial and temporal statistics of naturalistic plumes depend on the fluid dynamics of the underlying currents carrying the odor and these are unlikely to be preserved by linear scaling. The reason I raise this limitation is that the authors show virtually no behavior of real flies in the scaled-up plume. While Reviewer Figure 1 suggests that a small number of flies (3) could navigate the plume structure toward its source, more animals would need to be tested to establish this result with confidence. Given that this analysis was elevated to a main figure, I would have expected a more solid comparison between model simulations and the behavior of real flies. Specifically, does the multilayered nature of the navigation strategy deployed by real flies show discrepancies with model predictions? Were real flies generally effective at tracking the scaled-up plume? These questions remain unanswered in the revised manuscript. As a result, the conclusion that flies may deploy multiple navigational strategies depending on plume statistics is left as a hypothesis to be tested in future work. While this is a little unsatisfying, I understand why a complete experimental analysis would go beyond the scope of the current manuscript.

With the caveat noted above regarding the naturalistic plume analysis, I enthusiastically recommend acceptance of this manuscript. As was clear in my initial report, I was highly impressed by the quality of the work and the depth of the questions it addresses. I remain equally impressed by the value and impact of this study after reviewing the revised manuscript. I expect it will be of broad interest to both the navigation and sensory neuroscience communities.

Referee #3

(Remarks to the Author)
I thank the authors for their very thorough consideration of and response to the reviews. The additions and clarifications have strengthened an already excellent paper and I have only a few minor points for consideration:

Extended data figure 1 caption, has b) twice, the second one should be e)

Fig 2 i & j, in the text these results show “flies continued to walk in the same direction they had previously used to return to the plume” for "10s of seconds". It seems apparent in these plots that at 10 seconds, the flies are indeed walking very close to the same direction, but after that they start to diverge (e.g. the 45 degree flies walk closer to the expected 90 degree direction than the 90 degree flies by 30s, although still in the same general direction). Maybe this could be commented on, as indicative of possible memory decay rate?

Finally, I feel the authors could actually strengthen the point made in this paper if they did not, from the start, use 'navigation' as a broad term inclusive of any type of goal-directed behaviour (including reflexive steering), and instead made the distinction that 'navigation' requires “movement towards a desired goal that cannot be continuously sensed” - as they demonstrate occurs here for the fly. It seems counterproductive to dilute terminology that, if used more selectively, can usefully distinguish certain types of behaviour from others based on the computational demands required.

Barbara Webb

Open Access This Peer Review File is licensed under a Creative Commons Attribution 4.0 International License, which permits use, sharing, adaptation, distribution and reproduction in any medium or format, as long as you give appropriate credit to the original author(s) and the source, provide a link to the Creative Commons license, and indicate if changes were made.
In cases where reviewers are anonymous, credit should be given to 'Anonymous Referee' and the source.
The images or other third party material in this Peer Review File are included in the article's Creative Commons license, unless indicated otherwise in a credit line to the material. If material is not included in the article's Creative Commons license and your intended use is not permitted by statutory regulation or exceeds the permitted use, you will need to obtain permission directly from the copyright holder.
To view a copy of this license, visit https://creativecommons.org/licenses/by/4.0/

A vector-based strategy for olfactory navigation in *Drosophila*
[redacted]

Summary of revision for all reviewers
We thank all the reviewers for their constructive and thoughtful comments and their overall enthusiasm for the work. The manuscript has benefitted significantly from their suggestions, through the addition of new data, reanalysis of existing data, revision and reorganization of the figures, as well as clarification of the text throughout the manuscript.

Summary of key changes to manuscript:

1. **We now provide evidence that FC2 neurons are necessary for edge tracking, supporting a critical role in encoding angular goals.** Optogenetic silencing of FC2 neurons significantly impairs edge tracking, paralleling the behavioral phenotypes observed with EPG silencing. These perturbations along with additional analyses of FC2 functional imaging support a role for this population in guiding a goal direction derived from entry-angle memory, particularly during returns to the plume boundary.

2. **We explored how edge tracking may operate in the context of plumes with more naturalistic structures.** To address concerns about the ecological relevance of edge tracking, we expanded our analyses to examine whether this strategy operates in plumes that more closely resemble those encountered in nature. **First**, we examined how flies track odor corridors with smoothly graded lateral profiles characteristic of time-averaged surface plumes. We find that flies still appear to edge track but now ascend along the isocline of steepest concentration change, consistent with the established sensitivity of flies to odor gradients. These results indicate that flies track the edges of plumes even when they lack a sharp boundary. **Second**, we extended our modeling framework to a previously recorded surface plume used in studies of freely walking flies. By quantifying the coherence of available entry angles along the length of the plume and evaluating model performance across distinct turbulence regimes, we show that memory-based edge tracking is advantageous when plume structure is sufficiently stable to provide coherent entry angles. Together, these analyses suggest that memory based navigational strategies, like edge tracking, represent one strategy—but not the only one—that flies adopt, and that different tracking algorithms are likely engaged depending on the coherence and stability of the environment.

Below, we respond point-by-point to the reviewers' comments.

Referee #1 (Remarks to the Author):

Navigating the natural environment towards a goal requires efficient use of sensory cues that can fluctuate over time. Olfactory cues are particularly dynamic as their spatiotemporal structure depends on the characteristics of the wind, making the task especially challenging. This study by Siliciano et al. focuses on olfactory navigation and reports that *Drosophila* uses two strategies, namely tracking the edge of the odor plume and remembering the past angles with which the animal entered the plume, to execute the task.

These strategies can be described under the framework of vector-based computation, where the flies continuously update memories of plume entering angles (vectors). This proposal is novel as previous studies mainly focused on the upwind surge upon odor contact and also did not demonstrate the involvement of memories.

The authors provide multiple lines of evidence in support of the proposal by making full use of their olfactory virtual reality functioning in closed loop, where the shape of the odor plume as well as the direction of the wind

can be set at will. They show that edge-tracking behavior is robustly observed under odor plumes with various shapes, orientations and even with gaps. The behavior reflects the fly's previous experiences and can be broadly recapitulated with a model incorporating memories about plume entering angles. Edge tracking requires the compass neurons in the central complex. Finally, the authors find using calcium imaging that information about the dynamic goal, the direction to recapture the odor plume, is encoded in FC2 neuron in the fan-shaped body of the central complex.

This work represents an important contribution to the field by establishing "edge tracking" as a plausible strategy that is possibly widely used by insects to navigate the complex olfactory landscape to reach a food source. Although I am very much supportive of the work, I would like to add some comments to hopefully further strengthen the study.

We thank the reviewers for their enthusiasm for this work and their thoughtful comments and suggestions.

Major comments:

1. The authors examined the impact of suppressing EPG neurons on edge tracking. It is important to also test the impact of FC2 suppression on edge-tracking. Will the flies completely lose the capability to edge track? Or will they still be able to follow the vertical plume, walking only within it? This experiment could further lend support to the role of these neurons in short-term memory storage.
We have now performed the proposed experiments and compared how flies track when their FC2 neurons are active or optogenetically silenced. As in our EPG-silencing experiments, we used a "jumping plume" in which each time a fly exited the odor corridor, the plume was jumped 20 mm in the direction opposite to the exit, to enhance the likelihood that flies rely on their entry angle memory and don't incidentally return to the plume by chance.

Consistent with the role of FC2 neurons in signaling the fly's angular goal during returns, FC2 silencing significantly impairs the ability of flies to return to the plume's boundary following an excursion, phenocopying the effect of EPG silencing. These results reinforce the proposed role of FC2 neurons as conveying an integrated goal signal. Together with the EPG silencing data, these results also further support the notion that edge tracking relies on vector-based computations mediated by circuits within the central complex, requiring both neurons that store the entry angle memory (e.g. FC2) and neurons that convey the allocentric reference frame in which that angular memory is stored (e.g. EPG).

This new experiment is now included in **Fig. 6b, Extended Data Fig. 14f** in the revised manuscript.

2. To explore the fly's behavior to track the edges of odor plumes, the authors focus on the plumes that are precisely shaped in quadrilaterals with different angles with respect to the wind direction. To investigate the relevance of this behavioral strategy in the wild, it will be informative to level up the complexity of the landscape, trying to mimic the natural plumes as much as possible. Under such conditions, it is expected that flies encounter odors with constantly changing irregular geometries so that the entry and exit angle memories alone might make them wander around excessively, making it difficult for them to advance efficiently toward the food source. Therefore, the fly's behavior in the presence of irregular and dynamic plumes *could* be tested. What would be the behavioral strategy of flies in this complex situation? Will the switching state model still recapitulate the behavior in this scenario?
While the primary goal of our study was to use plume tracking as a paradigm to gain insight into general navigational algorithms in *Drosophila*, we agree with the reviewer that it is important to consider how our results extend to more naturalistic plumes. Our initial manuscript focused largely on stationary odor corridors where the invariant plume geometry allows for precise mapping between a fly's sensory experience and behavior. However, as the reviewer notes, in naturalistic plumes, animals are expected to experience far greater variation in their

entry and exit angles—raising the question as to the utility of memories in these more turbulent contexts and whether the edge-tracking strategy can be used at all?

To address this question, we explored the ability of our switching state space model to track a recorded surface plume previously used to study odor tracking in freely walking flies (Connor et al 2018, Álvarez-Salvado et al 2018). As with most available high-resolution plume recordings, the video spans a relatively short distance—only 300 mm from the source. As a consequence, simulations using the edge tracking model perform very few effective entries and exits along its length, where angular memories can be written or updated (**Extended Data Fig. 14a**). Therefore, to better match the dimensions of our odor corridors and allow access to a larger number of excursions from the plume, we scaled the recorded plume five-fold in space and slowed the playback five-fold in time, generating a plume ~1.5 meters long while preserving the velocity of odor fluctuations. Importantly, preliminary experiments (**Reviewer Fig. 1**) indicate that real flies can effectively track the scaled version of the plume when played back to tethered flies in our virtual reality system.

The structure of 'naturalistic' plumes can vary greatly since their spatial and temporal statistics depend on the fluid dynamics of the underlying currents carrying the odor. Here the recorded plume forms a stable, coherent ribbon near the source but breaks into chaotic filaments downstream—allowing us to evaluate model performance across distinct turbulence regimes. We found that simulations using the average fly model were frequently successful at tracking the plume to its source even when initiated far downwind in the plume's most turbulent region, likely because frequent, chance re-encounters with the odor enable effective navigation even in the absence of memory. By contrast, closer to the plume source, where the plume narrows and becomes more ribbon-like, we found that the crosswind component of simulated entry directions becomes increasingly coherent. Accordingly, the entry memory in our model predicts crosswind entry direction more accurately in less turbulent regions closer to the source, giving rise to more efficient return trajectories than simulations without entry memory. Use of entry memory thus enhances navigation efficiency and success in naturalistic plumes when the turbulence of a plume is sufficiently low for entry angles to be predictive. These analyses form a new **Fig. 5** and **Extended Data Fig. 13**.

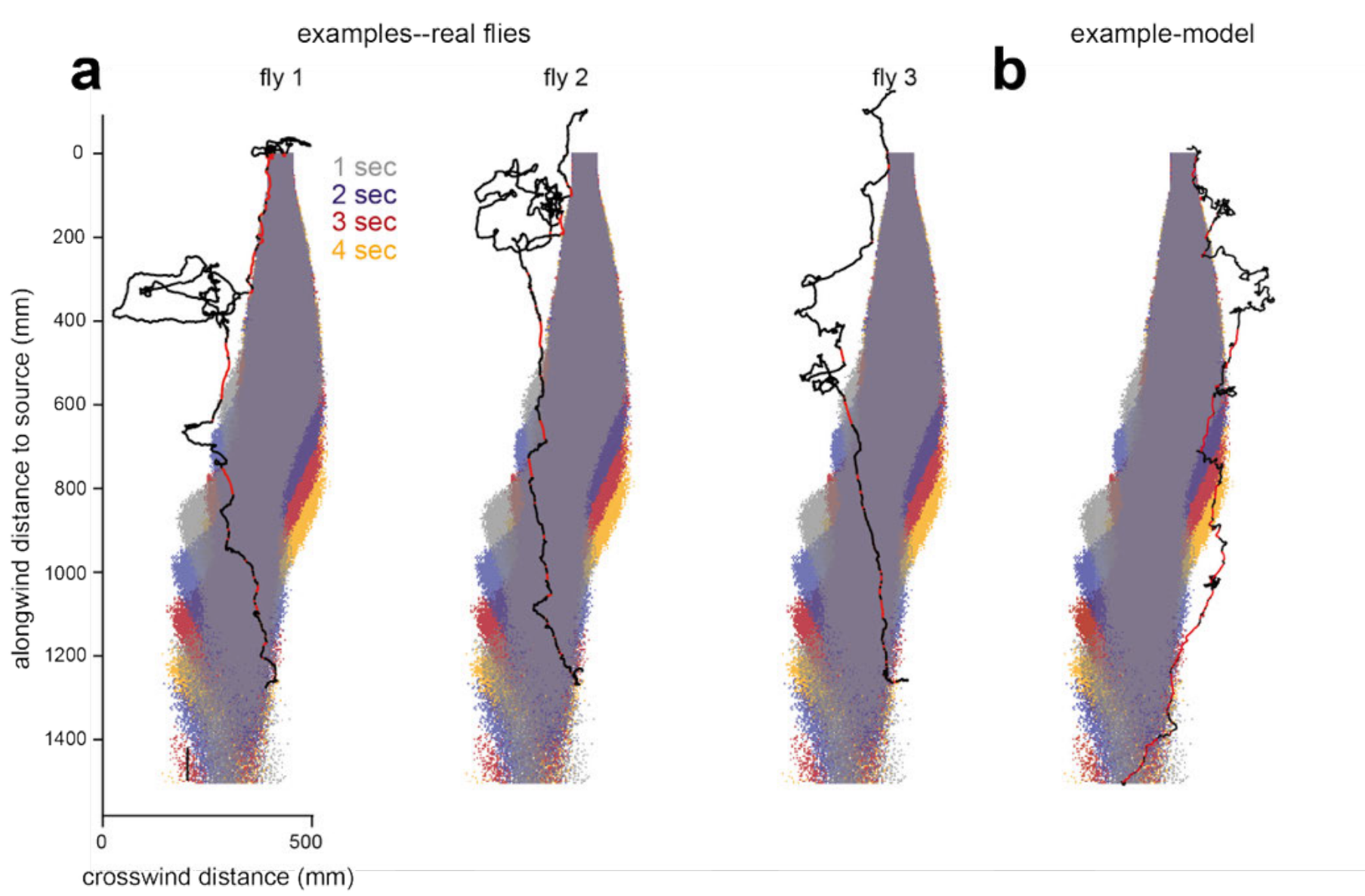

Reviewer Figure 1: Flies track naturalistic plumes. (a) Trajectories of three representative flies tracking the same scaled plume used **in Fig. 5** to analyze how our model contends with more naturalistic turbulent plumes. Red indicates bouts when the fly is in 20% ACV in the binarized plume and black indicates outside bouts when the fly is walking in clean air. Trajectories are overlaid sequential 1-sec snapshots of scaled naturalistic plume overlaid in different colors to highlight differences in plume dynamics along its length. (**b**) Representative simulated trajectory of average model tracking the same plume.

In line with these observations and preliminary experimental data, we now propose that flies likely deploy multiple navigational strategies depending on plume statistics, with edge tracking representing only one such algorithm. Elucidating how flies shift between navigational strategies based on plume structure is an exciting direction for the future but outside the scope of the current manuscript. Nevertheless, we have expanded on this

point in the text (lines 431-433; lines 531-538) to make it clear that flies rely on additional behavioral strategies beyond edge tracking.

3. In experiments where the flies were allowed to track a plume for 10 min before it was removed (described in lines 239-247, Fig. 2i, j), why was this particular time chosen? Was there a correlation between the tracking time and the time it takes for the directional bias to vanish passively? One can ask a similar question in the context of operant training described in Fig. 5. Is it possible to examine the relationship between the number of training and the strength of memory as well as the time constant of memory decay?

We thank the reviewer for this thoughtful question regarding the timescale of memory formation and decay. In experiments in which the plume disappeared, we selected a 10-minute tracking baseline period because most flies re-entered the plume multiple times (typically >3), providing sufficient sampling to ensure formation of a reliable entry-angle memory (**Reviewer Fig. 2**). This duration therefore offered a consistent behavioral baseline from which to demonstrate the persistence of a memory after the plume disappeared.

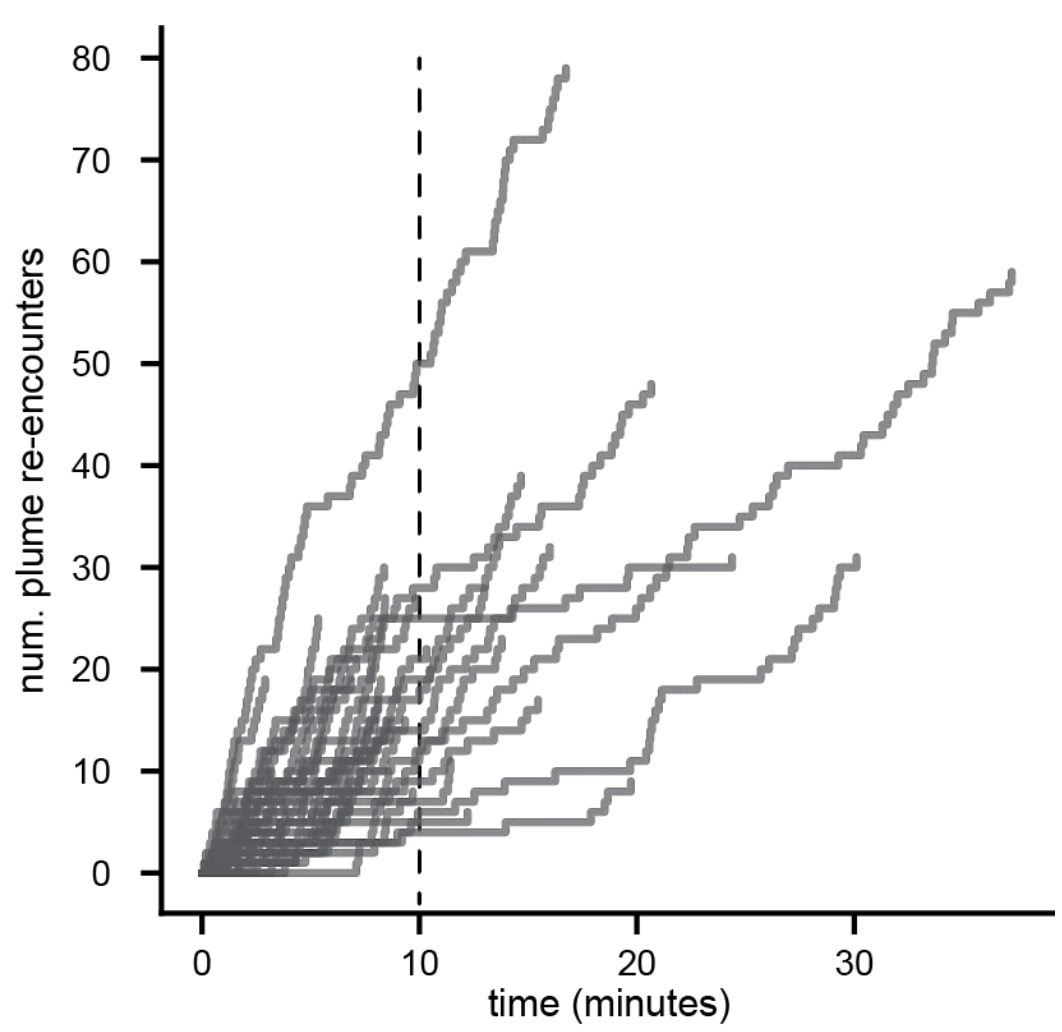

Reviewer Figure 2: Plot showing the number of plume encounters as a function of time for 40 flies tracking vertical (0°) plume.

To explore how long such memories might persist, we reanalyzed data from former **Fig. 1g** and **Extended Data Fig. 5** and now show the distribution of time that flies take on outside excursions. This analysis suggests that lengthy excursions away from the plume can take > 250 seconds to return (**Extended Data Fig. 6e**). Given that our earlier analyses demonstrate that these long returns are far more efficient than a random search, these results suggest that flies retain the stored entry-angle memory across multi-minute timescales. While this does not establish a precise decay constant, it does indicate that memory persists well beyond the time of the last plume encounter.

A more systematic analysis of how memory strength scales with the number of plume entries is challenging because the behavioral readout—directional persistence during clean-air walking—is inherently noisy. This is evident in the fact that the post-plume headings of flies tracking 45° versus 90° plumes are not statistically distinguishable, limiting the precision with which we can extract a memory-strength curve or decay function. Nevertheless, our data support the view that repeated plume encounters strengthen a stored angular goal, and that this memory decays gradually over several minutes once reinforcement ceases.

4. I acknowledge the value of proposing a plausible biological mechanism for storing entry angles as attempted in Figure 6a. However, this is a hypothesis not addressed in this study. Therefore, I would suggest removing or moving the figure panel to Extended Data Figs.

We appreciate that the neuronal schematic was very speculative and have removed it from **Fig. 6a**. We kept the broader conceptual schematic illustrating how the switching state space model alternates between entry- and exit-angle memories during returning and leaving states, as this visually captures the core computational framework supported by our data.

Minor comments:

5. I would suggest to discuss the authors' thought on why flies (and also the switch state model) track the upper part (closer to the wind source) of 45-degree and 90-degree plumes. From what it is shown in Fig. 2a-c, 4a and 5b, there is not a single case of tracking the lower edge.

This insightful observation highlights a key feature of edge tracking—across all plume geometries, when flies enter the odor corridor, they rapidly reorient to walk upwind, giving rise to exit angles that are largely in the upwind direction (**Fig. 2e-g**). As a consequence, flies predominantly track the upper boundary of the plume closer to the wind source rather than the lower boundary. We now discuss how this bias arises in lines 218-220.

Also, most of the edge tracking for these configurations do not stretch as long as the vertical edge tracking as observable in Fig. 2a-c, where the density of trajectories decreases midway especially for the 45-degree plume. Why is this the case?

Although the density of trajectories appears lower for the 45° or 90° plume in **Fig. 2a–c**, when normalizing for scaling and inter-individual velocity differences, flies actually track these plumes just as efficiently or slightly more efficiently on average than the vertical (0°) plume, as reflected in encounters per minute or encounters per meter. We now show this in **Extended Data Fig. 6c**.

6. In Fig. 6c, almost immediately after the fly enters the plume, the FC2 phase becomes aligned to the EPG phase. What could be the mechanisms behind this rapid shift? It would at least be appropriate to show the time posterior to plume entry in the same way as it is shown for the time prior to plume entry in Figure 6j.

We thank the reviewer for this helpful suggestion and have now extended our analysis in **Fig. 6k** to show the EPG-FC2 phase relationship after flies enter the plume. As the reviewer notes, the FC2 phase becomes rapidly aligned to the EPG phase upon plume entry. We speculate that this rapid shift is due to the fact that FC2 neurons integrate from a number of different columnar (hΔ) populations to generate a consolidated goal signal that can be directly compared to a fly's heading direction to drive corrective steering (Mussells-Pires et al. 2024; Hulse et al. 2021). In addition, FC2 neurons receive input from PFN neurons, a columnar population whose activity is offset from the animal's heading by ±45° due to offsets from the protocerebral bridge to the fan-shaped body. Preliminary recordings of hΔ populations pre-synaptic to FC2 reveal that they are active only outside the plume but suppressed inside the plume (data not shown), suggesting that their goal-related signals are selectively conveyed to FC2 neurons only when animals are walking outside the plume and must rely on memory mechanisms to return to its boundary. By contrast, PFN activity has little to no modulation by odor (Currier et al. 2020) allowing them to have greater influence on FC2 signals when odor-responsive hΔ neurons are suppressed. Consistent with this, inside the plume, the FC2 bump not only aligns with the heading direction of a fly but also is significantly more coherent, whereas outside the plume, the FC2 bump is broader, with a higher baseline activity. We now quantify FC2 bump amplitude and coherence in **Extended Data Fig. 14c,d** and discuss these dynamics in the context of how edge tracking alternates between epochs in which flies may rely on continuous sensory signals inside the plume and epochs in which they must depend on memories of past odor encounters (lines 486-494).

7. Although lines 41-44 are a good summary of the work, I feel that the use of the words 'dynamic landmark' is confusing, since the main point of the work is that flies do not memorize a point (landmark) in space.

We agree that the phrase 'dynamic landmark' may be misleading, as our results argue against flies storing the plume's edge as a fixed point in space or stationary landmark. Our intent was to convey that the odor plume itself acts as a moving spatial reference, guiding ongoing behavior. To avoid confusion, we have revised the text to better capture this idea by describing the plume as a 'dynamic angular goal' rather than a 'dynamic landmark.

8. Line 491 describes a prior study on insects that can track the surface of plumes. It might be better to mention this in the Introduction. Also, as Jun et al. eLife (2015) describe edge tracking in freely behaving flies, this reference should be discussed.
Thanks for this suggestion. We have added reference to these previous studies in the introduction to clarify how our studies relate to prior work (lines 83-86).

9. In lines 510-514, the analogy between edge tracking and the ant's navigational strategy is a bit of a stretched. Ants have to memorize several directional vectors associated with visual landmarks, while edge tracking as described in this work, only stores a single vector.
We have removed reference to this analogy.

10. Extended Data Fig.1a and 1c are not referred to in the text.
Thank you for your careful reading, we have now inserted reference to them.

11. Line 400. Fig. 4d does not exist.
Thank you for catching this error. We have corrected it.

12. Line 1104. Singh et al. appears twice in the list (36 and 69).
Thank you for catching this error. We have corrected it.

13. The title of Fig. 1 could describe the biological finding rather than the experimental system.
We agree with this suggestion. We have modified the title for Figure 1 to be more descriptive of the behavior.

14. The bottom right figure in Fig. 1g was hard to comprehend. There may be a way to better describe it.
We appreciate that this panel was very dense. In accord with the suggestion of Reviewer 3, we moved the analysis of the random walk model to the **Extended Data Fig. 5**, where there is more space to describe it and expanded on the figure legend to clarify.

15. Extended Data Fig. 4a. Although the legend describes that "the difference in the timing of odor arrival is < 1 ms, the graph goes beyond 1 ms.
Thanks for noting, we have fixed this.

16. Extended Dat Fig. 5d. Although the legend describes "Note that in contrast to real flies, simulations lacking an upwind bias (a=0) never progress along the length of the plume.", I cannot recognize a difference between real flies and simulations.
We agree that this distinction was not clear in the original figure. The previous legend referred to the theoretical expectation that simulations lacking any upwind bias will, on average, fail to progress upwind; however, this implication was difficult to appreciate at the scale of the plotted trajectories. We have therefore removed this statement from the legend to avoid confusion. Our primary intention with this figure was to show that, although the model with an upwind bias does make progress in the upwind direction, these trajectories are highly inefficient and lack directed returns (e.g., inbound and outbound segments are not statistically different). We now also include representative simulated trajectories in **Extended Data Fig. 5d** to illustrate that, although random search trajectories can return to the plume, they become increasingly inefficient as they move farther from the boundary.

17. Extended Data Fig. 8. How is the goodness of match between the fly and the model evaluated?
We now quantify the correlation between the summary statistics of real and simulated flies in **Table S2**.

18. Extended Data Fig. 9f. Please explain the label for y axis in more detail (entry memory alongwind (mm/s)). Why is the unit mm/s?

In new **Figure 4** describing the model and **Extended Data Fig. 10f and 12d,h**, the y-axis reflects the along-wind component of the stored entry and exit angle memory. Because the model encodes memory as a velocity vector, its components—including the along-wind projection—carry units of mm/s. We now clarify this in the figure legend.

19. Extended Data Fig. 10c. The label c needs to be inserted into the legend.

Thanks for noting, we have fixed this.

Referee #2 (Remarks to the Author):

Summary assessment: This paper presents an exceptional study of how fruit flies navigate structured chemical landscapes, revealing a strategy called "edge tracking" that relies on vector-based computations within the central complex. Using an innovative virtual reality paradigm for olfactory navigation, the authors demonstrate that flies track an appetitive odor corridor by following its boundary, alternating between rapid exits from the plume and directed returns to its edge. The work provides strong evidence that flies store and dynamically update memories of the direction required to return to the plume's boundary, and identifies neural correlates of this directional memory in FC2 neurons of the central complex. This groundbreaking study significantly advances our understanding of sensory-guided navigation and constitutes a major contribution to the field.

Key strengths and contributions of the work: The manuscript presents work of outstanding quality addressing a fundamental question related to sensory navigation with strong relevance to the ethology of flies. The virtual reality paradigm developed for this study offers unprecedented control of plume boundaries with the ability to manipulate these boundaries in closed-loop based on the fly's behavior. This new assay enables the study of navigation behavior with a precision and resolution not previously achieved.

The authors have identified edge tracking as an essential behavior for navigating plumes and demonstrate that this strategy relies on memories of the relative direction to the plume's boundary, not its location. The work shows that flies use the plume's boundary as a dynamic landmark, remembering it as a chemical signpost. The stimulation protocols take full advantage of the virtual olfactory environment to test specific hypotheses about plume tracking. The authors propose a series of ingenious experimental variants, including the "jumping plume" paradigm that elegantly demonstrates the role of directional but not positional memory.

The data analysis and modeling create exemplary synergy to formulate and test hypotheses. The switching state space model derived from behavioral data captures the essence of edge tracking behavior and provides insights into the timescales of memory formation and updating. The authors make a solid case for the involvement of the central complex in plume navigation through loss-of-function experiments with EPG neurons. The finding that FC2 neurons signal the direction back to the odor boundary during returns provides an entry point into understanding the neural circuit correlates of directional memory.

Several clever control experiments strengthen the work's conclusions, including Orco>CsChrimson controls to test the role of left-right comparison (Extended Figure 4), tests of longitudinal gradients to rule out their contribution (Extended Figure 3), sensory replay experiments to demonstrate that edge tracking requires using recent entry and exit angles to update goal direction (Figure 4), and operant training protocols showing that entry angles can be dynamically updated through successive odor encounters (Figure 5).

This manuscript makes a superb contribution to our understanding of insect navigation, revealing that odor tracking is not merely a reflexive process but incorporates sophisticated vector-based computations. The work

demonstrates impressive methodological innovation, advanced and meticulous data analysis, and a seamless integration of behavioral experiments with computational modeling. The findings advance our understanding of olfactory navigation in flies while simultaneously offering profound insights into fundamental principles of sensory-guided navigation conserved across diverse species.

We thank the reviewers for their enthusiasm for this work and their thoughtful comments and suggestions below.

While this is already an outstanding manuscript with exceptional experimental design and data analysis, I offer the following suggestions to further enhance the clarity of certain results and strengthen how key conclusions are established:

1. Analysis of traversal gradients: The authors convincingly demonstrate that flies do not follow longitudinal odor gradients in their assay, instead exploiting edge tracking. It would be valuable to examine how flies respond to the presence of a more graded traversal odor gradient. Would they switch from edge tracking to meandering across the center of the trail, or would they still focus on edge tracking? This would address the authors' speculation that edge tracking might be a strategy to avoid sensory adaptation.

We thank the reviewer for this useful suggestion. To examine how flies track graded odor plumes, we generated odor corridors with lateral concentration gradients following the Gaussian profile characteristic of naturalistic plume envelopes (Connor et al., 2018 or Alvarez-Salvado et al, 2018). To make these graded plumes most comparable to the constant-concentration odor corridors used throughout the manuscript, we set the peak odor concentration to be 20% at the plume's midline and adjusted the width such that the half-maximal concentration occurred at ±25 mm.

In our new **Extended Data Fig. 3**, we compare how the same flies tracked constant-concentration and graded plumes. We found that even in a smoothly graded plume, they did not meander near the center. Instead, they appeared to track along a perceived boundary, with an occupancy peak at ~25 mm from the midline, close to the maximal slope of the concentration gradient. This observation aligns with evidence that *Drosophila* sensory neurons are sensitive to the derivative of odor concentration (Kim et al., eLife, 2015). These experiments suggest that flies may maximize information by positioning themselves along the region of steepest concentration change—a level of behavioral precision that would have been difficult to uncover without the controlled virtual environment. Although outside of the scope of the current experiments, this will be an interesting direction to explore in the future.

2. Correlation between exit and entry angles: While the data are quantified in multiple ways, a relatively straightforward analysis of the relationship between exit angles and subsequent entry angles could be informative. For vertical plumes, are larger exit angles correlated with larger subsequent entry angles? This analysis might reveal whether flies are capable of fine-grained course correction at the level of their (first) turn toward the plume upon its exit.

Whether larger exit angles predict larger subsequent entry angles in vertical (0°) plumes—and thus reflect fine-grained course correction on the first turn back—is an important question. Although we do observe a correlation between exit and subsequent entry angles in vertical edge-tracking trajectories, a similar correlation is also observed in simulated trajectories where entries are not informed by prior exits (**Reviewer Figure 3**). This

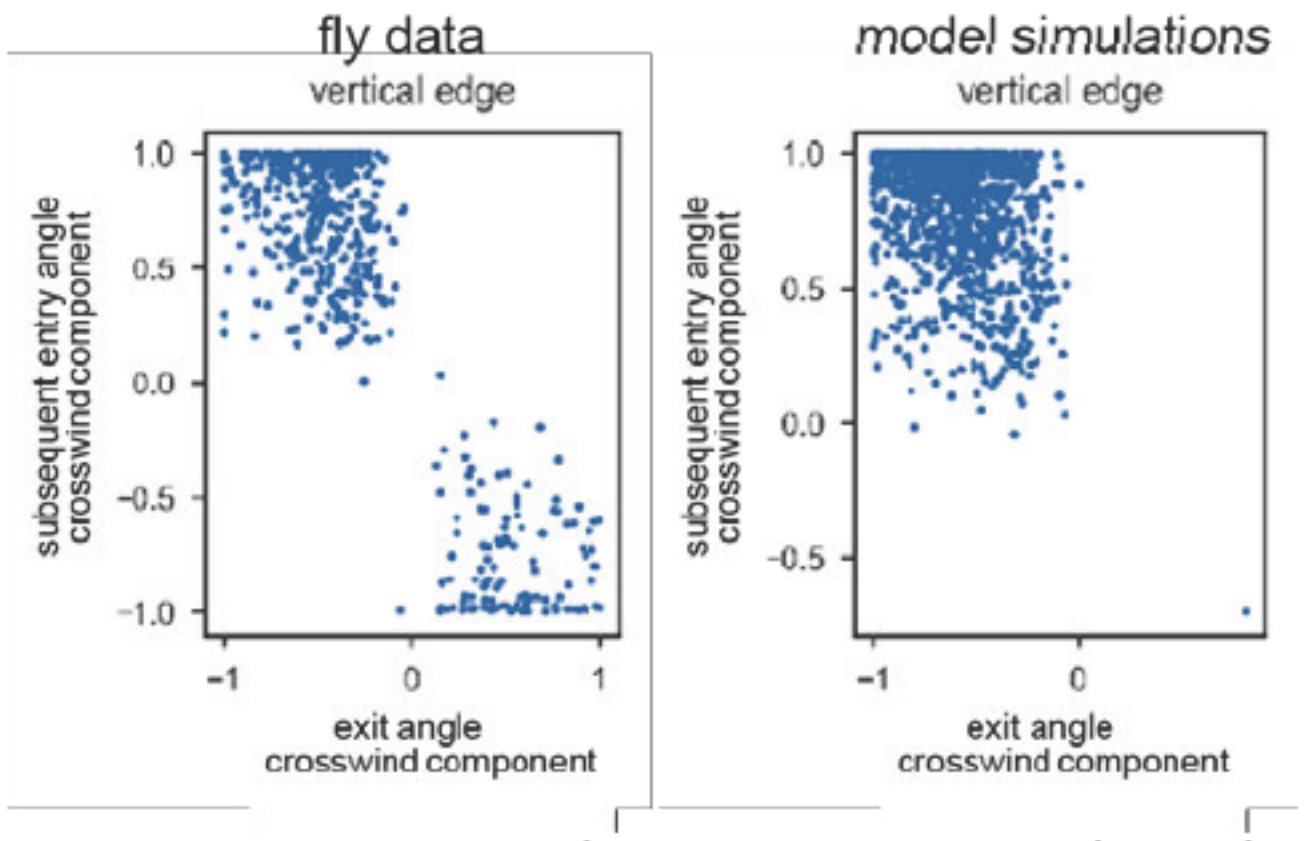

Reviewer Figure 3: Plot comparing exit angles and subsequent entry angles in real flies (left) and simulations (right). All trajectories flipped so real and model flies track the left side of the plume. Correlation of left quadrant: For flies: PearsonR =-0.24 (p = 2.9e-06). For model: PearsonR =-0.20 (p = 1.7e-11).

correlation likely originates from the fact that if a simulated fly exits close to the upwind direction, it is more likely to re-enter the plume near that direction.

One intuition also suggested by the reviewer's question is that flies could simply use a single angular memory—e.g. using their exit angle to guide subsequent returns. Notably, this could not work for all plume geometries since flies leave the plume in the upwind direction for both 0° and 90° plumes but return in the upwind direction for a 0° plume and in the downwind direction for a 90° plume.

3. Learning curve for plume encounter efficiency: Figure 5 convincingly demonstrates that entry angles can be updated through just a small number of plume encounters. It would strengthen the manuscript to more thoroughly document how the precision of returns to the plume improves with successive encounters across different plume geometries. While Extended Figure 10a touches on this analysis, a more systematic characterization of this learning trajectory would be valuable. Establishing this behavioral feature earlier in the manuscript would provide important context for the sophisticated manipulations that follow and further support the memory-based nature of edge tracking.

We appreciate this suggestion. Both the experimental and simulated trajectories in former **Fig. 5 (now Fig. 4e-h)** show that flies update their entry angle memories rapidly and thus may become more efficient on their first few re-entries as they strengthen the angular memory that guides them back to the plume's boundary. We agree with the reviewer that this feature of edge tracking would be helpful to describe earlier and more explicitly in the manuscript. We have therefore moved reference to this analysis to **Extended Data Fig. 7a,b** and now compare the trajectories during the first five bouts outside the plume providing a clearer view of how return efficiency develops with experience. As described in our initial manuscript, the increase in efficacy—now shown for both distance and duration of outside bouts—is significant for the 45°, 90° and jumping plumes, presumably because these configurations cause flies to be displaced further from the plume's edge and thus reduce the likelihood of incidental returns. Such rapid adaptation is consistent with the results from the operant training paradigm (updated **Fig. 4e-h, Extended Data Fig. 11**) which reveals these dynamics even more clearly because entry angles can be systematically manipulated.

4. Visualization of random search model trajectories: In Figure 1g, the authors discuss results of synthetic trajectories generated with a random-search model, but no example trajectories are shown. Including representative trajectories would help readers better understand the contrast with real fly behavior, strengthening the conclusion that edge tracking does not follow the statistics of a random search process.

We have now included representative trajectories to **Extended Data Fig. 5d** to highlight the difference between real and synthetic trajectories.

5. Integration of plume statistics: The authors propose that tracking the plume envelope rather than wind direction might be advantageous (line 196). This hypothesis could be strengthened by quantitatively relating it to the observed timescales of fly responses and memory updating, compared with statistical properties of natural odor plumes. Incorporating quantitative features from the literature on plume statistics (e.g., Celani and Vergassola, Cardé and Willis) would help establish whether the memory mechanisms observed in this study

operate on timescales compatible with the dynamics of natural plumes that flies would encounter in their environment.

While the primary goal of our study was to use plume tracking as a paradigm to gain insight into general navigational algorithms in *Drosophila*, we agree with the reviewer that it is important to consider how our results extend to more naturalistic plumes. Our initial manuscript focused largely on stationary odor corridors where the invariant plume geometry allows for precise mapping between a fly's sensory experience and behavior. However, as the reviewer notes, in naturalistic plumes, animals are expected to experience far greater variation in their entry and exit angles—raising the question as to the utility of memories in these more turbulent contexts and whether the edge-tracking strategy can be used at all?

To address this question, we explored the ability of our switching state space model to track a recorded surface plume previously used to study odor tracking in freely walking flies (Connor et al 2018, Álvarez-Salvado et al 2018). As with most available high-resolution plume recordings, the video spans a relatively short distance—only 300 mm from the source. As a consequence, simulations using the edge tracking model perform very few effective entries and exits along its length, where angular memories can be written or updated (**Extended Data Fig. 14a**). Therefore, to better match the dimensions of our odor corridors and allow access to a larger number of excursions from the plume, we scaled the recorded plume five-fold in space and slowed the playback five-fold in time, generating a plume ~1.5 meters long while preserving the velocity of odor fluctuations. Importantly, preliminary experiments (**Reviewer Fig. 1**) indicate that real flies can effectively track the scaled version of the plume when played back to tethered flies in our virtual reality system.

The structure of 'naturalistic' plumes can vary greatly since their spatial and temporal statistics depend on the fluid dynamics of the underlying currents carrying the odor. Here the recorded plume forms a stable, coherent ribbon near the source but breaks into chaotic filaments downstream—allowing us to evaluate model performance across distinct turbulence regimes. We found that simulations using the average fly model were frequently successful at tracking the plume to its source even when initiated far downwind in the plume's most turbulent region, likely because frequent, chance re-encounters with the odor enable effective navigation even in the absence of memory. By contrast, closer to the plume source, where the plume narrows and becomes more ribbon-like, we found that the crosswind component of simulated entry directions becomes increasingly coherent. Accordingly, the entry memory in our model predicts crosswind entry direction more accurately in less turbulent regions closer to the source, giving rise to more efficient return trajectories than simulations without entry memory. Use of

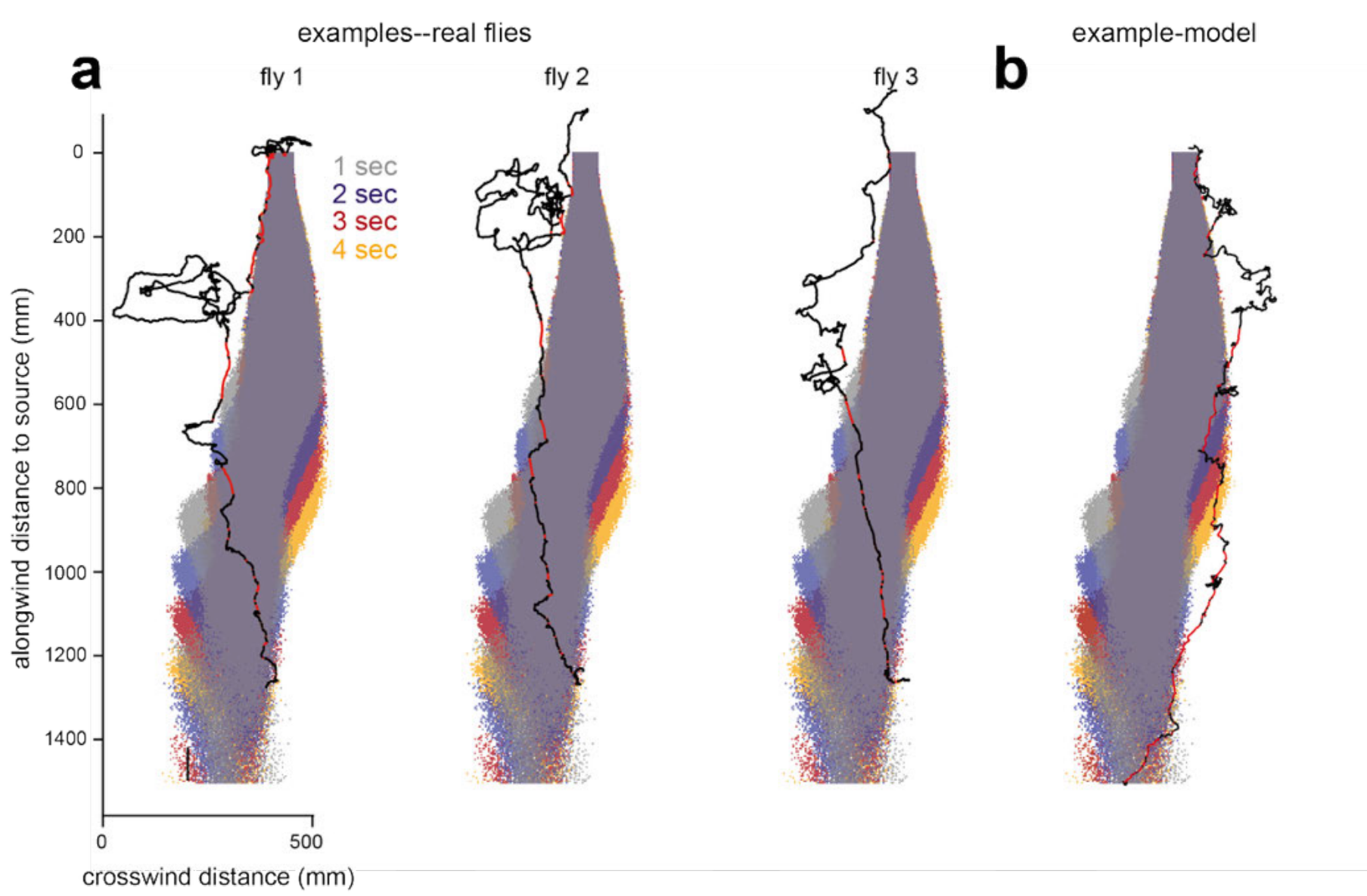

Reviewer Figure 1: Flies track naturalistic plumes. (a) Trajectories of three representative flies tracking the same scaled plume used **in Fig. 5** to analyze how our model contends with more naturalistic turbulent plumes. Red indicates bouts when the fly is in 20% ACV in the binarized plume and black indicates outside bouts when the fly is walking in clean air. Trajectories are overlaid sequential 1-sec snapshots of scaled naturalistic plume overlaid in different colors to highlight differences in plume dynamics along its length. (**b**) Representative simulated trajectory of average model tracking the same plume.

entry memory thus enhances navigation efficiency and success in naturalistic plumes when the turbulence of a plume is sufficiently low for entry angles to be predictive. These analyses form a new **Fig. 5** and **Extended Data Fig. 13**.

In line with these observations and preliminary experimental data, we now propose that flies likely deploy multiple navigational strategies depending on plume statistics, with edge tracking representing only one such algorithm. Elucidating how flies shift between navigational strategies based on plume structure is an exciting direction for the future but outside the scope of the current manuscript. Nevertheless, we have expanded on this point in the text (lines 431-433; lines 531-538) to make it clear that flies rely on additional behavioral strategies beyond edge tracking.

6. Comparison of ACV vs. Orco>CsChrimson behavior: Extended Figure 4 suggests that flies undergoing Orco>CsChrimson stimulation may spend more time inside the virtual odor corridor than flies experiencing stimulation with real vinegar (ACV). A more direct comparison of the probability distributions of positions would help clarify whether the edge tracking behavior observed with ACV generalizes to other odor stimuli.

We thank the reviewer for this suggestion. In our initial submission, we directly compared the positional probability distributions of flies tracking an ACV corridor versus a light corridor in which Orco>CsChrimson flies were activated. In both cases flies edge track, with occupancy peaks aligned to the corridor boundaries, supporting that flies adopt a similar spatial distribution with either real odor or optogenetic activation. Aligned with the reviewer's suggestion we now directly compare several key behavioral metrics for flies tracking an ACV corridor versus Orco>CsChrimson activation and show that while they are largely comparable, both inside and outside trajectories are shorter in the optogenetic paradigm, and flies are slower inside the optogenetic plume in comparison to ACV. This additional analysis (new **Extended Data Fig. 4f,g**) thus suggests that flies track the edge of optogenetic corridors more tightly on average, with shorter inside and outside excursions. These differences could arise from differences in the timing of sensory neuron activation or differences in the strength or distribution of activated sensory neurons. Importantly, however, they support that flies can edge track even when both antennae are synchronously activated.

7. Response to wind direction changes independent of movement: An interesting extension would be to test how flies respond when the wind direction changes independently of their movement. This would help address how flies distinguish changes due to their own actions from those due to external factors, a key challenge in natural environments with stochastic airflows. While this may exceed the scope of the current work, it would connect to the authors' speculation about "more reflexive strategies" when odor encounters are truly stochastic (lines 529-530).

We agree that this is an interesting question, since if wind is used as an allocentric landmark, shifts in its direction could potentially confound a fly's ability to use it as a compass cue. Indeed, in our own open-loop experiments we observed that EPG activity does not immediately follow externally imposed changes in wind direction (data not shown), consistent with prior findings from Rachel Wilson's lab (Okubo et al., *Neuron*, 2020; Basnak et al., *Nature Neuroscience*, 2025) that shifts in wind direction do not consistently alter the position of the EPG heading signal in walking flies. These observations suggest that the compass does not immediately update with shifts in the wind direction but rather integrates wind direction with a fly's own movement history. In addition, previous work on wind-responsive PFN neurons (Currier et al., *eLife*, 2020; Ishida et al., *Cell*, 2025) suggests how sudden gusts of wind could potentially shape goal representations within the fan-shaped body. While a systematic analysis of how wind direction is dynamically represented in the central complex is beyond the scope of the present study, we view this as an important direction for future work, particularly in more turbulent plume environments where both odor signals and wind direction fluctuate.

8. Evolutionary context: While the discussion of evolutionary conservation of navigational toolkits is insightful, there remains a gap between what is demonstrated and what is speculated. The authors might consider tempering some of these assertions or more clearly distinguishing between established findings and hypotheses.
We appreciate the reviewer's thoughtful point and have modified the text to temper our assertions accordingly. Our intention was to highlight that similar types of vector-based computations—such as storing and updating directional goals—are employed across different behavioral contexts in insects. We have now revised the text to remove potentially confusing comparisons to the navigation of central place foragers and to explicitly distinguish the angular memory described here from the positional memories that guide ants and bees to precise nest locations (lines 38-40; lines 267–268; lines 272–274; lines 518-520).

Minor points: Extended Figure 10 appears to be missing the label of panel (c) in its caption.
Thanks for noting this.

I found no other typos in the manuscript and wish to commend the authors for the exceptional quality of the manuscript, the clarity and neatness of the figures, and the thoroughness of the methodology.
Thank you for your enthusiasm.

Referee #3 (Remarks to the Author):

This paper provides novel and convincing evidence that the tracking of chemical plumes by *Drosophila* involves memory, encoded in the central complex, of the direction (relative to the wind direction) of movement of the animal when crossing the edge of the plume. This enables an edge following strategy, including extended periods outside the plume, towards the source. This strategy is suggested to exploit the saliency of the sharp change in odour intensity at the edge, rather than trying to detect and follow shallow or non-existent gradients in plumes, and to be more efficient that the surge-and-cast strategy previously thought to explain insect plume following.

My main concern with the work, as presented, is that the authors make very free, and in my view rather inaccurate, use of navigational terminology, such as 'landmark' and 'vector' in describing their findings. Indeed the mechanism proposed seems to be a form of menotaxis: the animal remembers a desired heading direction (the direction it was travelling when odour was encountered) relative to an external directional cue (the wind) and can flexibly switch to travelling in this direction to regain contact with the edge of the plume. As such, there are no landmarks ("dynamic" or otherwise) or vectors (i.e. representations of "direction + distance") involved, and comparisons to ant homing and route following are just misleading. In my additional comments I have highlighted specific instances where I find this framing misleading or confusing.

We thank the reviewer for these comments. We agree that it is important to be precise and appreciate the opportunity to clarify both our conceptual framework and how it is presented. As we detail in our responses below, we are in complete agreement that there is no evidence for positional encoding in our data—flies in our paradigm do not return to a fixed location in space, nor do they appear to store or compute a positional vector that combines both direction and distance to a goal. Rather our results suggest that the vector memory used during edge tracking is a velocity vector, not a position vector—consistent with other central-complex models of goal-directed navigation, including those proposed to be operant during menotaxis.

Importantly, in accord with the reviewer's thoughtful suggestions, we have revised the text to clearly distinguish between the directional memory used in edge tracking and the positional memory used during nest homing. We now also make clear that the plume's boundary does not serve as a stable spatial landmark but rather provides a directional reference that is continuously updated through odor encounters. Comparisons to ant navigation are therefore much more limited and framed only at the level of shared computational elements (e.g., storing

directional goals in the central complex) rather than implying mechanistic equivalence in positional mapping or distance estimation.

The manuscript is otherwise excellent in most respects, presenting a very thorough exploration of the behaviour and the neural correlates, accompanied by explanatory modelling. However I suggest it could be improved by consideration of the following points:

1) It is not fully clear what is the natural correspondence to simulated plumes that cross the wind direction at angles up to 90 degrees. For example, would the odour source itself need to be moving to produce such a plume? There is brief discussion of this around line 193, referring to transient changes in wind direction. It would be helpful to illustrate what exactly it would mean to 'track the envelope of the plume rather than the wind direction'.
We regret that this point was not clear. Prior work visualizing the spatial structure of plumes with smoke tracers or fluorescent volatiles, as well as models of turbulent odor transport (David et al., *Nature*, 1983; Young et al., *Exp Fluids*, 2021; Farrell et al *Environmental Fluid Mechanics*, 2002) reveal that the centerline of a plume often meanders due to transient fluctuations in wind direction, such that the plume's longitudinal axis can become substantially misaligned with the prevailing wind. As a consequence, insects often encounter odor plumes whose boundaries are offset from the wind direction (Cardé and Willis, *J Chem Ecol*, 2008; David et al., *J Chem Ecol* 1982), a feature we explicitly mimic with the 45° and 90° plumes used in our study.

Meandering plumes pose a challenge for purely reflexive strategies—such as odor-gated anemotaxis—because surging directly upwind would inevitably carry an animal out of the plume. This has led to the proposal that a more adaptive solution is to track the plume's boundary or envelope rather than simply the wind direction. Consistent with this idea, recent computational work (Singh et al., *Nat. Mach. Intell.*, 2023) showed that RNN agents trained on meandering plumes adopt a "plume-skimming" strategy that closely resembles edge tracking. Our virtual environment allows us to experimentally test this proposal by creating odor plumes whose centerline and boundaries are arbitrarily offset from the wind direction.

We have now clarified this point in the revised text (lines:201-205) and added the relevant citations to highlight the prevalence and ecological importance of meandering plumes in nature.

2) It is really not clear to me what the replay experiments are meant to prove (fig 4). It seems self-evident that (line 321) "odor encounters uncoupled" from the animal's movement through space will not produce coherent spatial behaviour, other than perhaps evoking upwind turning, which is what seems to happen. Given fig 2 already shows flies "maintain a directional bias based on their past plume encounters" (line 341), the replay results at most seem to show one or two inconsistent encounters with odour quickly erode this tendency. This is more convincingly shown in the later operant experiments (fig 5) so I think this whole section and figure could be dropped (or made supplementary) simplifying the paper.
We agree that our motivation for the replay experiments was not sufficiently clear in the original submission. We have therefore moved these experiments to a supplementary figure (**Extended Data Fig. 12**) and introduce them later in the manuscript where, rather than provide behavioral evidence for an angular memory, the replay experiments now support the ability of our model to predict how flies adapt to changing sensory experience. In their new position in the revised manuscript, the replay experiments provide a useful test of the switching state–space model: after prior plume tracking, flies show an initial return bias that rapidly decays when encounters are inconsistent, and thus where directional memories are no longer reinforced by coherent entry or exit angles (**Extended Data Fig 12e-h**).

3) My impression of the model results is that it is not necessary to assume the fly has two memories (entry and exit angle) but that the exit could be explained by the fly consistently turning into and then slightly past the wind

direction when it is in the plume. The FC2 activity results also seem to indicate only setting of a goal direction towards the plume. This possibility is briefly addressed in the discussion (lines 559-562) but as a single memory would be a more parsimonious account, which is still consistent with the data, I think it should be given more weight.

The possibility that a *single* goal memory—updated only upon plume entry—could account for our behavioral data is important to consider, particularly given the asymmetry we observe in the relative influence of the entry and exit angle memory to successful plume tracking. However, several features of the data argue against a purely reflexive strategy to guide plume exits. First, as **Fig. 2e-f** highlight, while flies exit largely in the upwind direction for all plumes, the relationship between entry and exit angles systematically varies with plume orientation. Reflexively turning slightly past the upwind direction, as the reviewer suggests, would work to track a 0° plume but not a 90° plume, where entries and exits share the same crosswind projection—a feature that is important to maintain consistent progress in the crosswind direction without backtracking. Second, flies tracking a 0° plume require a bias in their exit angle since without it they cannot adhere to a single edge and frequently cross over the plume (**Extended Data Fig. 10b-e** in the revised manuscript). Finally, some individual fly trajectories do show evidence of exit-angle updating during operant conditioning trials when the plume geometry changes (**Extended Data Fig. 10f**), although this is challenging to quantify, potentially because exits are predominantly in the upwind direction.

Thus, flies appear to require a consistent bias in their exit angle that depends on plume orientation, motivating our inclusion of an exit-angle term in the model. As the reviewer notes, there is no clear neural correlate of the exit angle memory in FC2 neurons. We therefore speculate that this steering bias could be conveyed by other columnar neurons in the central complex or possibly by circuits outside the central complex altogether. Given that the origin of the exit bias is not known, we have taken the simplest approach of treating entry- and exit-angle memories as being implemented in the same computational form within our model. However, in the revised manuscript we now emphasize that the entry angle memory and exit steering bias play distinct functional roles—with entry angles instructing an angular goal to guide returns to the plume's boundary whereas exit angles define a stable steering bias (lines 366–368) and expand the discussion to outline alternative circuit mechanisms that could generate this exit bias (lines 563–570).

Additional comments:

Line 1: The first sentence of the abstract seems both disputable (odors are not essential for navigation, and odors might guide behaviour without requiring navigation) and unnecessary.

We appreciate the reviewer's comment and have revised the first sentence of the abstract.

Line 45, if the memory only indicates a direction, and not a distance, then it might be better not to so prominently feature the term 'vector' throughout the paper (including the title) as 'vector' strictly implies direction and distance (angle and length). Although the term 'vector' is often used loosely in biology to refer only to direction, I think it is crucial to clear understanding of the neural mechanisms of navigation to maintain a distinction between the animal having directional guidance only vs. an intended travel vector with an explicit endpoint.

In our model, directional memories are indeed represented as vectors, not simply as angles but, as stated above, they are velocity—not position—vectors. Upon each plume entry, the model updates a goal-velocity vector by adding unit vectors in the direction of entry, scaled by an update parameter b (in mm/s). The angle of this vector encodes the remembered direction, while its magnitude reflects both the coherence of past entries and the goal speed used for velocity updates. Thus, even though the behavioral memory is directional, its mathematical representation—and its influence on movement—is inherently vectorial. Importantly, this also aligns with models of how angular goals are neurally represented as vectors via sinusoidal patterns of activity within the *Drosophila* central complex.

The reviewer makes an important distinction, however, that the memory we describe is only directional and not positional. In the revised text, we now emphasize this distinction explicitly throughout the results and discussion (e.g. lines 38-40; lines 267–268; lines 272–274; lines 518-520).

Line 55-60: I assume that the validation of the chemical landscape produced by the system is provided in previous papers (although the references 15,16 do not have obviously relevant titles). A more explicit reference to how the system was validated (i.e. measured to be producing the intended odor landscape) would be helpful.
The plume boundaries in our paradigm are created by coupling command signals to the mass flow controllers that infuse odor into the wind stream to the fly's XY position as it walks in virtual space. Therefore, to validate the odor landscapes generated in our virtual reality system, we measured odor onset and offset relative to the control signal driving the mass flow controllers (**Extended Data Fig. 1a**). These measurements confirmed that odor timing accurately reflects the programmed plume boundaries. We also quantified how the fly's own walking speed at the time of odor impacts the effective location of the plume's boundaries, introducing small variations in the exact position of the boundary that the fly experiences (**Extended Data Fig. 1b,c**). We now describe this validation explicitly in the main text (lines 68-70).

Line 60-62: Is it certain that the fly has no other sensory cues for orientation in this paradigm? Presumably it still has proprioceptive cues to its own rotation, for example. I appreciate that the wind cue is probably sufficiently dominant for the purposes of the experiment, but other possible cues should at least be mentioned.
Thank you for raising this point. Our intention with our initial description of our paradigm was to convey that wind provides the dominant external directional cue available to the fly. Indeed, in the absence of wind, flies appear unable to maintain directed trajectories, as evidenced by the highly convoluted, undirected paths during optogenetic activation of olfactory sensory neurons without wind (**Extended Data Fig. 4c**). Nevertheless, we agree with the reviewer that flies do retain access to proprioceptive cues that may contribute to further stabilizing their orientation during navigation. We have clarified the text accordingly (lines 55-57).

Line 90: a 95% confidence interval of 1.91 to 2.15 seconds spent in the plume seems surprisingly tight, as though flies are performing a very fixed response (consistent with the possibility above that they do not need a memory to direct their exit of the plume. Perhaps this could be characterised further, maybe excluding the 4% of times the flies crossed to the other side of the plume?
We have performed the proposed analysis, which confirms that flies indeed spend very little time in the plume. Excluding inside trajectories that cross to the opposite side of the plume yields a mean inside-bout duration of 4.4 sec (95% CI: 3.6–5.3 sec), emphasizing how rapidly flies counterturn to exit rather than sample the interior of the odor corridor. We also re-examined how confidence intervals were computed and found that our original approach, which applied a Box–Cox transformation prior to estimating confidence intervals, effectively produced confidence intervals closer to the median. We therefore re-calculated confidence intervals using a nonparametric bootstrap approach, which is more intuitive and does not rely on distributional assumptions giving rise to time inside = 4.9 sec, 95% CI: 4.1–5.8 sec). We now note this (lines 99-101).

Line 311: I agree these results show the flies efficiency in tracking the plume is influenced by EPGs, but do the EPG-deficient flies not still exhibit a form of (less efficient) edge tracking?
We thank the reviewer for this question. When EPG neurons are silenced, flies are unable to reliably return to the plume's boundary and when they do return it is often hundreds of meters upwind, due to the fact that they take far less efficient trajectories, similar to a random search process. We now provide a clearer representation of this deficit by showing the average trajectories of flies when EPG neurons are active and silenced (**Fig. 3f**). In the revised manuscript, we now also show that silencing of FC2 neurons gives rise to a similar deficit as EPG silencing (**Fig. 6b**). Edge tracking therefore appears to rely on both the neurons that encode the entry angle memory and the allocentric reference frame in which that angular memory is stored.

Line 315: Is it essential that flies track both entry and exit angles? The evidence provided here (lines 334-335) seem to suggest that odor onset causes steering upwind, not requiring memory, whereas the behaviour after odor offset (in this case a downwind bias) cannot be explained without memory.
Thank you for raising this point. As the reviewer notes—and as we address in more detail in our response to their related comment on the necessity of having both an entry and exit memory in point (3) above— while flies exit largely in the upwind direction, they appear to require a consistent bias in their exit angle that depends on the orientation of the plume. For these reasons, we included an exit angle memory in our model. Nevertheless, we have revised the text throughout the manuscript (e.g. lines 366-368; lines 491-493; lines 563-570) to make it clear that the entry and exit angles are not equivalent in their influence on edge tracking and present alternative models of how they may be implemented.

Regarding the section title of line 315, based on the thoughtful suggestion of the reviewer, we have also reorganized the results section to move the replay experiments to the supplementary material and now removed this title.

Line 492-3 I do not think it is appropriate to describe memory of a heading direction as 'an olfactory landmark'.
We agree that the phrase 'olfactory landmark' may be misleading given that our results argue against flies storing the plume's edge as a fixed point in space. Our intent was to convey that the odor plume itself acts as a moving spatial reference, guiding ongoing behavior. We have revised the text to remove reference to the plume as a landmark to avoid confusion.

Line 500-501 I think it is extremely misleading to describe this as a "vector based strategy similar to what central place foragers use to home to their nests". It is absolutely crucial for homing to integrate the directional and distance information in travel vectors. As the following sentences makes clear, there is no evidence that vectors are involved or required for plume edge-tracking, only directions.
We agree that central place foragers are thought to navigate back to their nests through path integration, relying on positional memories that include both direction and distance. By contrast, during edge tracking appears to only require an angular memory to return to the plume boundary. The conceptual point we wished to convey was simply that edge tracking is neither a reflexive nor egocentric navigational strategy. Rather, it relies on a vector-based computation in the sense that flies store a direction anchored to an external allocentric reference frame, which is used to guide the fly's movement back towards a goal direction. Edge tracking therefore appears to rely on some of the same elementary components of vector-based navigation used central place foragers, even though it lacks the distance term essential for true homing.

We have revised this sentence to make this important distinction clear, now noting that edge tracking shares key computational features of the vector-based navigation used by central place foragers (lines 514-515). We have also revised the text to explicitly distinguish the angular memory described here from the positional memories that guide ants and bees to precise nest locations (lines 38-40; lines 267–268; lines 272–274; lines 518-520).

Line 511-512 Work on ant navigation over the past decades has cast doubt on the 'local vector' account of route following, as it is possible to use rotational match of visual snapshots to recover directions without requiring any association to a vector. See e.g. Collett, M., Chittka, L., & Collett, T. S. (2013). Spatial memory in insect navigation. Current Biology, 23(17), R789-R800. There is also a big difference between 'serially sampling sensory landmarks' in the sense of ants storing multiple snapshots (indicating fixed locations along a route) and in the sense of the fly updating a single estimate of the desired direction to re-enter the plume – these are simply not comparable in my view.

We agree that the underlying mechanisms differ in important ways making this analogy too superficial. We have revised the text to remove this reference.

Overall the figures are very dense and the authors might want to consider removing some of the subfigures (or moving them to supplementary material). Some specific comments:

Fig 1d) is extremely hard to read (even magnified on screen and it is not clear it is necessary. 1g is not very intuitive, and overall, I think the demonstration that random path segments do not reproduce the edge following could be kept wholly as supplementary, as it is not a surprising finding, although useful as a basic check.
We appreciate this input. While we think it is important to characterize some of the basic metrics of edge tracking in this initial figure (**Fig. 1d**), we have moved the random search model (formerly **Fig. 1g**) fully to the supplement (**Extended Data Fig. 5**), allowing us to reorganize and simplify the panels of this figure for clarity.

2i and j, any comment on the lack of difference for 45 and 90 degrees after plume is gone? Could this be indicative of a simpler strategy – setting a goal either 90 degrees or 180 degrees to wind direction? Could it be explored in the model whether a very approximate goal is sufficient?
We agree that the lack of a statistical difference in the post-plume headings for flies tracking 45° versus 90° plumes may suggest a simpler strategy for storing a goal. However, the heading measured in clean air after the plume disappears is a relatively noisy behavioral readout—in part because it is challenging to resolve how the memory decays in individual flies once reinforcement ceases. The operant training paradigm (**Fig. 4e-h**) provides a clearer and more interpretable readout of memory updating, which is why we rely on it to demonstrate angular learning more explicitly.

Regarding the possibility of a simpler rule—for example, storing only an approximate goal that is 90° or 180° relative to the wind—we think this is unlikely for two reasons. First, as shown in **Fig. 2e–h**, the entry angles used during edge tracking are not clustered around fixed directions but instead vary systematically with plume geometry. Second, from the perspective of our model, restricting the returning goal to either purely crosswind (90°) or purely up/downwind (180°) would require the fly to discard either the crosswind or along-wind component of its entry directions. Because neither real nor simulated flies have access to the true orientation of the plume's edges—they only know their own heading direction relative to the wind direction when they encounter the odor—there is no principled way to select which component to ignore. Our model therefore incorporates an angular memory that is continuously updated with each plume entry. Notably, while the amplitude of this goal signal can be small, it is important that it points in an effective returning direction, as emphasized by the fact that removing this memory from the model results in significantly poorer edge tracking (**Extended Data Fig. 10a,e**).

Fig 4, See my comments above, it is also quite hard to understand what is plotted in these figures, and seems somewhat pointless to plot 'entry direction' for flies that are experiencing 'entry' (odour onset) uncoupled to their action.
We appreciate the reviewer's feedback and agree that the rationale for the replay experiments was not sufficiently clear in our initial submission. Aligned with the reviewer's earlier suggestion, we have now moved these analyses to the supplementary material (**Extended Data Fig. 12**) and rely on them to provide support for the model. In their revised placement, we use the replay results specifically to validate the switching state space model: after prior plume tracking, flies initially show directed returns, but these decay across bouts when entry angles are no longer consistently coupled to their own actions, exactly as the model predicts.

Fig 5, how meaningful are stats when the model has been tuned to fit data?

Although the model is trained on a subset of the behavioral data (as detailed in the Methods), its performance on the full dataset of flies tracking plumes of varying geometries is not a given. The model relies on a specific set of hypotheses described by 14 parameters and should only be able to capture fly behavior statistics of the full dataset if these hypotheses are valid. For example, models lacking entry-angle memory fail to capture edge-tracking behavior or statistics even when fitted to data (similar to ablation results shown in **Extended Data Fig. 10a,e**). This demonstrates that the statistical agreement we observe is not an inevitable consequence of fitting the model but instead reflects the relevance of the underlying computational assumptions.

5c, is there a difference here between simulation and flies, with flies more efficient, at least for longer bouts?

Thanks for this astute observation. The reviewer is correct that flies appear more efficient than the model for the longest outside bouts. However, this difference is confined to roughly the top 1% of bout durations. Because the vast majority (99%) of real and simulated bouts overlap closely, these rare long bouts appear disproportionately prominent in the former **Fig. 5c**. Notably, these lengthy simulated bouts occur almost exclusively at the very beginning of a trial, when the entry-memory amplitude is close to zero and trajectories resemble correlated random walks, producing long, inefficient excursions. Once entry-angle memory is established after the first plume encounter, simulated trajectories become comparable to those of real flies. Consistent with this, excluding the initial outside bouts—during which both real and simulated flies lack a defined directional memory—substantially eliminates the discrepancy between the two. We have now removed these first trajectories from the analysis and reformatted the plot to better represent the high density of points near the origin. The updated plot is shown in **Fig. 4c**, and the figure legend has been revised accordingly.

A second potential reason why simulated outside trajectories appear less efficient than those of real flies is that the memory-update rate in our model appears to underestimate how rapidly real flies update their entry angle memory. This difference is apparent when comparing the memory update rate in the model versus that inferred for real flies (**Fig. 4g**) during operant conditioning. While the model captures the qualitative pattern of behavior in real flies across successive odor encounters, it updates more slowly. The average learning rate of the model, derived from fitting the trajectories of edge tracking flies in straight plumes, thus appears to be a conservative estimate of the flies' true update dynamics observed in this operant paradigm. One possible reason for this is because we assume a single fixed memory update rate in the model, whereas the update rate of real flies could potentially be dynamically modulated across behavioral contexts (e.g. if the plume structure suddenly changes) or across individuals (e.g. if flies are especially motivated to track)—an interesting possibility that is worth exploring in future studies.

5g seems overly complex, making the same point in three different plots.

We have simplified this figure as suggested and now just compare how the crosswind component of the entry angle memory shifts with successive reinforcements between the model and that inferred for real flies (now **Fig. 4g**) to show how both update.

The model seems to show very minor shift for one encounter (5g), is this really enough to make such a difference in success after one training trial (5h)?

As noted in the legend for **Fig. 5g** and in the Methods, in our operant training paradigm, flies were required to enter the test plume using the same heading in which they had been trained, effectively providing each animal with an additional reinforcement epoch. Therefore, even flies with a single training trial, effectively received two bouts of reinforcement.

Nevertheless, even accounting for this, the reviewer raises an important point about the apparent discrepancy between the model's slower update rate in comparison to real fly behavior, which we comment on above. In the

revised manuscript we now directly compare how the crosswind component of the entry angle memory shifts with successive reinforcements between the model and that inferred for real flies (**Fig. 4f,g**, **Extended Data Fig. 10f**) and explicitly note this difference (lines 390-393). As stated above, this discrepancy may arise from the fact that flies can modulated the rate at which they update memories, something for which we did not have sufficient evidence to put into the model.

Extended data 2, seems large variability across flies? Any comment?

As the reviewer notes, flies show considerable variability in how they edge track—both in how far naïve flies progress (now **Extended Data Fig. 1d,e**) and in how tightly they adhere to the plume boundary (reflected in the distribution of fitted model parameters in **Extended Data Fig. 8d**). One likely contributor to this variation is that edge tracking is strongly state-dependent: starved flies vigorously track an ACV plume, whereas fed flies are largely indifferent to the presence of the plume (data not shown), consistent with prior work demonstrating that satiety-state strongly modulates olfactory behaviors in *Drosophila* (e.g. Krashes et al. *Cell*, 2009; Lewis et al., *Curr Biol*, 2015; Tsao et al., *eLife*, 2018). Although all flies in our study were starved for 16–24 hours, this state-dependence highlights a further layer of modulation that could shape individual differences. A more systematic analysis of how internal state influences edge tracking—and the circuit mechanisms underlying this modulation—will be an interesting direction for future work. We now explicitly note this state dependence in the revised text (lines 579-580).

Extended data 1 and 4, the optogenetic introduces no onset/offset gradient? How does this affect behaviour, and could it be simulated?

The reviewer raises an important point about the distinct temporal dynamics of sensory input as flies track real odor versus optogenetically-produced plumes. Our motivation in performing these optogenetic experiments was to demonstrate that flies can edge track even when sensory neurons from both antennae are synchronously activated without any spatial gradient, suggesting that they do not simply rely on left-right asymmetries to define the boundary of the plume.

Real odor plumes, however, introduce inherent onset and offset delays (on the order of ~500 ms in our paradigm, **Extended Data Fig. 1a,b**) whereas optogenetic activation produces essentially instantaneous stimulation of sensory neurons. In our original submission, we showed that flies edge track under both conditions but we have now expanded our analysis of Orco>CsChrimson trajectories in **Extended Data Fig. 4g**. We show that flies track the edge of optogenetic corridors more tightly on average, with shorter inside and outside excursions. While these differences could arise from differences in the timing of sensory neuron activation, they may also reflect differences in the strength or distribution of activated sensory neurons.

Our model assumes a binary odor signal and therefore must capture odor onset and offset delays and their potential impact on the duration of the leaving and returning states in the values of state transition parameters learned from the behavioral data. In optogenetic experiments, state transition parameters may be slightly different to reflect the quantitative differences in how Orco>CsChrimson flies track the edge. For example, **Extended Data Fig. 8d** shows a fly tracking a real ACV plume that resembles the tight edge tracking displayed by Orco>CsChrimson flies, where the leaving state is shorter than the average fly model, underscoring how variations in edge tracking can arise from different fitted parameters while maintaining the same modeling framework.

A vector-based strategy for olfactory navigation in *Drosophila*
[redacted]

Referee #1
The authors have addressed all the issues raised by this reviewer.
We thank the referee for their constructive input.

Referee #2
This manuscript addresses a question of broad significance for our understanding of sensory-guided navigation in *Drosophila*. The authors have responded to my previous suggestions with care and thoroughness. All the points I raised in my initial report were seriously considered in the revision. Among the new additions, I found particularly exciting the data in Extended Fig. 3 showing the elegant observation that flies accumulate in the region where the slope of the lateral concentration gradient is maximum. This result is consistent with the established sensitivity of *Drosophila* olfactory neurons to concentration derivatives. The inclusion of representative simulated trajectories in Extended Fig. 5d is helpful for appreciating the contrast with real fly behavior. The complementary quantification of behavior in real (ACV) and optogenetic olfactory corridors is also a valuable addition. In response to Reviewer 1, the new experimental evidence supporting the role of FC2 neurons shown in Fig. 6 is solid and strengthens the case for the necessity of these neurons in edge tracking, which complements the EPG silencing results that were already compelling in the initial submission.

Analysis of edge tracking in naturalistic plumes: The authors now test their model in plume structures displaying heterogeneous conditions of turbulence. These simulations highlight the role of entry memory in augmenting navigation success. Using replay of plumes published in past work is a clever strategy. I understand the decision to spatially scale up the dimensions of the plume. Slowing down the playback is indeed a reasonable approach to preserve the overall velocity of odor fluctuations. However, there is no guarantee that the geometric properties of the enlarged plume and the spatiotemporal information a fly collects while moving through it will be comparable to those of the original plume. As the authors themselves note, the spatial and temporal statistics of naturalistic plumes depend on the fluid dynamics of the underlying currents carrying the odor and these are unlikely to be preserved by linear scaling. The reason I raise this limitation is that the authors show virtually no behavior of real flies in the scaled-up plume. While Reviewer Figure 1 suggests that a small number of flies (3) could navigate the plume structure toward its source, more animals would need to be tested to establish this result with confidence. Given that this analysis was elevated to a main figure, I would have expected a more solid comparison between model simulations and the behavior of real flies. Specifically, does the multilayered nature of the navigation strategy deployed by real flies show discrepancies with model predictions? Were real flies generally effective at tracking the scaled-up plume? These questions remain unanswered in the revised manuscript. As a result, the conclusion that flies may deploy multiple navigational strategies depending on plume statistics is left as a hypothesis to be tested in future work. While this is a little unsatisfying, I understand why a complete experimental analysis would go beyond the scope of the current manuscript.

With the caveat noted above regarding the naturalistic plume analysis, I enthusiastically recommend acceptance of this manuscript. As was clear in my initial report, I was highly impressed by the quality of the work and the depth of the questions it addresses. I remain equally impressed by the value and impact of this study after reviewing the revised manuscript. I expect it will be of broad interest to both the navigation and sensory neuroscience communities.

We thank the referee for their enthusiastic support of this work and for the thoughtful comments provided throughout the review process, which have significantly strengthened the manuscript.

We agree that our original analysis of naturalistic plume navigation did not provide a sufficiently direct comparison between model predictions and the behavior of real flies. To address this concern, we have now substantially expanded this dataset (n = 12 flies). As noted in the new **Figure 5** legend and Methods, 10 of 12 flies successfully tracked the naturalistic plume to within 50 mm of the source, demonstrating that flies can robustly navigate this more complex and turbulent odor environment. We have also included **Supplementary Video 4**, which illustrates that even in this naturalistic plume, flies continue to progress along the plume boundary in a manner consistent with the edge-tracking strategy described throughout the manuscript.

In addition, we now show that the predictivity of the inferred memory in real flies increases as animals approach the source, where the plume becomes more stable and less turbulent. This mirrors the behavior of the model and suggests that the same memory-based computations become increasingly informative as plume structure becomes more reliable. Together, these new analyses provide a more direct comparison between model and experimental behavior and support the conclusion that flies effectively navigate the scaled naturalistic plume using strategies that are broadly consistent with model predictions.

While we agree that understanding whether flies flexibly deploy additional navigational strategies under different plume statistics remains an important direction for future work, we believe the new experimental data substantially strengthen our central conclusion that the edge-tracking strategy identified here remains effective even in complex and dynamic plume environments.

Referee #3 (Remarks to the Author):

I thank the authors for their very thorough consideration of and response to the reviews. The additions and clarifications have strengthened an already excellent paper and I have only a few minor points for consideration:

We appreciate the referee's thoughtful input over both rounds of review.

Extended data figure 1 caption, has b) twice, the second one should be e)

Thanks for catching this—we have fixed it.

Fig 2 i & j, in the text these results show "flies continued to walk in the same direction they had previously used to return to the plume" for "10s of seconds". It seems apparent in these plots that at 10 seconds, the flies are indeed walking very close to the same direction, but after that they start to diverge (e.g. the 45 degree flies walk closer to the expected 90 degree direction than the 90 degree flies by 30s, although still in the same general direction). Maybe this could be commented on, as indicative of possible memory decay rate?

We agree with the reviewer that trajectories become more variable with increasing time after plume offset, although are still distinct from the generally upwind bias flies show prior to edge tracking. We have therefore revised the text to state simply that flies continued to walk in the same general direction they had previously used to return to the plume, rather than emphasizing a specific timescale. Although the data are consistent with a gradual weakening of this directional bias over time, we do not think the present experiments provide a sufficiently quantitative measure of memory decay and have therefore avoided making a stronger claim.

Finally, I feel the authors could actually strengthen the point made in this paper if they did not, from the start, use 'navigation' as a broad term inclusive of any type of goal-directed behaviour (including reflexive steering), and instead made the distinction that 'navigation' requires “movement towards a desired goal that cannot be continuously sensed” - as they demonstrate occurs here for the fly. It seems counterproductive to dilute terminology that, if used more selectively, can usefully distinguish certain types of behaviour from others based on the computational demands required.

We thank the reviewer for this suggestion. We have carefully considered the distinction between behaviors guided by immediate sensory cues and those that require memory, and modified the Introduction accordingly:

“In many instances, a simple chain of sensorimotor reflexes can be used to guide animals towards or away from a salient object using only immediate sensory cues[1,7]. However, when sensory signals are intermittent or unreliable, animals may instead need to navigate toward goals that cannot be continuously sensed, requiring memory of their position or direction.”
